# A Platform for Site‐Specific DNA‐Antibody Bioconjugation by Using Benzoylacrylic‐Labelled Oligonucleotides

**DOI:** 10.1002/anie.202109713

**Published:** 2021-11-03

**Authors:** Juraj Konč, Libby Brown, Daniel R. Whiten, Yukun Zuo, Peter Ravn, David Klenerman, Gonçalo J. L. Bernardes

**Affiliations:** ^1^ Yusuf Hamied Department of Chemistry University of Cambridge Lensfield Road Cambridge CB2 1EW UK; ^2^ AstraZeneca R&D BioPharmaceuticals Unit|Antibody Discovery & Protein Engineering (ADPE) Milstein Building, Granta Park Cambridge CB21 6GH UK; ^3^ UK Dementia Research Institute University of Cambridge Cambridge CB2 0XY UK; ^4^ Instituto de Medicina Molecular João Lobo Antunes Faculdade de Medicina Universidade de Lisboa Avenida Professor Egas Moniz 1649-028 Lisboa Portugal; ^5^ Current address: Department of Biotherapeutic Discovery H. Lundbeck A/S Ottiliavej 9, 2500 Valby Denmark

**Keywords:** antibodies, bioconjugation, imaging, mass spectrometry, nucleic acids

## Abstract

Many bioconjugation strategies for DNA oligonucleotides and antibodies suffer limitations, such as site‐specificity, stoichiometry and hydrolytic instability of the conjugates, which makes them unsuitable for biological applications. Here, we report a new platform for the preparation of DNA‐antibody bioconjugates with a simple benzoylacrylic acid pentafluorophenyl ester reagent. Benzoylacrylic‐labelled oligonucleotides prepared with this reagent can be site‐specifically conjugated to a range of proteins and antibodies through accessible cysteine residues. The homogeneity of the prepared DNA‐antibody bioconjugates was confirmed by a new LC‐MS protocol and the bioconjugate probes were used in fluorescence or super‐resolution microscopy cell imaging experiments. This work demonstrates the versatility and robustness of our bioconjugation protocol that gives site‐specific, well‐defined and plasma‐stable DNA‐antibody bioconjugates for biological applications.

## Graphical abstract

BA‐PFP is a simple and practical reagent that enables preparation of a range of diverse site‐specific, homogenous DNA‐protein/antibody bioconjugates under mild conditions. The generated constructs can be conveniently characterized by an optimized LC‐MS method and utilized as probes for cell imaging using fluorescence or super‐resolution DNA‐PAINT microscopy.

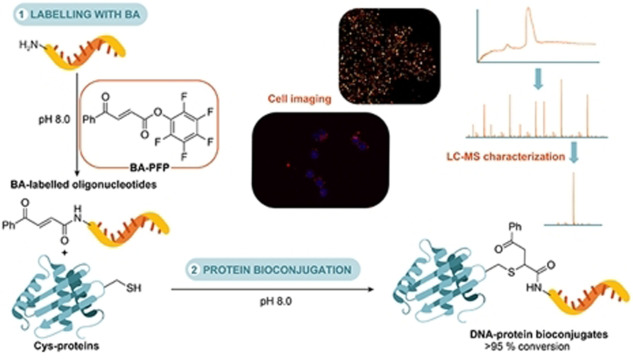

## Introduction

Cross‐linking of various types of molecules leading to chimeric constructs with combined functionalities has been gaining attention across the fields of chemical biology, biotechnology, and medicine.^[1]^ Among these, proteins are one of the most important and valuable class of biomolecules owing to the wide range of functions they fulfil in living organisms. Numerous studies have been undertaken to extend the diversity of protein functionalities by introducing non‐natural labels or “tags” in the emerging field of chemical protein modification.^[1a]^ Besides proteins, nucleic acids (NAs) are also an extensively studied class of biomolecules. It is mainly the highly specific and predictable base pairing that makes DNA or RNA oligonucleotides (ONs) unique tools for the design of biomolecular hybrids with new structures and improved functions. ONs have been applied in the design of DNA‐microarrays,^[2]^ other nanostructures,^[3]^ imaging^[4]^ and as therapeutic agents.^[5]^ By joining proteins and NAs in a single biomolecular scaffold, NA‐protein hybrids can combine the programmable sequence recognition properties of NAs and diverse functionalities of proteins. Two major research areas that benefit from the dual functionality of NA‐protein bioconjugate constructs are bioanalysis and nanofabrication.^[6]^ In addition, conjugation of ONs to antibodies has been used for the antibody‐mediated delivery of therapeutic ONs,^[7]^ cytotoxic agents through intercalation^[8]^ or hybridization of complementary DNA ONs,^[9]^ decoration of DNA nanostructures with multiple proteins^[10]^ and fluorophores^[11]^ and DNA‐antibody bioconjugates have been used as probes for super‐resolution imaging by means of the DNA‐PAINT method.^[12]^

**Figure 1:**
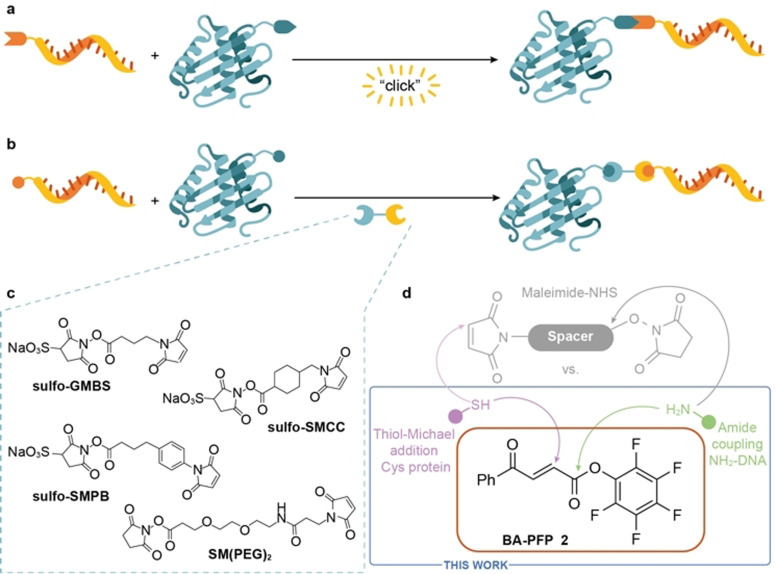
Strategies for the preparation of covalent NA‐protein bioconjugates. a) Conjugation strategy based on the introduction of handles for bioorthogonal ligation reactions. b) Conjugation of ONs and native proteins by heterobifunctional linkers. c) Examples of heterobifunctional linkers bearing various spacers commonly used for the DNA‐protein bioconjugation. d) Conjugation method with novel BA‐PFP reagent **2** described in this work.

**Figure 2:**
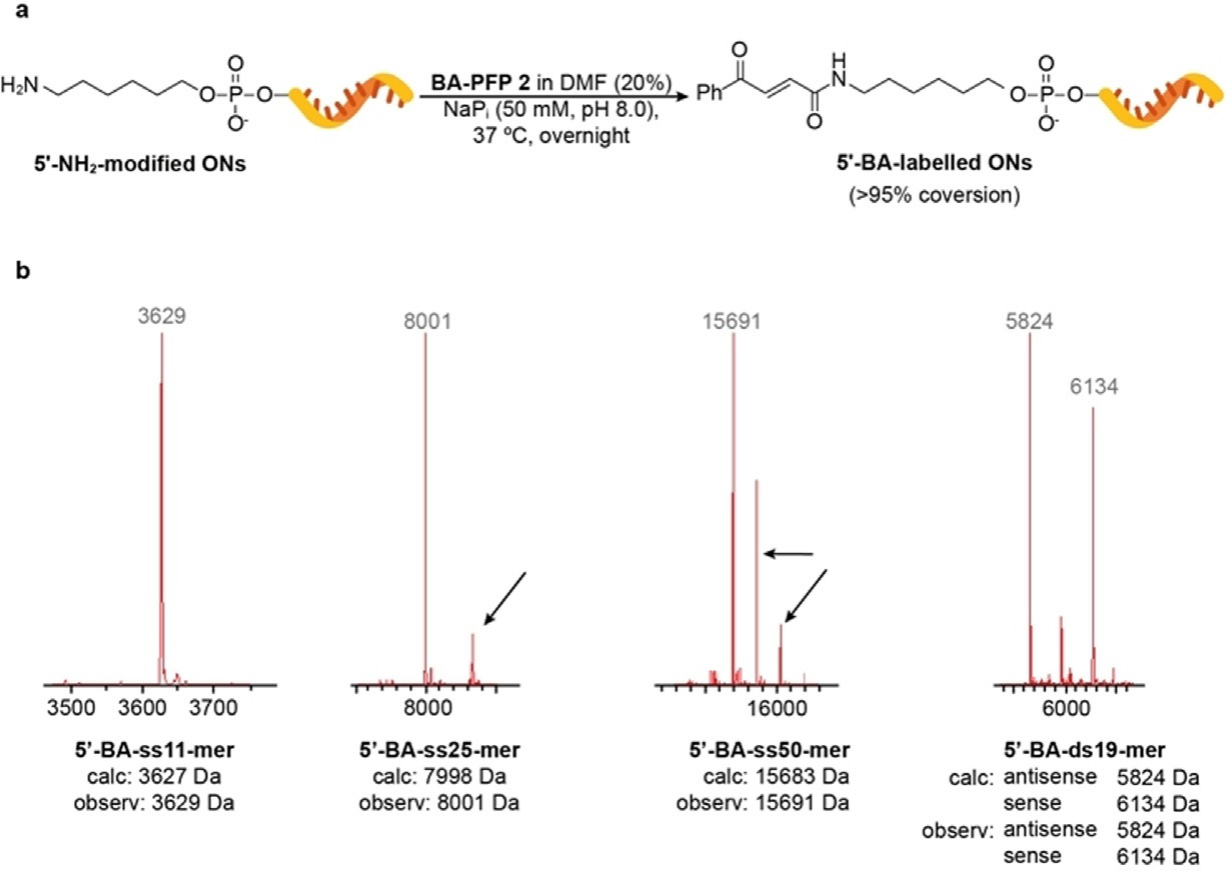
Modification of 5′‐amino DNA ONs and LC‐MS characterization of BA‐labelled products. Deconvoluted mass spectra show peaks with masses expected for ss11‐mer, ss25‐mer, ss50‐mer and ds19‐mer 5′‐BA‐labelled products. Extra peaks (as indicated by black arrows) seen with longer ONs (ss25‐mer and ss50‐mer) correspond to hexaflufluroisopropanol adducts (+168 Da) formed during electrospray ionization. For full LC‐MS spectra see Figures S20–S23.

However, for constructs like DNA‐antibody conjugates to become viable for bioanalytical and biological applications, efficient methods for their production are needed. Many different chemical approaches are available to prepare these bioconjugates.^[13]^ The choice of conjugation method usually depends on the scaffold of the protein of interest, and the main challenges are scalability, control over efficacy, site‐specificity and stoichiometry of the NA bioconjugation. Strategies for the preparation of covalent DNA‐antibody conjugates can be divided into two main categories: 1) approaches based on proteins modified with functional groups suitable for biorthogonal reactions, such as strain‐promoted azide–alkyne cycloaddition^[14]^ or inverse electron‐demand Diels–Alder reactions[^14c^, ^15^] (Figure 1 a) or 2) direct conjugation of modified ONs to native proteins by using bifunctional cross‐linkers (Figure 1 b).^[16]^ Whereas the former requires either genetic manipulation of the protein of interest or additional steps to introduce the functional groups for the biorthogonal ligation, the latter cross‐links the reactive amino acid residues on the protein surface with modified ONs directly. However, proteins usually contain multiple reactive residues, which often results in nonspecific labelling at sites important for protein activity and/or binding. To avoid formation of inactive conjugates, site‐specific bioconjugation of NAs to proteins with control over modification site is required; often attempted by using bifunctional cross‐linkers. Functionalized homo‐ or hetero‐bifunctional linkers usually contain reactive activated esters for amine modification or Michael acceptor moieties for conjugation of thiol bearing molecules (Figure 1 c). *N*‐hydroxysuccinimide (NHS) ester and maleimide moieties are some of the most frequently used scaffolds but various other crosslinkers are available.^[16]^ Although popular, bifunctional linkers based on NHS and maleimide functionalities have some drawbacks. Maleimide is widely used for reactions with thiol‐containing molecules but the selectivity of maleimide towards thiols highly depends on the reaction pH. At neutral pH, thiols react with maleimide 1000‐times faster than amines but at higher pH reaction with amine is favored.^[17]^ This non‐specific labelling can lead to mixtures of heterogenous conjugates with various characteristics. Furthermore, at more alkaline pH, hydrolysis of both the NHS ester and maleimide occurs, which results in an unreactive carboxylic acid and maleamic acid, respectively.^[16]^ Another disadvantage of the maleimide reagents is the limited stability of the formed thioether bond, which can undergo a rapid thiol‐exchange reaction.[^17^, ^18^] To avoid some of the downsides of these reagents, improved conjugation techniques that give site‐specific, homogenous and well‐defined DNA‐antibody conjugates are desired. In this work, we report a strategy that leads to chemically defined constructs by employing new benzoylacrylic acid pentafluorophenyl ester (BA‐PFP) labelling reagent **2** (Figure 1 d) as an alternative to the cross‐linking of ONs and proteins by conventional maleimide‐NHS ester‐based reagents. Importantly, we use the DNA‐antibody conjugates generated as probes in fluorescence microscopy and super‐resolution DNA‐PAINT imaging experiments.

## Results and Discussion

Our approach towards the preparation of DNA‐antibody conjugates was based on the method for cysteine protein modification using benzoylacrylic (BA) reagents recently developed in our laboratory.^[19]^ With these reagents (sometimes in stoichiometric amounts) proteins with solvent‐accessible cysteine residues are irreversibly modified with reaction kinetics comparable to maleimide chemistry.^[19a]^ This approach offers site‐specific, stable constructs that overcome the disadvantages of maleimide bioconjugates, such as hydrolytic instability of reagents and problems with retro‐Michael addition reaction.^[19a]^ To introduce the BA moiety to DNA ONs, we used bifunctional reagent **2**, which is easily prepared by activation of commercially available trans‐3‐benzoylacrylic acid (**1**) (see Organic synthesis of BA reagents in the SI). PFP‐activating ester group was chosen for its known higher hydrolytic stability over NHS‐ester derivatives.^[16]^ Bifunctional reagent **2** can be stored as a solid for months or in solution for weeks and used without any significant loss of the reactivity for the modification of various amino group‐containing small molecules or biomolecules (see Organic synthesis of BA reagents in the SI).

A small optimization of the DNA‐labelling reaction was performed on the commercially available short single‐stranded (ss) DNA ON 5′‐NH2‐ss11‐mer that contains 11 nucleotides and a 5′‐amino modifier (see 5′‐NH_2_ DNA ON BA‐labelling reaction optimization in the SI). Conditions that used different amounts of reagent and dimethyl formamide (DMF) were screened. The best results were obtained with 100 equiv of **2** in 20 % DMF in phosphate (NaP_i_) buffer at pH 8.0 and 37 °C overnight (Table S1). Under these conditions, complete conversion of the starting ON into BA‐labelled ON (determined by LC‐MS) was obtained. Lower amounts of linker and shorter reaction times did not lead to complete conversions and more DMF or linker resulted in a small fraction (<10 %) of the second DNA modification, probably on one of the nucleophilic sites on nucleobases, being detected. The second DNA modification was also noted after prolonged reaction times (>48 h) and under more basic conditions (NaP_i_ pH 10.0 buffer for 24 h). Labelling of amino‐modified DNA ONs by **2** can be efficiently performed on different reaction scales (20 μL–1 mL) and at a range of ON concentrations (25–100 μM). BA‐labelled ONs can be easily purified by using size‐exclusion spin columns for small‐scale preparations or RP‐HPLC for larger‐scale labelling reactions.

The optimized labelling conditions were used to label four amino‐modified ONs, 5′‐NH2‐ss11‐mer, 5′‐NH2‐ss25‐mer, 5′‐NH2‐ss50‐mer with different lengths, including double‐stranded (ds) DNA‐ON 5′‐NH2‐ds19‐mer (Figure 2). We chose examples of modified ONs based on their possible applications: shorter (ss11‐mer) ONs are usually used in preparation of probes for super‐resolution microscopy by DNA‐PAINT,^[12]^ whereas longer ones are in the range of sizes used for therapeutic^[7]^ (ss25‐mer or ds19‐mer) or biomedical^[3]^ (ss50‐mer) applications. All BA‐labelled ONs were successfully characterized by LC‐MS (Figure 2b) and in all cases, complete conversion of the starting material into expected product was observed. It is necessary to mention that for modification of 5′‐NH2‐ss50‐mer more linker **2** (200 equiv instead of 100 equiv) was needed to ensure complete conversion of the starting ON (Figure S22).

In a similar manner, 5′‐NH2‐ss11‐mer and 5′‐NH2‐ss25‐mer ONs were modified with commercially available 3‐(maleimido)propionic acid *N*‐hydroxysuccinimide ester **5** using the optimized BA labelling conditions stated above. Complete conversion of the starting material to either the maleimide (minor) or hydrolyzed maleamic acid (major) labelled product was observed by LC‐MS for both reactions (Figures S25 and S26). RMss11 (1:9 5′‐maleimide‐ss11‐mer:5′‐maleamic acid‐ss11‐mer) was then incubated at 37 °C to test hydrolytic stability of the maleimide‐labelled ON. After 24 h, complete hydrolysis of 5′‐maleimide‐ss11‐mer to 5′‐maleamic acid‐ss11‐mer (Figure S27) was obtained. In comparison, no hydrolysis of 5′‐BA‐ss11‐mer was observed under the same conditions (Figure S29), highlighting the remarkable hydrolytic stability of the BA‐labelled ONs.

With a well‐working protocol for the labelling of amino‐modified ONs, we wanted to proceed with the bioconjugation of BA‐labelled ONs to proteins and antibodies. Because we were interested in developing a protocol for the generation of site‐specific and stoichiometric (1:1) DNA‐protein conjugates, we investigated an analytical method for the precise characterization of these constructs. Bioconjugation reactions between ONs and proteins are typically analyzed by gel electrophoresis under reducing or non‐reducing conditions.[^7a^, ^20^] Although this analytical method is useful to confirm successful bioconjugation reaction has taken place, its accuracy and resolution is inferior to results obtained by mass spectrometry (MS), which is nowadays the prevailing analytical tool for structural characterization of bioconjugates. Accuracy of characterization is needed for applications that require use of well‐defined and homogenous conjugates, for example development of improved therapies^[21]^ or single‐molecule techniques in biophysics.^[22]^ One of the earliest examples of DNA‐protein hybrids characterization by MS involved bioconjugates that were enzymatically pre‐digested and then analyzed by LC‐MS/MS.^[23]^ Besides this, only a handful of reports on the characterization of DNA‐protein bioconjugates by MS methods have been published. These include use of matrix‐assisted laser desorption/ionization MS that usually requires specific sample and matrix preparation.^[24]^ Electrospray ionization (ESI) MS was used to characterize NA‐protein conjugates but gave low mass accuracy and errors in mass ranges of tens of Da.[^7b^, ^25^] Since MS provides more precise results than gel electrophoresis, we believed that a liquid chromatography‐mass spectrometry (LC‐MS) method for the accurate characterization of DNA‐protein bioconjugates could be developed. Analysis by LC‐MS is well‐established for the accurate characterization of bioconjugates with small molecules, however, its use for the analysis of DNA‐protein conjugates is not that straightforward. To the best of our knowledge a simple, accurate, reproducible and reliable LC‐MS method for routine characterization of intact DNA‐protein hybrids has not been reported.

**Figure 3:**
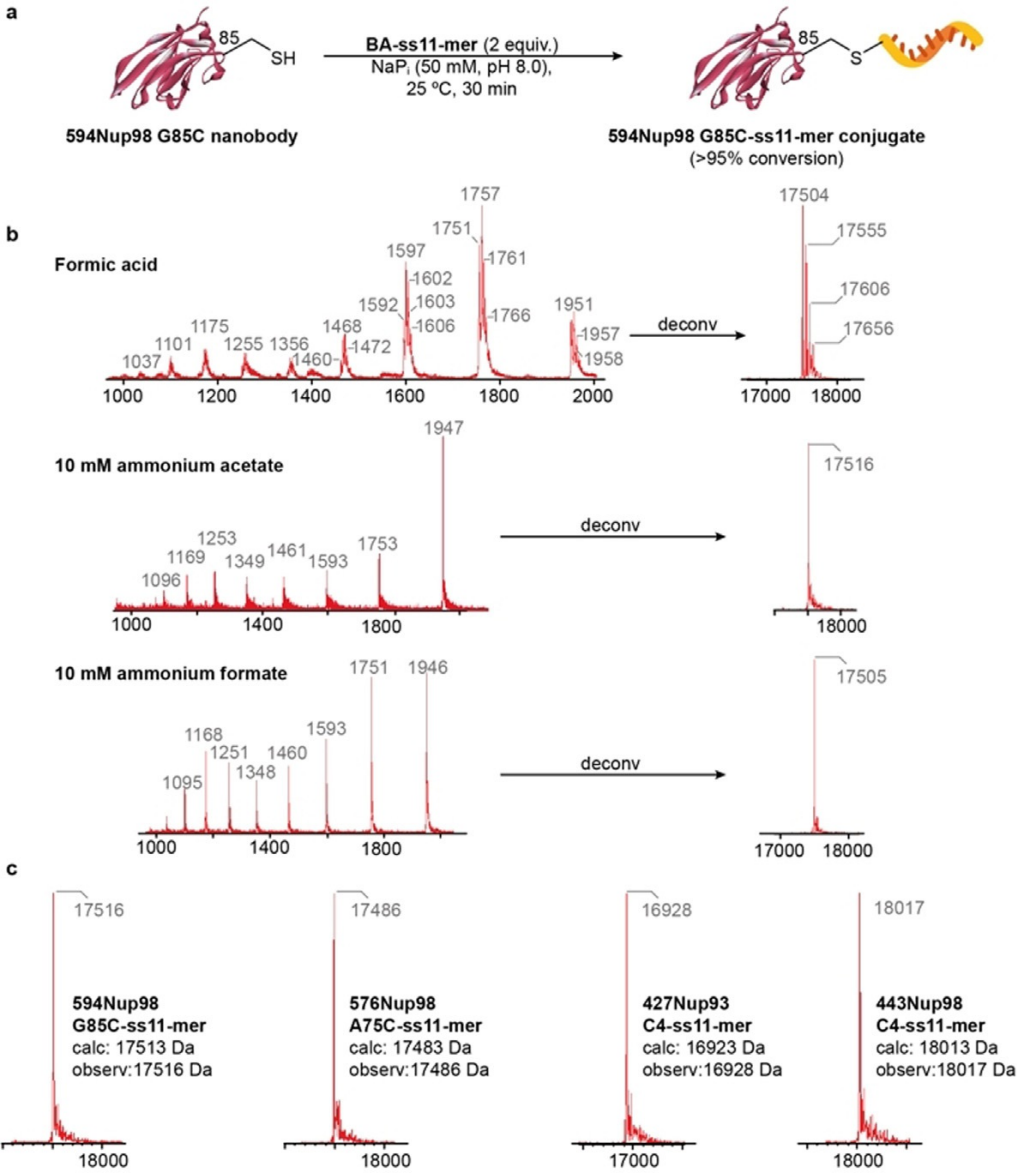
Optimization of the LC‐MS method for the characterization of the model DNA‐antibody conjugate. a) Conditions used for the preparation of anti‐nucleoporin nanobody conjugates with a short ss11‐mer DNA ON. b) Comparison of the effect of different LC mobile phase additives on the ion series and deconvoluted mass spectra for conjugate 594Nup98 G85C‐ss11‐mer. c) LC‐MS characterization of prepared ss11‐mer conjugates of different anti‐nucleoporin nanobody variants with deconvoluted mass spectra and masses that correspond to expected products. For full LC‐MS spectra see Figures S30–S40.

Bioconjugate 594Nup98 G85C‐ss11‐mer was chosen as a model for the optimization of the LC‐MS method. The conjugate was prepared by reaction of a short BA‐labelled ON 5′‐BA‐ss11‐mer with the single‐domain antibody against *Xenopus* nucleoporin complex 594Nup98 G85C^[26]^ bearing a single available cysteine residue (Figure 3 a). Complete conversion of the starting protein was obtained under mild reaction conditions (pH 8.0, 25 °C, 2 equiv of the BA‐labelled ON; Figure S30). At first, this bioconjugation reaction was analyzed by standard LC‐MS conditions suitable for proteins with formic acid as the LC‐MS additive (Figure 3 b). Under these conditions, ion series with multiple ion species was obtained, which was deconvoluted by the MaxEnt1 algorithm and led to a peak with a molecular weight that corresponded to the expected product (ca. 17.5 kDa). This was observed together with other ions with higher masses (Figure 3 b), which were assigned to ion species with different levels of saturation with counter cations, such as Na^+^ and K^+^ on the phosphate backbone of the ON part of the bioconjugate. We assumed that this was due to the phosphate buffer used in the labelling reaction or the mobile phase used for the LC‐MS analysis. So, to eliminate these species, we tested whether a different LC‐MS mobile phase additive would provide better results. With ammonium acetate^[27]^ at 10 mM concentration, much cleaner extracted ion series were obtained relative to experiments that used formic acid as the LC‐MS additive (Figure 3 b) and deconvolution led to a single peak with the mass corresponding to the calculated mass of the desired conjugate (17 516 Da). This was probably due to the saturation of the ON phosphate backbone by volatile ammonium counter cations and overall stabilization of the charge of the DNA‐protein conjugate sample. An ion series of an individual species could be then extracted and deconvoluted into a mass spectrum that contained a single peak for the expected product. Concentration of LC‐MS additive had little effect on the signal of the extracted ion series or deconvoluted mass spectra at higher (10 mM) or lower (5 mM, 2 mM or 1 mM) ammonium acetate concentrations (Figures S32–S35). However, the use of higher concentrations was noticeable in the UV trace of the LC whereby background noise was detected. To examine the impact of the counter anion of the LC‐MS additive on the analysis, 10 mM ammonium formate was also tested. We observed a clear effect on the ionization of the sample, but the deconvolution and reconstruction of the total mass ion gave a similar result as with 10 mM ammonium acetate (Figure 3 b). At 1 mM concentration, extracted ion series clearly show differences in the presence and intensity of ion species, which we attributed to the less efficient ionization by ammonium formate at lower concentration (Figure S36). Based on all these observations, we chose 1–10 mM ammonium acetate as the LC‐MS additive for the accurate analysis and characterization of our DNA‐antibody conjugates. We then tested the applicability and accuracy of our LC‐MS method for the characterization of 11‐mer conjugates with other variants of *Xenopus* nucleoporin nanobodies.^[26]^ Constructs 576Nup98 A75C‐ss11‐mer, 427Nup93 C4‐ss11‐mer and 443Nup98 C4‐ss11‐mer were prepared similarly to 594Nup98 G85C‐ss11‐mer conjugate (Figures S38–S40). In all the cases, our optimized LC‐MS method gave accurate results for the deconvoluted mass spectra of prepared conjugates with observed masses in the range of ±5 Da from calculated values (Figure 3 c). The advantages of site‐specificity and reproducibility of the conjugation reaction combined with the accuracy of our LC‐MS analysis could allow application for the construction of well‐defined, functionally active DNA‐protein conjugates.

**Figure 4:**
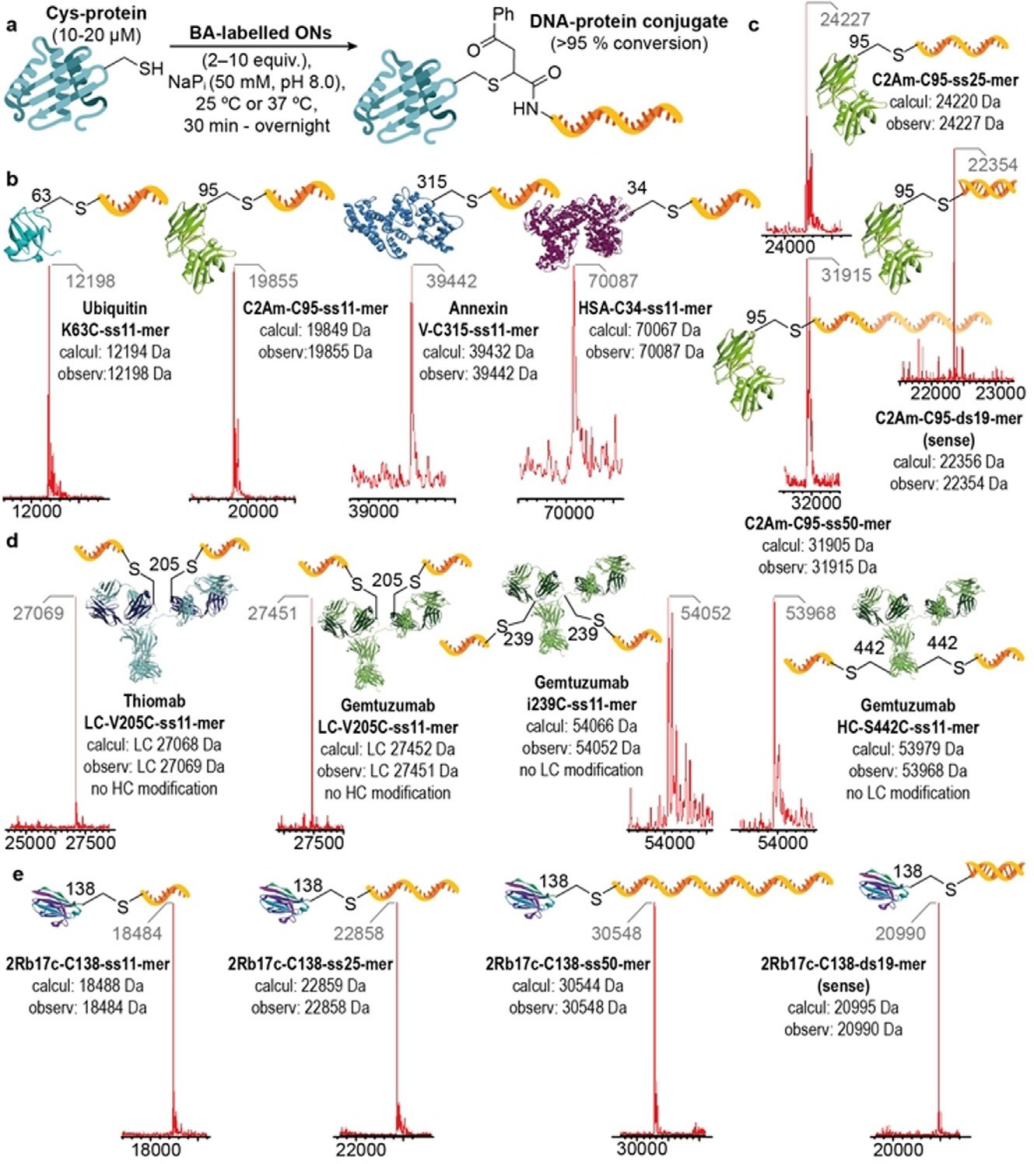
Site‐specific NA‐antibody conjugates. a) Scope of the conjugation of BA‐modified ONs through cysteine residues to proteins and antibodies of various sizes and formats. b) Deconvoluted mass spectra obtained from the modification of four model proteins with the 5′‐BA‐ss11‐mer ON. c) Deconvoluted mass spectra obtained from the modification of C2Am‐C95 with different BA‐modified ONs. d) Deconvoluted mass spectra of prepared DNA‐antibody conjugates that bear antibodies against HER2 or CD33 receptors. To observe individual LC and HC of IgG mAbs, disulfide bonds between LCs and HCs were reduced prior to LC‐MS analysis (SI). e) Deconvoluted mass spectra of four different 2Rb17c‐C138 conjugates. Each DNA‐protein bioconjugation reaction was performed under the optimized conditions (SI) at least two times and led to identical, pure products. For full LC‐MS spectra see Figures S41, S43–S52, S54, S56–59.

Having optimized the conditions for the LC‐MS analysis and characterization of the DNA‐antibody conjugates, we wanted to explore the applicability of our bioconjugation protocol to proteins of various sizes with cysteine residues with different accessibility and reactivity. We managed to successfully conjugate 5′‐BA‐ss11‐mer to four different proteins including Ubiquitin K63C (with an engineered cysteine residue),^[28]^ C2A domain of Synaptotagmin‐I C2Am‐C95 (derivatives of which are used as probes for in vivo imaging of apoptosis),^[29]^ Annexin V‐C315^[30]^ and an recombinant human serum albumin‐Recombumin (Albumedix Ltd) HSA‐C34^[31]^ (which often serves as a versatile carrier for therapeutic and diagnostic agents). In all cases, bioconjugation conditions were optimized to obtain complete conversion of the starting protein into desired bioconjugate with no more than 10 equiv of BA‐labelled ON used (Figure 4 a). All reactions were performed in 50 mM NaP_i_ buffer at pH 8.0, 25 °C, 30 min or 1 h to obtain UbK63C‐ss11‐mer, C2Am‐C95‐ss11‐mer and HSA‐C34‐ss11‐mer conjugates (Figure 4 b). Annexin V‐315‐ss11‐mer conjugate required a reaction temperature of 37 °C and a prolonged reaction time because of the more buried and consequently less reactive Cys‐315 residue (Figure S44). Moreover, by conjugating longer ssONs 5′‐BA‐ss25‐mer, 5′‐BA‐ss50‐mer and even dsON 5′‐BA‐ds19‐mer to C2Am‐C95 (Figure 4 c) we have shown that our DNA‐protein bioconjugation protocol is versatile and suitable for conjugation of different types of BA‐labelled ONs. As a control experiment, we mixed Ubiquitin K63C with an unmodified 5′‐NH2‐ss11‐mer (Figure S42). Formation of conjugate was not observed under these conditions, which demonstrates that unspecific bioconjugation reactions do not occur in the absence of the BA moiety. All the prepared DNA‐protein bioconjugates were successfully characterized by our optimized LC‐MS method thus proving its reliability and accuracy in the analysis of DNA‐protein constructs for a wide range of masses and types of bioconjugates.

Next, we extended our DNA bioconjugation method to antibodies. Site‐specific and stoichiometric conjugation of DNA or RNA ONs to monoclonal antibodies (mAbs) or antibody fragments is desired for applications like structural DNA nanotechnology and imaging,^[32]^ preparation of well‐defined DNA‐antibody nanostructures^[33]^ or delivery of therapeutic ONs, such as antisense ONs (AOs)^[7a]^ or small‐interfering RNAs (siRNAs).^[7b]^ As only a limited number of bioconjugation methods for such constructs are available, we wanted to test the reliability of our method in the preparation of ON‐antibody conjugates. With our bioconjugation platform, light (LC) and heavy chain (HC) subunits of immunoglobulin G (IgG) mAbs can be easily modified with BA‐labelled ONs via engineered cysteine residues. To demonstrate this we used Thiomab LC‐V205C,^[34]^ a mAb targeting the human epidermal growth factor receptor 2 (HER2) that is over‐expressed on the cell surface of certain types of breast cancer, and Gemtuzumab mAb variants, which recognize the myeloid differentiation antigen CD33 on acute myeloid leukemia (AML) cells.^[35]^ By using our bioconjugation protocol, cysteine residues on LCs of Thiomab LC‐V205C and Gemtuzumab LC‐V205C and cysteine residues on HCs of Gemtuzumab i239C and Gemtuzumab HC‐S442C were the only amino acid residues modified with 5′‐BA‐ss11‐mer ON (Figure 4 d). For the bioconjugation reaction conditions we used 5 equiv of the BA‐labelled ON per cysteine residue in 50 mM NaP_i_ buffer at pH 8.0 and 37 °C for 1 h, which led in all cases to complete conversion of starting antibody materials into desired bioconjugates. To rule out any chemoselectivity issues, 5′‐BA‐ss11‐mer was also reacted with Trastuzumab, a HER2 targeting mAb containing no additional accessible cysteine residues. LC‐MS analysis revealed no modification in the light or heavy chain after 1 h at 37 °C (identical results were obtained when leaving the reaction for 48 h; Figure S53), demonstrating the site‐specificity of the BA‐labelled ON reagents towards cysteine residues. It is worth noting that LC‐MS analysis on ON‐modified HC antibody fragments was challenging. As seen in Figure 4 d (see also Figures S51 and S52), the signal of the main peak in the deconvoluted mass spectra of HCs of the ss11‐mer‐modified Gemtuzumab i239C and Gemtuzumab HC‐S442C variants is of lower intensity and accompanied by other peaks. Although with a slightly larger error, observed peaks are in the range of expected masses for bioconjugate products, which suggests the DNA‐antibody conjugation reaction was successful. The inaccuracy in the LC‐MS analysis for this type of conjugate could be attributed to their inefficient ionization caused by glycosylation of mAb with various glycans attached to HCs.^[36]^

In addition to full‐length IgG mAbs, we have applied our DNA‐protein bioconjugation protocol to single domain anti‐HER2 antibody 2Rb17c‐C138^[37]^ with all four prepared BA‐labelled ONs (Figure 4 e). Here, complete conversion was observed and all products were characterized by LC‐MS with masses as expected. The range of BA‐labelled ONs of various lengths successfully conjugated to an array of various proteins and antibodies clearly demonstrates the versatility of this simple and powerful, bioconjugation method to give site‐specific and stoichiometric DNA‐protein conjugates. 2Rb17c‐C138 was also reacted with RMss11 (1:9 5′‐maleimide‐ss11‐mer:5′‐maleamic acid‐ss11‐mer) under the same conditions as those used for 5′‐BA‐ss11‐mer. Only ca. 64 % conversion of the starting material to the desired conjugate was observed (Figure S55). As a control, 2Rb17c‐C138 was also reacted with 5′‐maleamic acid‐ss11‐mer and as expected, formation of conjugate did not occur (Figure S28). The low conversion of 2Rb17c‐138 to 2Rb17cc‐C138‐ss11‐mer using RMss11 can be attributed to the high percentage of 5′‐maleamic acid‐ss11‐mer present in the reaction mixture. Altogether, the results of both antibody bioconjugation and ON labelling experiments confirm the superior performance of the BA functionality over maleimides in DNA‐antibody bioconjugation reactions.

**Figure 5:**
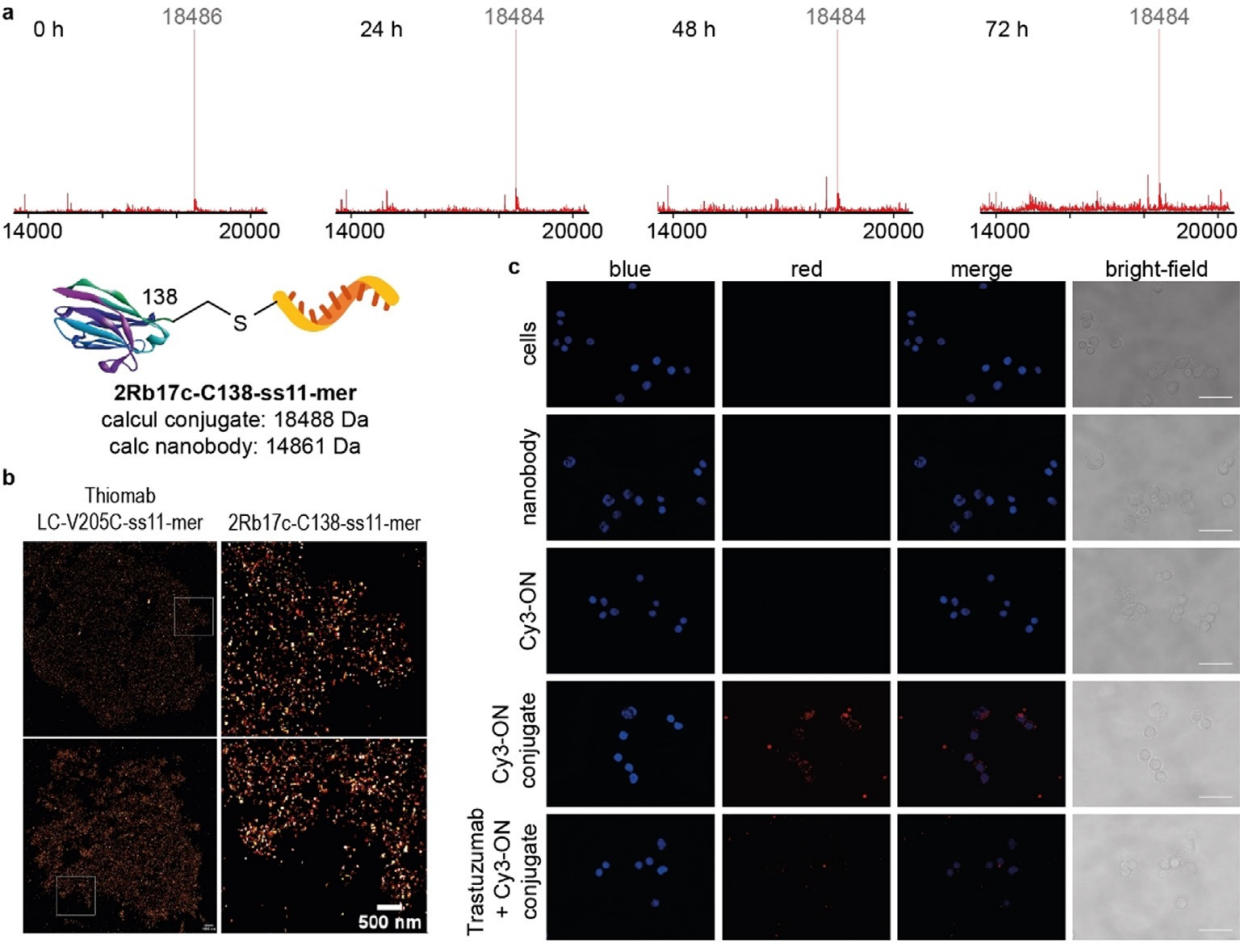
Bioconjugate integrity studies and cell imaging experiments. a) LC‐MS based conjugate integrity study of the anti‐HER2 nanobody conjugate 2Rb17c‐C138‐ss11‐mer in the presence of 10 % human plasma with deconvoluted mass spectra of samples taken at specified time points. No detectable degradation of the DNA‐nanobody conjugate was observed and an intact 2Rb17c‐C138‐ss11‐mer conjugate was identified by LC‐MS after 72 h. For full LC‐MS spectra see Figures S60–S63. b) Super‐resolution images of HER2 receptors on SKBR3 breast cancer cells obtained by DNA‐PAINT method. 2Rb17c‐C138‐ss11‐mer and Thiomab LC‐V205C‐ss11‐mer were used as probes. Scale bars represent 1000 nm (top images) or 500 nm (bottom images). c) Epifluorescence microscopy imaging of the HER2 receptor‐mediated internalization of the 2Rb17c‐C138‐Cy3‐3′‐ss25‐mer conjugate on SKBR3 breast cancer cells. Obtained images of SKBR3 cells incubated for 2 h: without any probe, with 2Rb17c‐C138 antibody, 5′‐NH2‐Cy3‐3′‐ss25‐mer ON, 2Rb17c‐C138‐Cy3‐3′‐ss25‐mer or with 2Rb17c‐C138‐Cy3‐3′‐ss25‐mer after 1 h incubation with Trastuzumab. Images were processed using ImageJ software, scale bars represent 100 mm. Experiments were performed two independent times. Representative data from one experiment is shown. For full experimental details see SI.

As we were keen to develop a bioconjugation method which provides DNA‐antibody conjugates suitable for in vivo studies, we assessed the plasma stability of 2Rb17c‐C138‐ss11‐mer. The conjugate was added to 10 % human plasma in PBS buffer pH 7.4 at 37 °C, and samples were taken at four different time points (0 h, 24 h, 48 h and 72 h) and analyzed by LC‐MS (Figure 5 a). No decomposition of 2Rb17c‐C138‐ss11‐mer was seen, which strongly suggests that it is stable in the presence of human plasma and opens up opportunities for this method to be used for the preparation of hybrid biomolecular constructs for in vivo applications, such as improved targeted cancer therapy by antibody‐mediated delivery of siRNA, antisense ONs or other therapeutic ONs.^[38]^

Ultimately, Thiomab LC‐V205C‐ss11‐mer and 2Rb17c‐C138‐ss11‐mer conjugates were used as probes in super‐resolution microscopy experiments with the DNA‐PAINT method.[^12^, ^14b^, ^24c^] The sequence of the conjugated ON,^[39]^ called “docking strand”, is designed to be complementary to another short 10‐mer fluorophore‐labelled DNA ON, referred to as “imager strand”. The low melting temperature of the corresponding duplex causes the “imager strand” to repeatedly bind and dissociate from the target “docking strand” and this transient binding enables to locate “docking strand”‐labelled antibody. The detected “blinking” is then used to reconstruct the super‐resolution image. Both Thiomab LC‐V205C full‐length IgG and 2Rb17c‐C138 single‐domain antibody selectively bind to HER2 receptors on breast cancer cells, so we investigated whether HER2 receptor binding of both bioconjugate probes was retained even after DNA ON conjugation. These experiments were performed on paraformaldehyde‐fixed SKBR3 breast cancer cells (see DNA‐PAINT imaging experiments in the SI) which are known for their high HER2 receptor expression levels.^[40]^ High localization numbers were detected in both cases and super‐resolution images of HER2 receptor networks on the surface of SKBR3 cells were acquired (Figure 5 b). Results obtained from these experiments confirm that both prepared DNA‐antibody conjugates retained their target receptor binding activity.

To provide further insight into intracellular trafficking and localization of generated bioconjugates, another experiment which provides qualitative evidence on the receptor‐mediated internalization of the DNA‐antibody bioconjugate was carried out by using epifluorescence microscopy. Internalizing anti‐HER2 single‐domain antibody 2Rb17c‐C138, which upon binding to its target cell surface HER2 receptors is taken up by cells via receptor‐mediated endocytosis, was chosen as delivery vehicle. This feature together with the smaller format of this antibody relative to full‐length anti‐HER2 IgG Thiomab LC‐V205C, makes this antibody an ideal candidate for cell‐specific (in this case HER2+), antibody‐mediated delivery of drugs and therapeutic ONs. 2Rb17c‐C138‐Cy3‐3′‐ss25‐mer DNA‐antibody bioconjugate probe with a Cy3 fluorophore attached to the 3′‐end of a ss25‐mer ON was prepared (Figure S59). SKBR3 cells were incubated with this bioconjugate or controls, fixed and imaged (see epifluorescence microscopy imaging experiments in the SI). Obtained images demonstrate that Cy3‐labelled DNA ON was localized inside SKBR3 cells only when formulated and delivered as an ON‐antibody conjugate (Figure 5 c) and not detected when cells were incubated with “naked” Cy3‐ON without the antibody vehicle (Figure 5 c). A control experiment in which HER2 receptors were blocked with full‐length anti‐HER2 IgG antibody Trastuzumab resulted in 75 % decrease of the Cy3‐ON signal (Figure S64) which was localized mainly on the cell surface presumably as a result of competition for HER2 receptor binding between 2Rb17c‐C138‐Cy3‐3′‐ss25‐mer and Trastuzumab. Additional evidence was obtained from an imaging experiment in which the same nanobody directly modified with a fluorophore dye was used. Conjugate 2Rb17c‐C138‐C5‐AF488 was prepared by modifying the cysteine residue of the nanobody with AF‐488‐C5‐maleimide under standard conditions (Figure S65). A similar intracellular localization pattern to 2Rb17c‐C138‐Cy3‐3′‐ss25‐mer was observed with 2Rb17c‐C138‐C5‐AF488 conjugate (Figure S66). The results provided by super‐resolution and fluorescence microscopy imaging experiments support the potential of the developed DNA‐antibody bioconjugation platform to be applied for targeted antibody‐mediated delivery of therapeutic ONs.

## Conclusion

We have developed an improved protocol for the bioconjugation of DNA ONs to proteins and antibodies, and an optimized method for the LC‐MS characterization of prepared bioconjugates. By using easy‐to‐prepare BA‐PFP reagent **2**, various types of ss‐ or ds‐amino‐modified DNA ONs can be labelled with a BA moiety. Prepared BA‐modified ONs are then conjugated to proteins and antibodies through cysteine selective thiol‐Michael addition reactions. By conjugating different types of DNA ONs to a wide range of proteins and antibodies, we demonstrate the generality and versatility of our DNA‐protein bioconjugation platform that gives site‐specific and homogenous DNA‐protein conjugates. Importantly, bioconjugates prepared by this method are stable in the presence of human plasma, which makes them useful for various in vitro applications, including imaging, nanofabrication, biosensing or immunological detection methods^[6a]^ and suitable for in vivo bioassays and potentially for therapeutic applications.^[13]^ All these features (confirmed by side‐by‐side comparative experiments) make our BA‐based DNA‐antibody bioconjugation platform superior to the most popular approaches utilizing maleimides. As an example, we have shown that DNA‐antibody probes 2Rb17c‐C138‐ss11‐mer and Thiomab LC‐V205C‐ss11‐mer prepared by our method can be used to image HER2 receptors on the surface of HER2+ SKBR3 breast cancer cells by means of DNA‐PAINT super‐resolution microscopy.[^12^, ^39^] Such studies could open up the door for development of improved diagnostic methods for the quantification of expression levels of HER2 or other receptors in breast and other types of cancer by adapting the method for the quantitative point accumulation in nanoscale topography (qPAINT).^[41]^ In addition, fluorescent DNA‐antibody bioconjugate 2Rb17c‐C138‐Cy3‐3′‐ss25‐mer was used as a probe in epifluorescence microscopy imaging experiments to investigate HER2 receptor‐mediated internalization of this DNA‐antibody construct in SKBR3 cancer cells. Images obtained from these experiments clearly showed intracellular localization of 2Rb17c‐C138‐Cy3‐3′‐ss25‐mer. The results of this work support the potential of the developed bioconjugation platform for therapeutic applications by using antibodies as delivery vehicles for therapeutic ONs. Further studies and developments towards strategies for more efficient cancer therapies by constructing tumor‐targeting nanostructures or various types of NA‐antibody bioconjugates for targeted delivery of therapeutic ONs are underway in our laboratories.

## Conflict of interest

The authors declare no conflict of interest.

## Supporting information

As a service to our authors and readers, this journal provides supporting information supplied by the authors. Such materials are peer reviewed and may be re‐organized for online delivery, but are not copy‐edited or typeset. Technical support issues arising from supporting information (other than missing files) should be addressed to the authors.

Supporting Information
