# Supplementary material for: A Platform for Site‐Specific DNA‐Antibody Bioconjugation by Using Benzoylacrylic‐Labelled Oligonucleotides
Source: Angew Chem Int Ed Engl. 2021 Nov 3;60(49):25905–13. doi: 10.1002/anie.202109713 (PMC9297960; doi:10.1002/anie.202109713)
Supplement: Supplementary file 1 — Supporting Information [file ANIE-60-25905-s001.pdf]

Angewandte Chemie
Eine Zeitschrift der Gesellschaft Deutscher Chemiker

Supporting Information

# A Platform for Site-Specific DNA-Antibody Bioconjugation by Using Benzoylacrylic-Labelled Oligonucleotides

*Juraj Konč, Libby Brown, Daniel R. Whiten, Yukun Zuo, Peter Ravn, Sir David Klenerman, and Gonçalo J. L. Bernardes**

anie_202109713_sm_miscellaneous_information.pdf

**SUPPORTING INFORMATION**

# Table of contents

**General remarks for organic synthesis**……………………………………………………...… 3

**Protein and oligonucleotide LC-MS**………………………………………………………… 4

**Organic synthesis of benzoylacrylic (BA) reagents**….....……………………………...… 7

**Oligonucleotides (ONs) used in this study**...……………………………………………... 10

**General protein methods**…...………………………………………………………………….. 15

**Proteins and antibodies used in this study**...………………..………………………...….. 17

**5'-$NH_2$ DNA ON BA-labelling optimization**……………………………………………... 37

**Preparation of BA-labelled ONs**…………………………………………………………..… 38

**Preparation of maleimide-labelled ONs**…...………………………………………………….. 43

**Hydrolytic stability of maleimide vs BA-labelled ONs**...………………..…………....… 47

**DNA-protein conjugate LC-MS method optimization**…….……………………...…...….. 50

**DNA-protein and DNA-antibody conjugation**…………….……………………………….. 62

**Plasma integrity tests of the 2Rb17c-C138-ss11-mer conjugate**……….....……………….... 86

**Cell culture**...…………………………………………………………………………….... 90

**DNA-PAINT imaging experiments**………………………………………………………… 91

**Epifluorescence microscopy imaging experiments**………………………………………..... 92

**NMR spectra**………………...………………………………………………………………… 97

**References**…..……………....…………………………………………………………….... 100

## General remarks for organic synthesis

All synthetic reaction using anhydrous solvents were performed under $N_2$ atmosphere in dried glassware unless stated otherwise. All the reagents and solvents were purchased from commercial suppliers and used as received. DCM, THF and $Et_2O$ were purified either according to the method of Grubbs and Pangborn[1] or by distillation under an inert atmosphere (DCM, MeOH and MeCN were distilled from calcium hydride. THF and $Et_2O$ were pre-dried over sodium wire then distilled from calcium hydride and lithium aluminium hydride). Petroleum ether (distillate collected between 40–60 ºC), *n*-hexane and EtOAc were distilled on site. Water used for the organic synthetic was deionized and prepared on site. Merck Silica gel 60 was used for the flash column chromatography. Monitoring of reactions was performed using TLC Silica gel 60 F254 plates. Compounds were detected using shortwave (254 nm) UV lamp or by staining with an indicated solution prepared by known procedures. NMR spectra were recorded on Bruker 400-Avance III HD, Avance DPX-400, 400-QNP Cryoprobe (400.1 MHz for $^{1}$H, 100.6 MHz for $^{13}$C and 376.5 MHz for $^{19}$F), in $CDCl_3$ (TMS was used as internal standard), MeOH-*d4* (referenced to the residual solvent signal) acetone-*d6* (referenced to the residual solvent signal) or DMSO-*d6* (referenced to the residual solvent signal). Chemical shifts are given in ppm (δ-scale), coupling constants (*J*) in Hz. High resolution mass spectra were measured on Waters LCT Premier spectrometer using electrospray ionization (ESI). Melting points were measured on a Stuart Scientific SMP3 Melting Point Apparatus and are uncorrected. IR spectra (wavenumbers in $cm^{-1}$) were recorded on a Perkin-Elmer Spectrum One FTIR ATR spectrometer. Peaks were labelled by their relative absorption: w (weak), m (medium), s (strong), br (broad).

## Protein and oligonucleotide LC-MS

LC–MS analysis of small molecule protein modification reactions was performed on a Waters SQ Detector 2 mass spectrometer coupled to an Acquity UPLC system using an Acquity UPLC Protein BEH C4 column 300 Å (1.7 µm, 2.1 × 50 mm) at 40 ºC. Solvents A, milliQ $H_2O$ with 0.1% formic acid and B, 71% LC-MS hypergrade MeCN, 29% milliQ $H_2O$ and 0.075% formic acid were used as the mobile phase at a flow rate of 0.2 mL min$^{-1}$. The gradient was programmed as follows: 72% A to 71.2% B after 12 min, to 100% B after 1 min, then 100% B for 3 min, to 72% A after 0.5 min and after that 72% A for 3.5 min. The electrospray source was operated in the positive mode with a capillary voltage of 3.0 kV and a cone voltage of 30 V. Nitrogen was used as the desolvation gas at a total flow of 800 L·h$^{-1}$ at 400 ºC desolvation temperature.

LC–MS analysis of single-stranded ON modification reactions was performed on a Waters SQ Detector 2 mass spectrometer coupled to an Acquity UPLC system using an Acquity UPLC BEH C18 column (1.7 µm, 2.1 × 50 mm) at 60 ºC. Solvents A, 16.3 mM TEA and 400 mM 1,1,1,3,3,3-hexafluoro-2-propanol (HFIP) in milliQ $H_2O$ and B, 16.3mM TEA and 400mM HFIP in 20% milliQ $H_2O$, 80% LC-MS hypergrade MeCN were used as the mobile phase at a flow rate of 0.2 mL min$^{-1}$. The gradient was programmed as follows: 100% A to 20% B after 5 min, to 100% B after 1 min, then 100% B for 4 min, to 100% A after 0.2 min and after that 100% A for 4.8 min. The electrospray source was operated in the negative mode with a capillary voltage of 3.0 kV and a cone voltage of 25 V. Nitrogen was used as the desolvation gas at a total flow of 800 L·h$^{-1}$ at 400 ºC desolvation temperature.

LC–MS analysis of double-stranded ON modification reactions was performed on a Waters SQ Detector 2 mass spectrometer coupled to an Acquity UPLC system using an Acquity UPLC BEH C18 column (1.7 µm, 2.1 × 50 mm) at 40 ºC. Solvents A, 10 mM hexylammonium acetate (HAA) in milliQ $H_2O$ and B, 10 mM HAA in 20% milliQ $H_2O$, 80% LC-MS hypergrade MeCN were used as the mobile phase at a flow rate of 0.2 mL min$^{-1}$. The gradient was programmed as follows: 100% A to 20% B after 5 min, to 100% B after 1 min, then 100% B for 4 min, to 100% A after 0.2 min and after that 100% A for 4.8 min. The electrospray source was operated in the negative mode

with a capillary voltage of 3.0 kV and a cone voltage of 25 V. Nitrogen was used as the desolvation gas at a total flow of 800 L·h$^{-1}$ at 400 ºC desolvation temperature.

LC–MS analysis of DNA-protein bioconjugation reactions was performed on a Waters SQ Detector 2 mass spectrometer coupled to an Acquity UPLC system using an Acquity UPLC Protein BEH C4 column 300 Å (1.7 µm, 2.1 × 50 mm) at 40 ºC. Solvents A, milliQ $H_2O$ with 10mM $NH_4OAc$ and B, 71% LC-MS hypergrade MeCN, 29% milliQ $H_2O$ and 0.075% formic acid were used as the mobile phase at a flow rate of 0.2 mL min$^{-1}$. The gradient was programmed as follows: 72% A to 71.2% B after 12 min, to 100% B after 1 min, then 100% B for 3 min, to 72% A after 0.5 min and after that 72% A for 3.5 min. The electrospray source was operated in the positive mode with a capillary voltage of 3.0 kV and a cone voltage of 30 V. Nitrogen was used as the desolvation gas at a total flow of 800 L·h$^{-1}$ at 400 ºC desolvation temperature.

Total mass spectra were reconstructed from the ion series using the MaxEnt1 algorithm preinstalled on MassLynx software (v. 4.1 from Waters) according to the manufacturer's instructions. To obtain the ion series described, an approximately two-minute retention time window around the major peak(s) of the total ion chromatogram (TIC) was selected for integration and further analysis.

Concentration of unmodified and modified protein, antibodies and DNA ONs samples was determined using their respective molar extinction coefficient (ε) either provided by the commercial supplier or calculated using Molbiotools DNA Calculator[2] or ExPASy ProtParam tool[3] and the UV absorbance of the solution measured on a Molecular Devices SpectraMax i3x Multi-Mode Microplate Reader (280 nm for proteins and antibodies/260 nm for ONs).

**Note on the LC-MS analysis of oligonucleotides (ONs)**

Extra peaks in the LC-MS spectra (extracted ion series and deconvoluted total mass spectra) from the analysis of unmodified and modified ONs correspond to the adducts of analyzed ONs with LC-MS additives 1,1,1,3,3,3-hexafluoro-2-propanol (+168 Da) or hexylamine (+101 Da).

**Note on the LC-MS analysis of DNA-Gemtuzumab conjugation reactions**

LC-MS analysis of Gemtuzumab HC variants and DNA ONs conjugation reactions was performed using conditions for proteins with formic acid as the LC-MS mobile phase additive. The reason for this was the inefficient sample ionization under the conditions using ammonium acetate as the LC-MS mobile phase additive caused by the glycosylation of antibody HCs.

## Organic synthesis of benzoylacrylic acid (BA) reagents

### Pentafluorophenyl (*E*)-4-oxo-4-phenylbut-2-enoate (2)

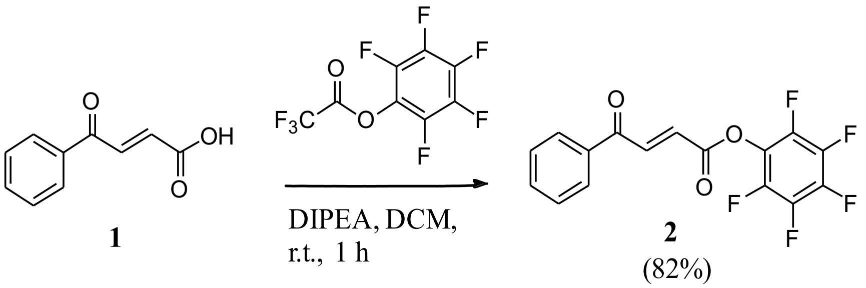


3-Benzoylacrylic acid (**1**) (5.3 g, 30.1 mmol) was dissolved in anhydrous DCM (60 mL) and DIPEA (10.5 mL, 60.3 mmol) was added followed by dropwise addition of pentafluorophenyl trifluoroacetate (6.2 mL, 36.1 mmol), under $N_2$ atmosphere. The reaction mixture was stirred at r.t. for 1 h. Solvents were removed under reduced pressure and the crude product was purified by flash column chromatography (silica gel, 50% DCM in PE) yielding in the title compound **2** (8.48 g, 24.8 mmol, 82%) as a yellow solid.

$R_f$ 0.83 (10% MeOH/DCM, UV 254 nm).

Mp 81.7–82.2 ºC.

$^1$H NMR (400.1 MHz, $CDCl_3$) δ 8.19 (d, *J* = 15.6 Hz, 1H, *H*3), 8.07–8.06 (m, 2H, *H*2''), 7.72–7.67 (m, 1H, *H*4''), 7.60–7.56 (m, 2H, *H*3''), 7.14 (d, *J* = 15.6 Hz, 1H, *H*2).

$^{13}$C NMR (100.6 MHz, $CDCl_3$) δ 188.5, 161.6, 140.4, 136.3, 134.5, 129.2, 129.1, 128.7, 125.1.

$^{19}$F NMR (376.5 MHz, $CDCl_3$) δ -153.2 (d, *J* = 18.0 Hz, 2F, *o*-F), -158.1 (t, *J* = 21.6 Hz, 1F, *p*-F), -162.8 (dd, *J* = 18.2, 21.5 Hz, 2F, *m*-F).

IR (thin film): ν (cm$^{-1}$) = 1767m, 1670m, 1514s, 1449w, 1290m, 1118m, 993s, 717m, 684m.

HRMS (ESI$^+$): *m/z* calcd for $C_{16}H_7F_5O_3Na$ $[M+Na]^+$ 365.0207, found 365.1706.

**(*E*)-*N*-ethyl-4-oxo-4-phenylbut-2-enamide (3)**

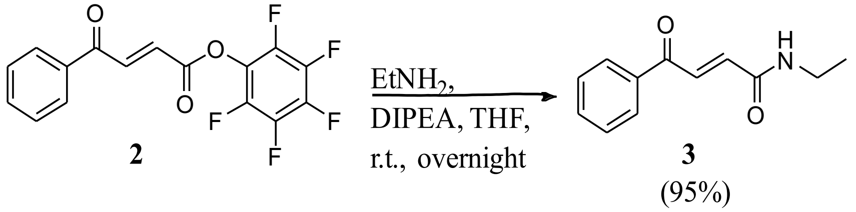


To a solution of PFP ester **2** (80 mg, 0.23 mmol) in anhydrous THF (1 mL), under $N_2$ atmosphere, $EtNH_2$ (2 M in THF, 230 μL, 0.46 mmol) and DIPEA (80 μL, 0.46 mmol) were added. The reaction mixture was stirred at r.t., overnight. Solvents were removed under reduced pressure and the crude product was purified by flash column chromatography (silica gel, DCM → 2% MeOH in DCM) to afford the title compound **3** (45 mg, 0.22 mmol, 95%) as a pale-yellow solid.

$R_f$ 0.69 (10% MeOH/DCM, UV 254 nm, ninhydrin).

$^1$H NMR (400.1 MHz, $CDCl_3$) δ 8.02–8.04 (m, 2H, *H*2''), 7.98 (d, *J* = 14.8 Hz, 1H, *H*3), 7.64–7.60 (m, 1H, *H*4''), 7.53–7.49 (m, 2H, *H*3''), 6.97 (d, *J* = 14.8 Hz, 1H, *H*2), 6.07 (s, 1H, N*H*), 3.50–3.43 (m, 2H, *H*1'), 1.23 (t, *J* = Hz, 3H, *H*2'). $^1$H NMR data are in accordance with the previously reported literature data.[4]

**(*E*)-*N*-(2-(2-(4-Oxo-4-phenylbut-2-enamido)ethoxy)ethyl)-5-(2-oxohexahydro-1*H*-thieno[3,4-*d*]imidazol-4-yl)pentanamide (4)**

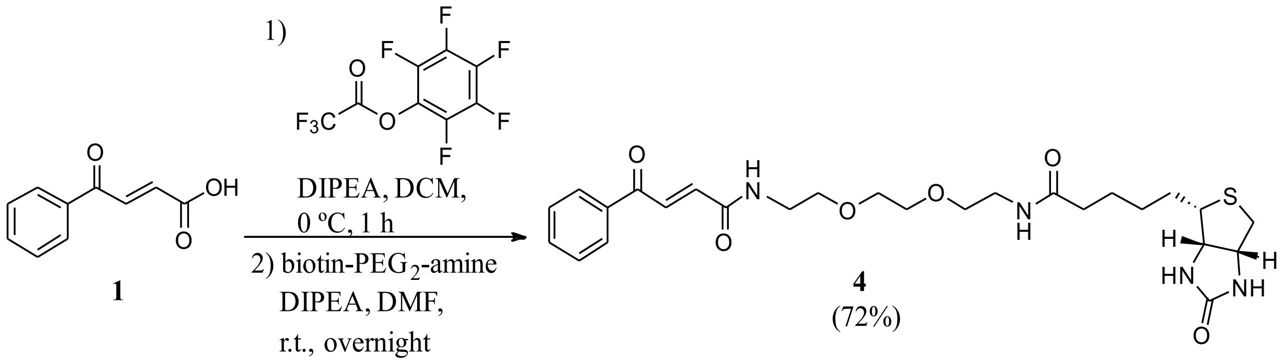


To a solution of 3-benzoylacrylic acid (**1**) (6 mg, 0.034 mmol) in anhydrous DCM (210 μL), under $N_2$ atmosphere, DIPEA (11.9 μL, 0.068 mmol) was added and the reaction mixture was cooled with ice bath. Then, pentafluorophenyl trifluoroacetate (6.2 μL, 0.036 mmol) was added and the reaction mixture was stirred with cooling for 1 h, after which the solvents were removed under reduced pressure. The residue was dissolved in anhydrous DMF (0.5 mL) and added to a solution of biotin-PEG$_2$-amine (13 mg, 0.034 mmol) in anhydrous DMF (1 mL). DIPEA (9 μL, 0.052

mmol) was added and the resulting mixture was stirred at r.t., overnight. Solvents were then evaporated under reduced pressure and the residue was co-evaporated with toluene (3×2 mL). The crude product was purified by flash chromatography (silica gel, DCM → 10% MeOH in DCM) to give the product **4** (13 mg, 0.024 mmol, 75%) as an off-white solid.

$R_f$ 0.83 (10% MeOH/DCM, UV 254 nm).

Mp 99.1–99.8 ºC.

$^1$H NMR (400.1 MHz, MeOH-*d4*) δ 8.06–8.03 (m, 2H, *H*2''), 7.90 (d, *J* = 15.2 Hz, 1H, *H*3), 7.69–7.65 (m, 1H, *H*4''), 7.93–7.54 (m, 2H, *H*3''), 7.14 (d, *J* = 15.2 Hz, 1H, *H*2), 4.59–4.56 (m, 1H, *H*14'), 4.30–4.27 (m, 1H, *H*15'), 3.67–3.63 (m, 6H, *H*3' + *H*4' + *H*5'/*H*2'), 3.57–3.51 (m, 4H, *H*1'/*H*6' + *H*5'/*H*2'), 3.37 (t, *J* = 5.5 Hz, 2H, *H*1'/*H*6'), 3.21–3.16 (m, 1H, *H*12'), 2.91 (dd, *J* = 12.7, 4.9 Hz, 1H, *H*13'), 2.69 (d, *J* = 12.6 Hz, 1H, *H*13'), 2.21 (t, *J* = 7.4 Hz, 2H, *H*8'), 1.75–1.55 (m, 4H, *H*9' + *H*11'), 1.43 (q, *J* = 7.6 Hz 2H, *H*10').

$^{13}$C NMR (100.6 MHz, $CDCl_3$) δ 191.5, 176.2, 166.8, 138.2, 136.5, 135.0, 134.1, 130.1, 129.9, 126.4, 71.4, 71.3, 70.6, 70.3, 63.4, 61.6, 57.0, 41.0, 40.8, 40.3, 36.8, 29.7, 29.5, 26.8.

IR (thin film): ν ($cm^{-1}$) = 3278br, 2925br, 1768w, 1688m, 1645m, 1520s, 1449w, 1328w, 1286m, 1118m, 999m, 719m.

HRMS ($ESI^+$): *m/z* calcd for $C_{26}H_{37}N_4O_6S$ $[M+H]^+$ 533.2428, found 533.2392.

## Oligonucleotides (ONs) used in this study

All ONs used in this study were obtained from Integrated DNA Technologies IDT.

### 5'-NH$_2$-ss11-mer

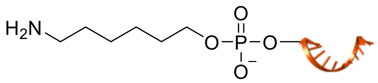


Oligonucleotide sequence:

5'-NH$_2$-C6-TTA TAC ATC TA-3'

Molecular weight: 3469 Da; observed: 3471 Da.

$\varepsilon_{260}$: 110600 L · mol$^{-1}$ · cm$^{-1}$

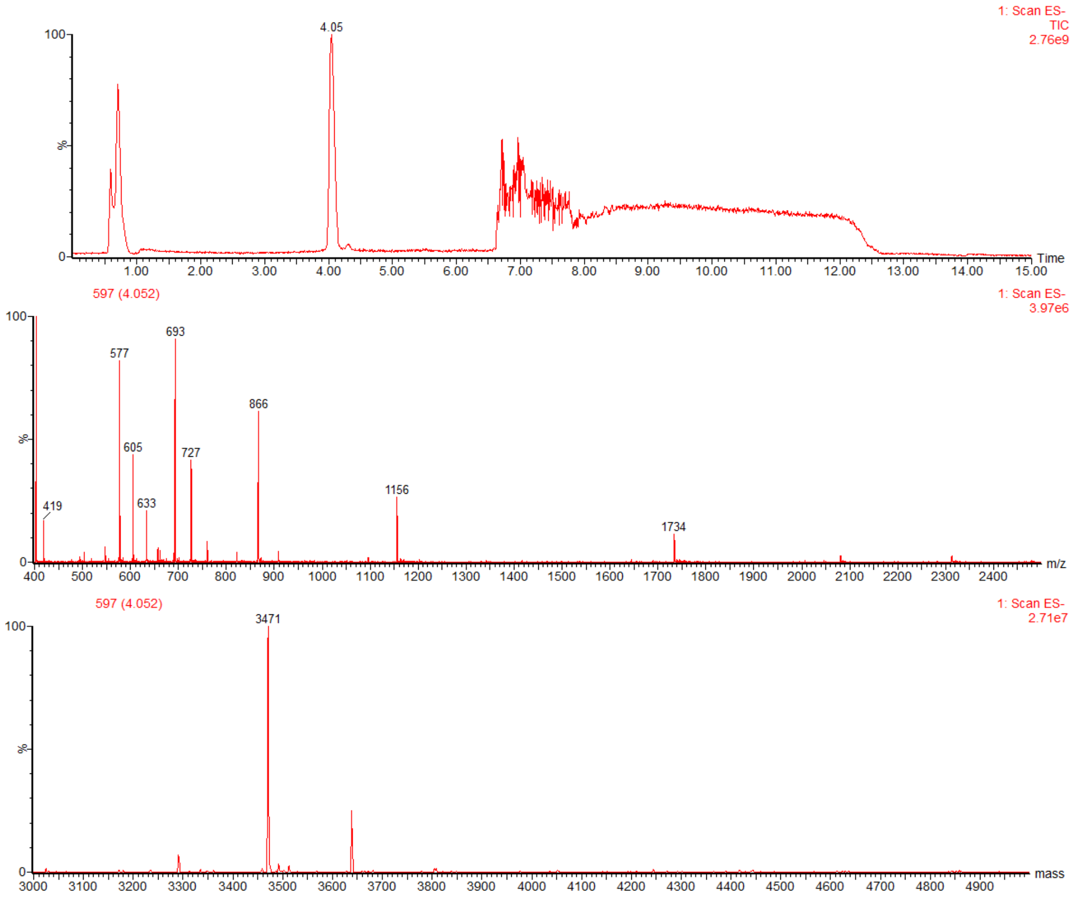


**Figure S1.** LC-MS analysis of a sample of the **5'-NH$_2$-ss11-mer** ON.

**5'- NH$_2$-ss25-mer**

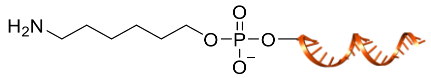


Oligonucleotide sequence:

5'-NH$_2$-C6-AAA ATC GGC GAC CTC CGT AGC AGC G-3'

Molecular weight: 7840 Da; observed: 7843 Da.

$\varepsilon_{260}$: 242500 L · mol$^{-1}$ · cm$^{-1}$

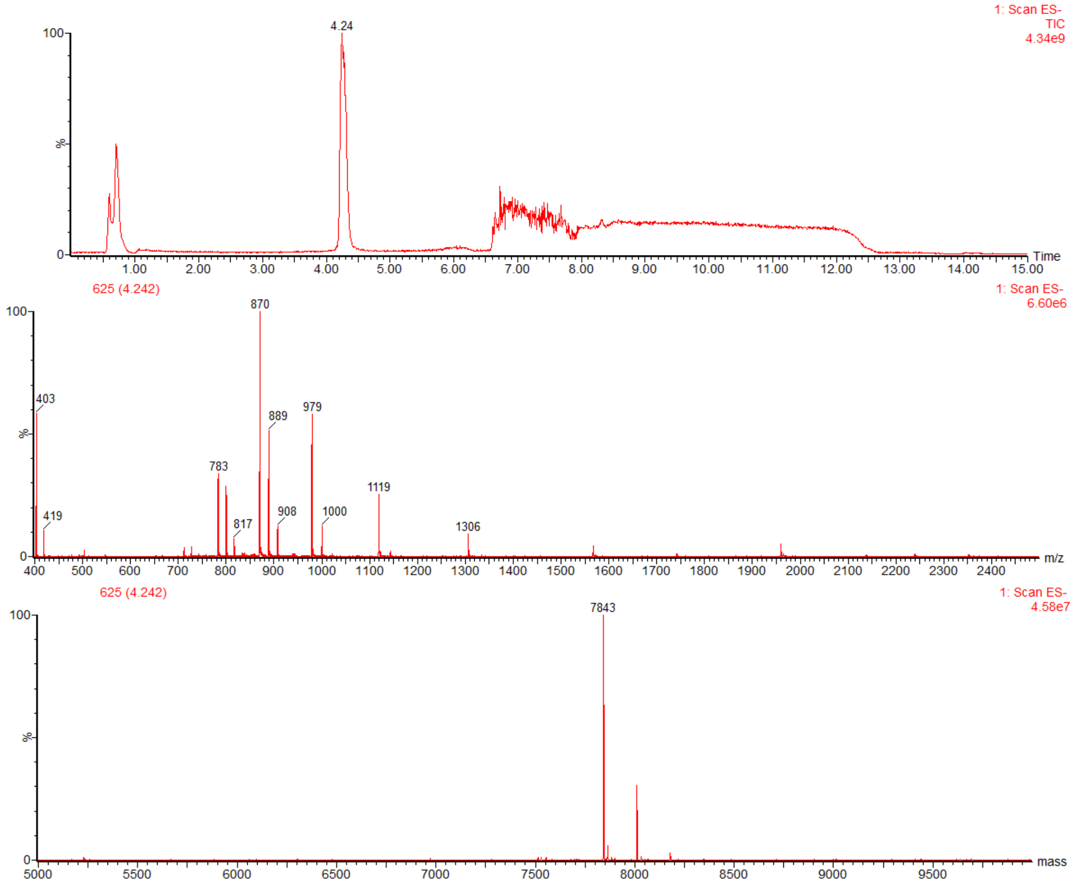


**Figure S2.** LC-MS analysis of a sample of the **5'-NH$_2$-ss25-mer** ON.

**5'- NH$_2$-ss50-mer**

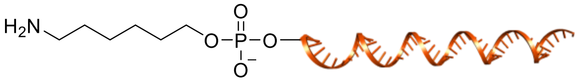

Oligonucleotide sequence:

5'-NH$_2$-C6-ATA TAA CTC GTA AGC CCT AGC AAG CCG CTG CTA CGG AGG TCG CCG ATT TT-3'

Molecular weight: 15525 Da; observed: 15532 Da.

$\varepsilon_{260}$: 474700 L · mol$^{-1}$ · cm$^{-1}$

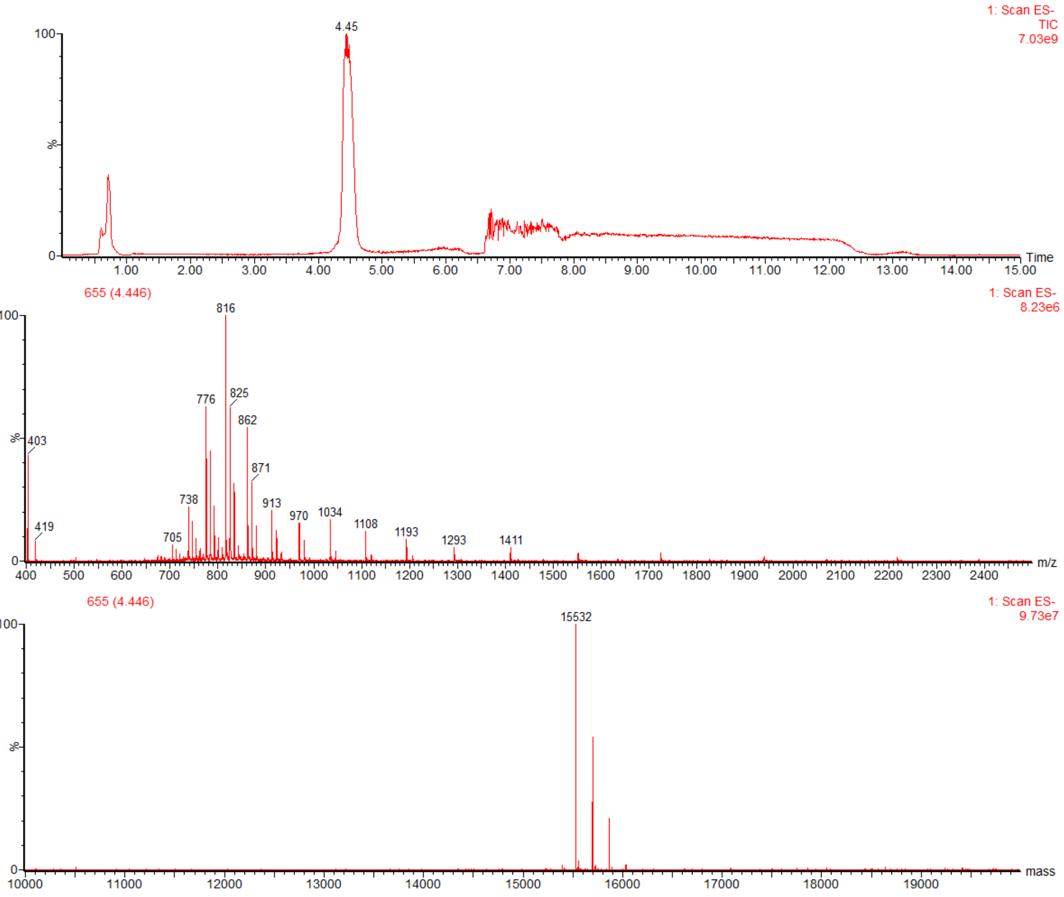


**Figure S3.** LC-MS analysis of a sample of the **5'-NH$_2$-ss50-mer** ON.

**5'- NH$_2$-ds19-mer**

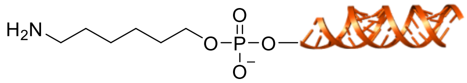


Oligonucleotide sequence (sense):

5'-NH$_2$-C6-TTG TCC CGG TGC CGG ACG C-3'

Molecular weight: 5976 Da; observed: 5976 Da.

$\varepsilon_{260}$: 167200 L · mol$^{-1}$ · cm$^{-1}$

Oligonucleotide sequence (antisense):

5'-GCG TCC GGC ACC GGG ACA A-3'

Molecular weight: 5824 Da; observed: 5824 Da.

$\varepsilon_{260}$: 181100 L · mol$^{-1}$ · cm$^{-1}$

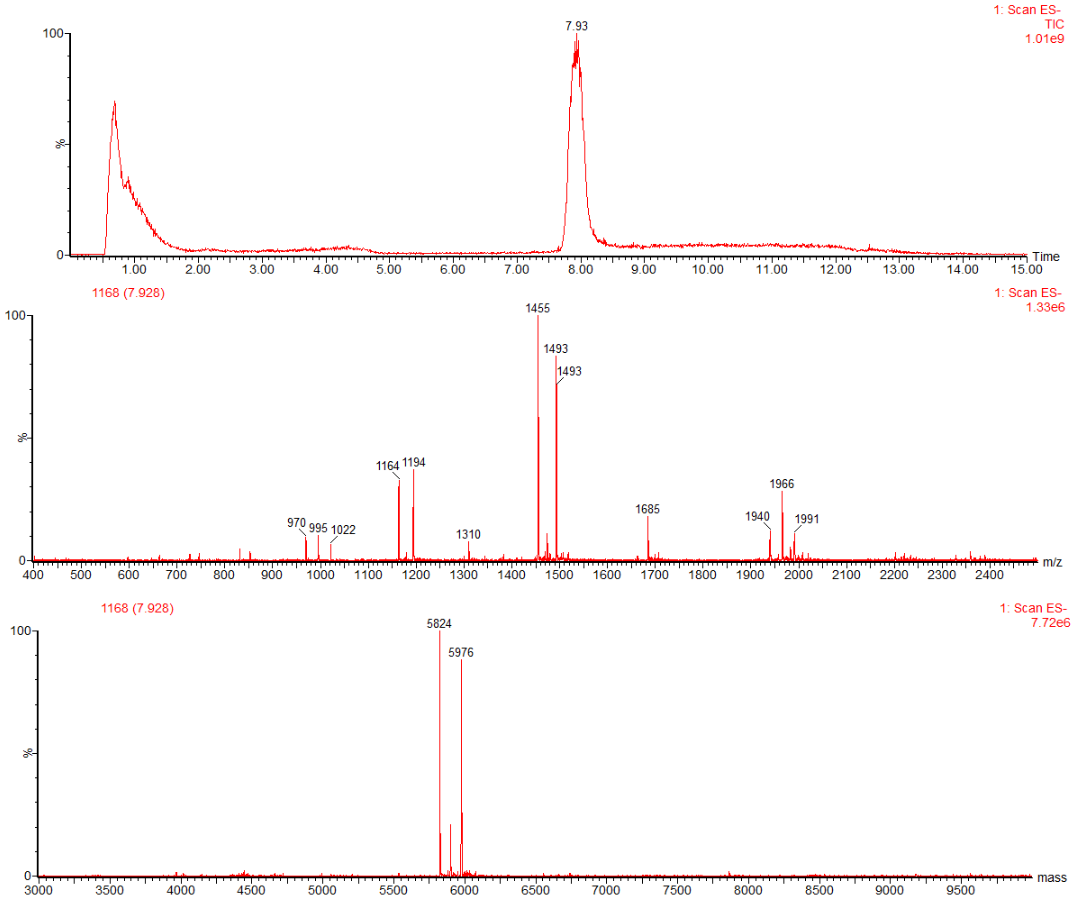


**Figure S4.** LC-MS analysis of a sample of the **5'-NH$_2$-ds19-mer** ON.

**5'- NH$_2$-Cy3-3'-ss25-mer**

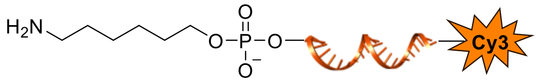


Oligonucleotide sequence:

5'-NH$_2$-C6-AAA ATC GGC GAC CTC CGT AGC AGC G-Cy3-3'

Molecular weight: 8485 Da; observed: 8486 Da.

$\varepsilon_{260}$: 247400 L · mol$^{-1}$ · cm$^{-1}$

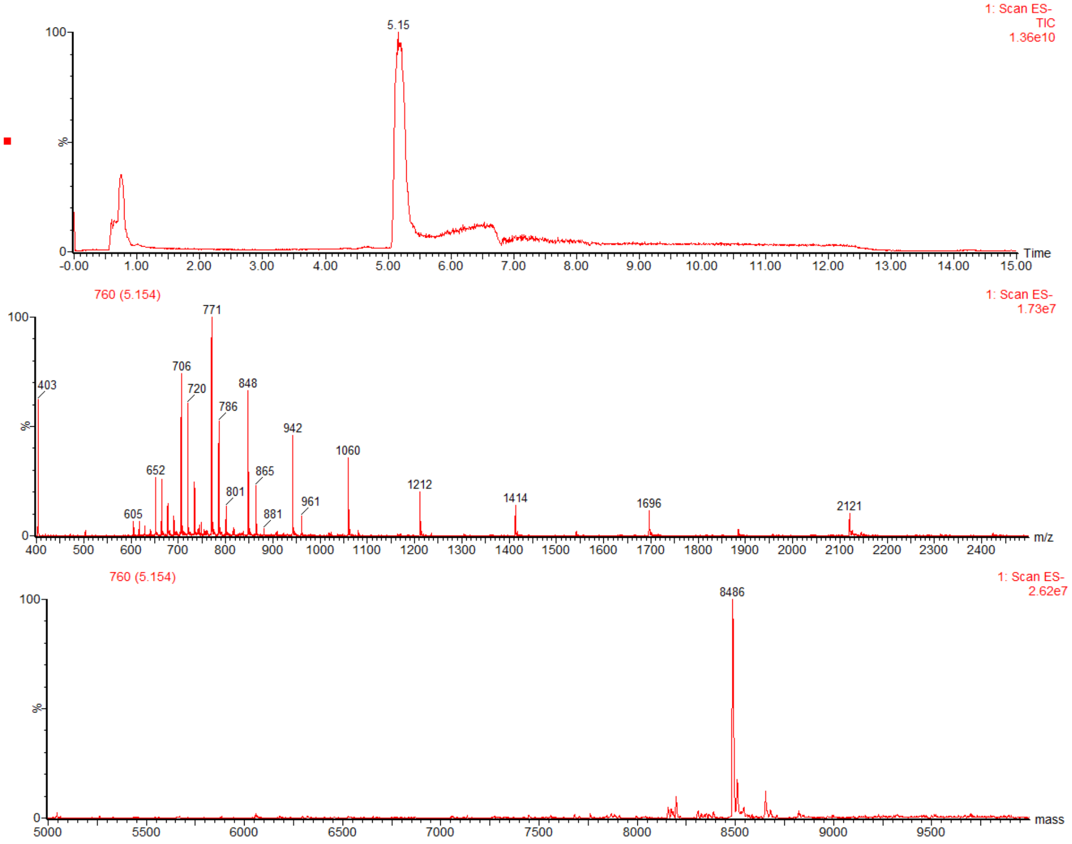


**Figure S5.** LC-MS analysis of a sample of the **5'-NH$_2$-Cy3-3'-ss25-mer** ON.

## General protein methods

**Reduction of cysteine disulfide dimer of proteins and antibodies**

Prior to bioconjugation reactions, Cys proteins and antibodies prone to dimerization were reduced by treatment with TCEP·HCl (20 equiv./protein, in milliQ $H_2O$, added in 0.5–1 µL volume) in 50 mM $NaP_i$ pH 8.0, the reaction mixture was vortexed and shaken at 37 ºC or 25 ºC (for proteins prone to degradation at 37 ºC) for 30 min. Proteins were purified from small molecules using Zeba™ Micro Spin Desalting Column 7K MWCO and used directly in the next protein modification step.

**Pre-LC-MS full-length IgG antibody sample reduction**

Prior to LC-MS analysis, IgG antibodies were reduced to light (LC) and heavy chains (HC) by treatment with TCEP·HCl (20 equiv./antibody, in milliQ $H_2O$, added in 0.5–1 µL volume) in 50 mM $NaP_i$ pH 8.0, the reaction mixture was vortexed and shaken at 37 ºC for 30 min. Aliquots of the reaction mixture were then analyzed by LC-MS.

**Decapping of engineered cysteine residues on full-length IgG antibodies**

Prior to bioconjugation reaction, IgG antibodies with capped engineered cysteine residues were reduced by treatment with TCEP·HCl (20 equiv./antibody, in milliQ $H_2O$, added in 0.5–1 µL volume) in 50 mM $NaP_i$ pH 8.0, the reaction mixture was vortexed and shaken at 37 ºC for 30 min. antibodies were purified from small molecules using Zeba™ Micro Spin Desalting Column 7K MWCO and native antibody disulfide bonds were then re-oxidised by treatment with L-dehydroascorbic acid (20 equiv./antibody, in DMF, 10% DMF final concentration) in 50 mM $NaP_i$ pH 8.0, the reaction mixture was vortexed and shaken at 37 ºC for 4 h. Small molecules were removed using Zeba™ Micro Spin Desalting Column 7K MWCO and antibody with free engineered cysteine residues were used directly in the next protein modification step or stored at 4 ºC.

Number of free cysteine residues on antibodies was confirmed by the reaction with Ellman's reagent (5,5'-dithio-bis-(2-nitrobenzoic acid), DTNB). DTNB (100 equiv./antibody, in DMF, 10% DMF final concentration) was added to the solution of antibody in 50 mM $NaP_i$ pH 8.0, the reaction mixture was vortexed and shaken at 37 ºC for 1 h. Unreacted DTNB was removed using Zeba™

Micro Spin Desalting Column 7K MWCO, samples were reduced by treatment with TCEP·HCl (20 equiv./antibody, in milliQ $H_2O$, added in 0.5–1 μL volume) in 50 mM $NaP_i$ pH 8.0 at 37 ºC for 30 min and the absorbance of the sample was measured at 412 nm ($\varepsilon_{412}$ = 14150 L · $mol^{-1}$ · $cm^{-1}$ for the 2-nitro-5-thiobenzoate dianion) and 280 nm (using $\varepsilon_{280}$ for the corresponding antibody) and concentration calculated using Beer-Lambert law.

## Proteins and antibodies used in this study

**Ubiquitin K63C** (expressed in the Bernardes laboratory)[5]

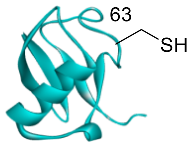


Amino acid sequence:

SAQIFVKTLTGKTITLEVEPSDTIENVKAKIQDKEGIPPDQQRLIFAGKQLEDGRTLSDYNI
Q**C**ESTLHLVLRLRGG

Molecular weight: 8567 Da; observed: 8569 Da.

$\varepsilon_{280}$: 1490 L · mol$^{-1}$ · cm$^{-1}$

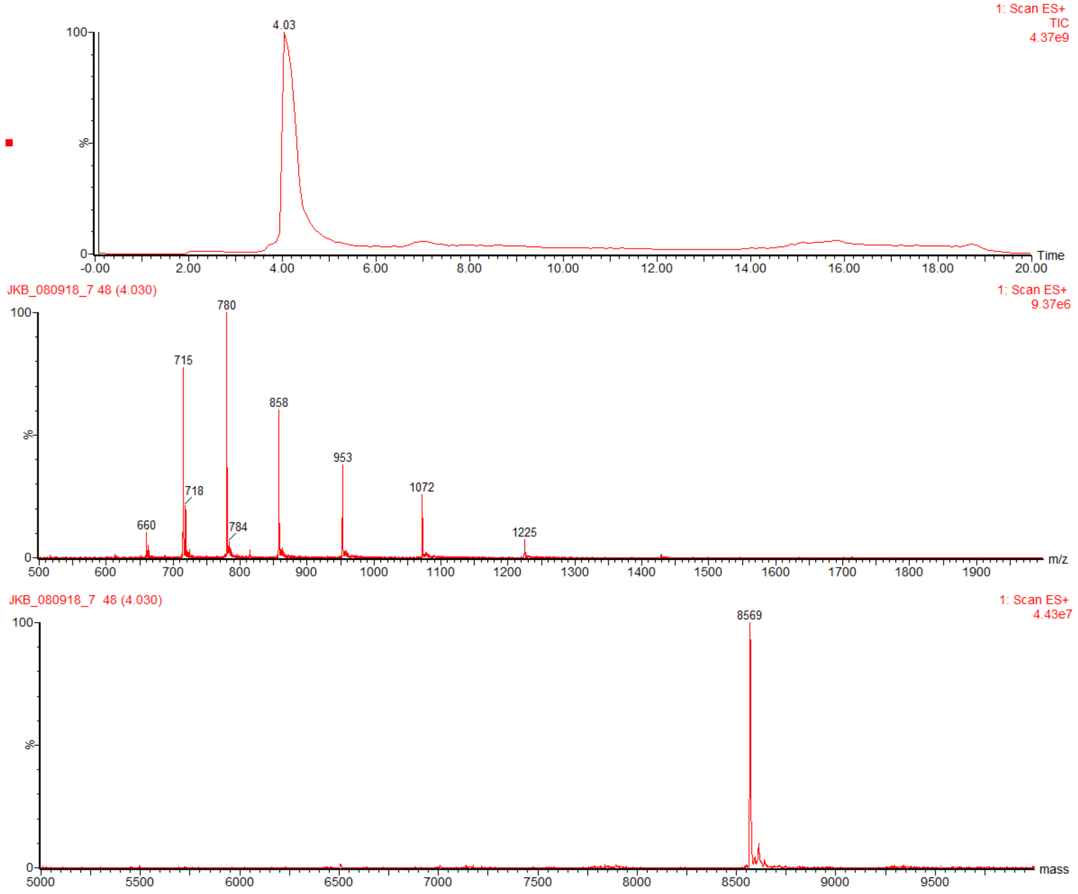


**Figure S6.** LC-MS analysis of a sample of the **Ubiquitin K63C** protein.

**C2Am-C95** (provided by Dr. André Neves and Prof. Kevin Brindle)[6]

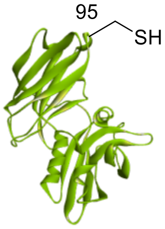


Amino acid sequence:

GSPGISGGGGGILDSMVEKLGKLQYSLDYDFQNNQLLVGIIQAAELPALDMGGTSDPYV
KVFLLPDKKKKFETKVHRKTLNPVFNEQFTFKVPY<u>**C**</u>ELGGKTLVMAVYDFDRFSKHDII
GEFKVPMNTVDFGHVTEEWRDLQSAEK

Molecular weight: 16222 Da; observed: 16222 Da.

$\varepsilon_{280}$: 12090 L · mol$^{-1}$ · cm$^{-1}$

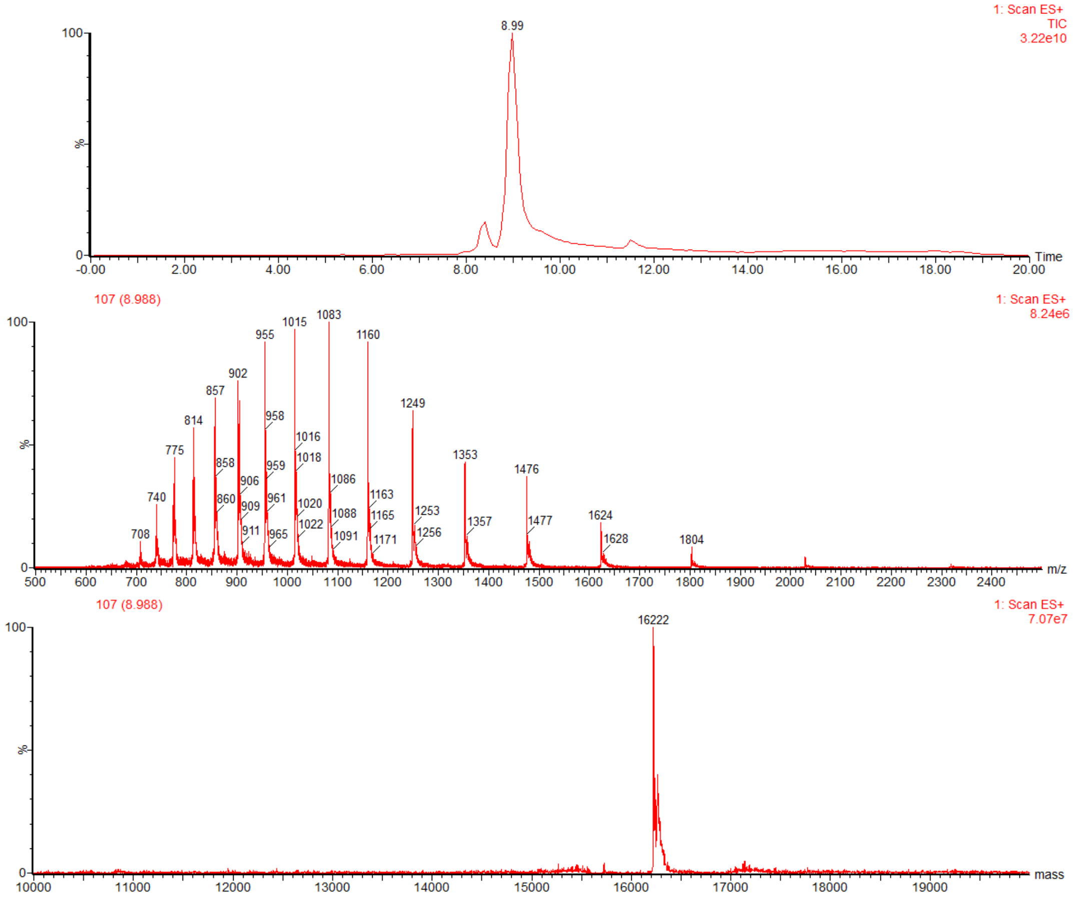


**Figure S7.** LC-MS analysis of a sample of the **C2Am-C95** protein.

**Annexin V-C315** (expressed in the Bernardes laboratory)[7]

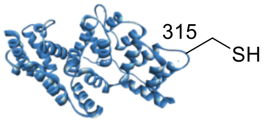


Amino acid sequence:

AQVLRGTVTDFPGFDERADAETLRKAMKGLGTDEESILTLLTSRSNAQRQEISAAFKTLF
GRDLLDDLKSELTGKFEKLIVALMKPSRLYDAYELKHALKGAGTNEKVLTEIIASRTPEE
LRAIKQVYEEEYGSSLEDDVVGDTSGYYQRMLVVLLQANRDPDAGIDEAQVEQDAQAL
FQAGELKWGTDEEKFITIFGTRSVSHLRKVFDKYMTISGFQIEETIDRETSGNLEQLLLAV
VKSIRSIPAYLAETLYYAMKGAGTDDHTLIRVMVSRSEIDLFNIRKEFRKNFATSLYSMIK
GDTSGDYKKALLLL**C**GEDD

Molecular weight: 35805 Da; observed: 35811 Da.

$\varepsilon_{280}$: 23380 L · mol$^{-1}$ · cm$^{-1}$

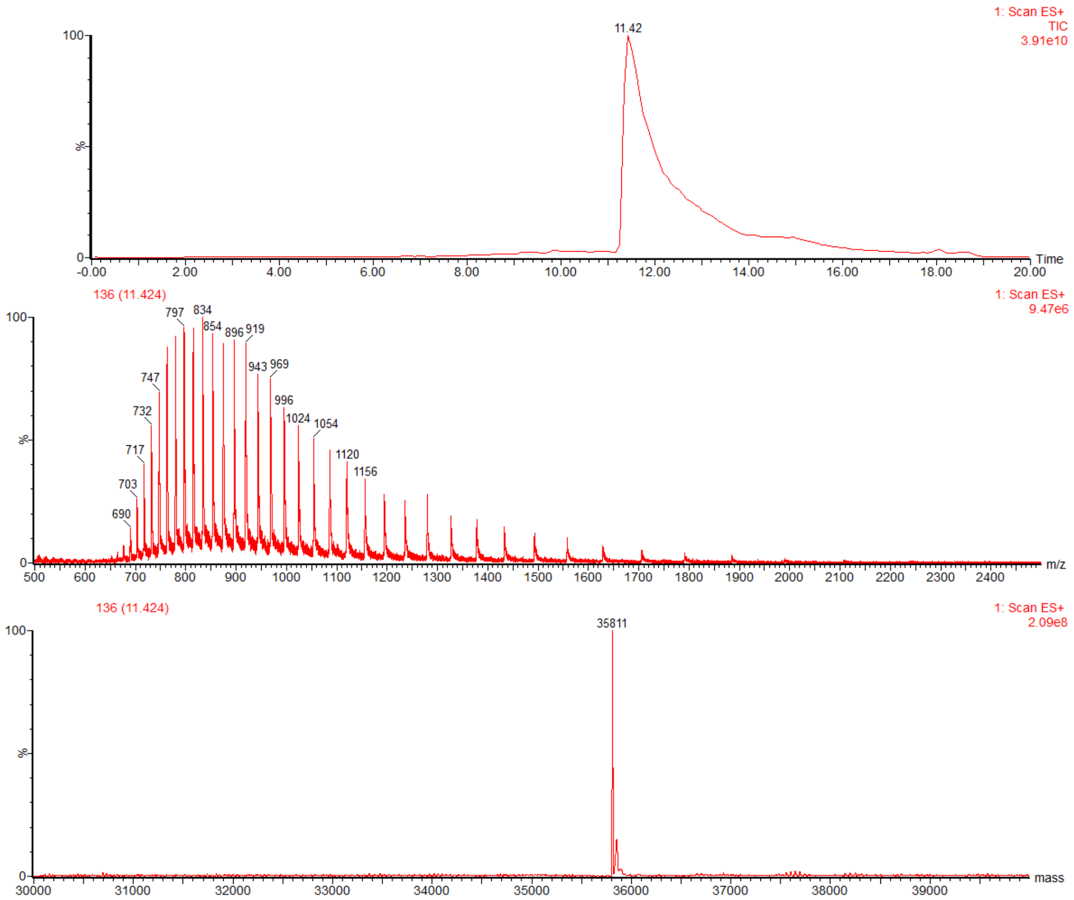


**Figure S8.** LC-MS analysis of a sample of the **Annexin V-C315** protein.

**HSA-C34** (provided by Albumedix)

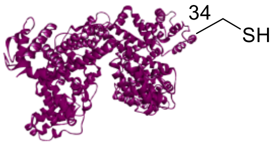


Amino acid sequence:

DAHKSEVAHRFKDLGEENFKALVLIAFAQYLQQ**C**PFEDHVKLVNEVTEFAKTCVADES
AENCDKSLHTLFGDKLCTVATLRETYGEMADCCAKQEPERNECFLQHKDDNPNLPRLV
RPEVDVMCTAFHDNEETFLKKYLYEIARRHPYFYAPELLFFAKRYKAAFTECCQAADK
AACLLPKLDELRDEGKASSAKQRLKCASLQKFGERAFKAWAVARLSQRFPKAEFAEVS
KLVTDLTKVHTECCHGDLLECADDRADLAKYICENQDSISSKLKECCEKPLLEKSHCIAE
VENDEMPADLPSLAADFVESKDVCKNYAEAKDVFLGMFLYEYARRHPDYSVVLLLRL
AKTYETTLEKCCAAADPHECYAKVFDEFKPLVEEPQNLIKQNCELFEQLGEYKFQNALL
VRYTKKVPQVSTPTLVEVSRNLGKVGSKCCKHPEAKRMPCAEDYLSVVLNQLCVLHEK
TPVSDRVTKCCTESLVNRRPCFSALEVDETYVPKEFNAETFTFHADICTLSEKERQIKKQ
TALVELVKHKPKATKEQLKAVMDDFAAFVEKCCKADDKETCFAEEGKKLVAASQAAL
GL

Molecular Weight (with 17 internal disulfides): 66438 Da; observed: 66455 Da.

$\varepsilon_{280}$ = 34445 L · mol$^{-1}$ · cm$^{-1}$

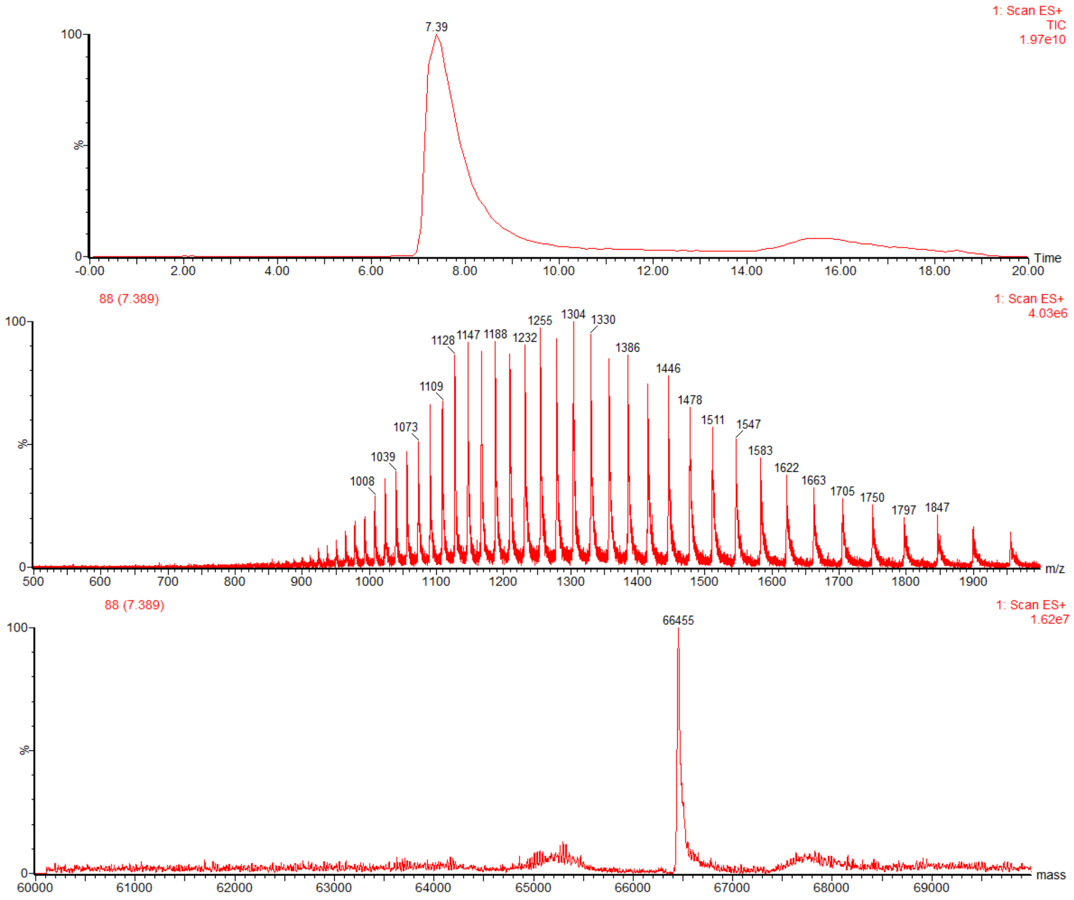


**Figure S9.** LC-MS analysis of a sample of the **HSA-C34** protein.

**594Nup98 G85C** (provided by Dr. Tino Pleiner and Prof. Dr. Dirk Görlich)[8]

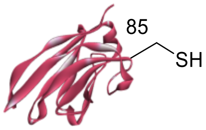


Amino acid sequence:

GSQVQLVESGGGPVEAGGSLRLSCAASGRSFSNSVMAWFRQAPGKEREFLSVLNWSSG
RTSIADSVKGRFTMSRDPAKITVYLQMN<u>**C**</u>LKPEDTAVYYCAASNRGSLYTLDNQNRYE
DWGQGTQVTVSS

Molecular weight: 13886 Da; observed: 13883 Da.

$\varepsilon_{280} = 24325\ \mathrm{L \cdot mol^{-1} \cdot cm^{-1}}$

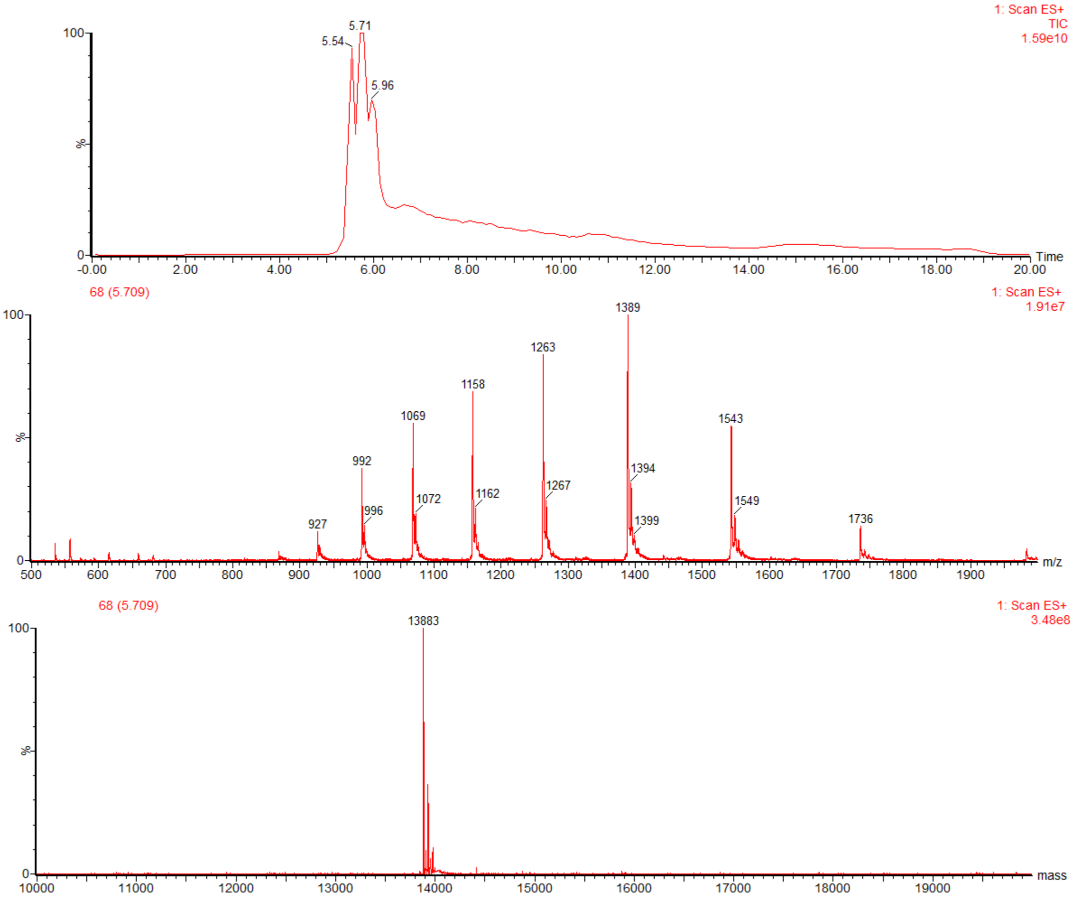


**Figure S10.** LC-MS analysis of a sample of the **594Nup98 G85C** antibody.

**576Nup98 A75C** (provided by Dr. Tino Pleiner and Prof. Dirk Görlich)[8]

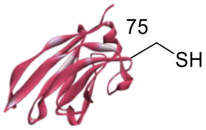


Amino acid sequence:

GSQVQLVESGGGPVEAGGSLRLSCAASGRSFSNSVMAWFRQAPGKEREFLSVLNWSSG
RTSIADSVKGRFTMCRDPAKITVYLQMNGLKPEDTAVYYCAASNRGSLYTLDNQNRYE
DWGQGTQVTVSS

Molecular weight: 13856 Da; observed: 13852 Da.

$\varepsilon_{280}$ = 24325 L · mol$^{-1}$ · cm$^{-1}$

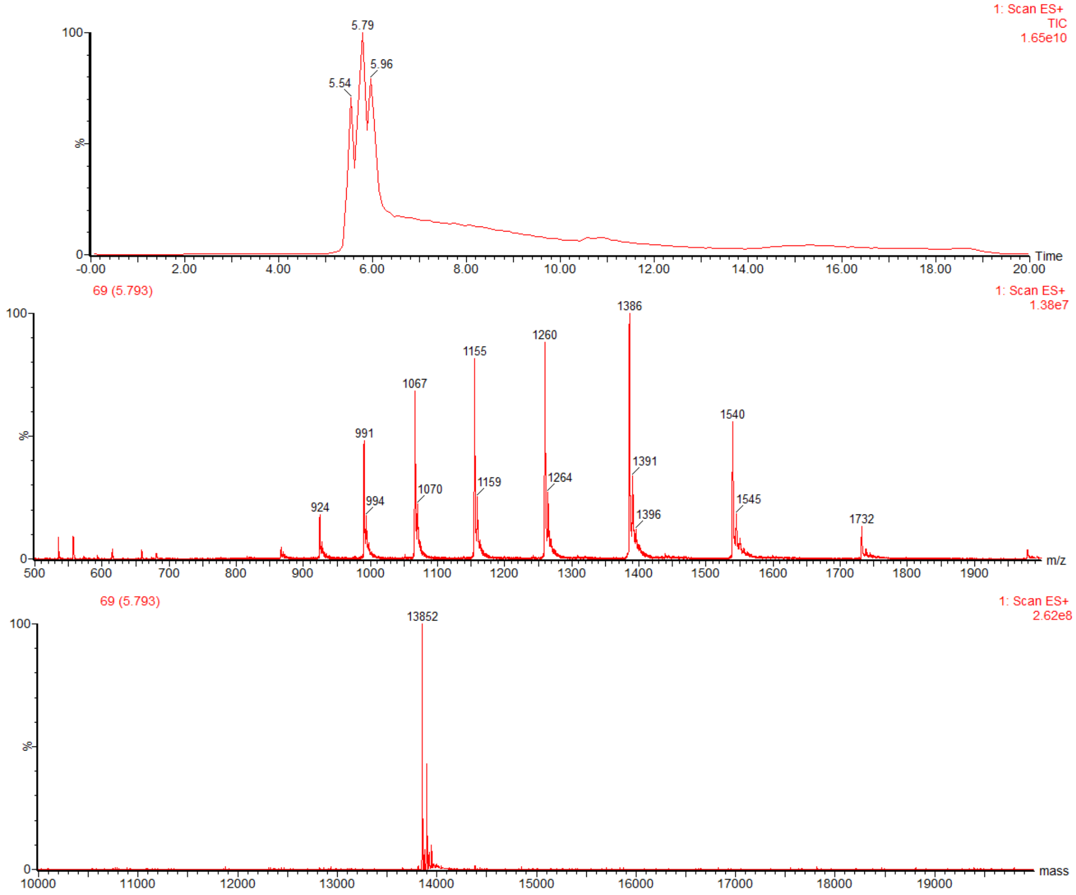


**Figure S11.** LC-MS analysis of a sample of the **576Nup98 A75C** antibody.

**427Nup93 C4** (provided by Dr. Tino Pleiner and Prof. Dirk Görlich)[8]

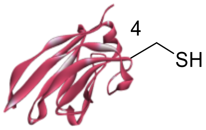


Amino acid sequence:

AGT**<u>C</u>**GSDVQLVESGGGSVQAGGSLRLSCTASGGTFSSYYMGWFRQAPGKEREFVAAIN
KSGDSTYYADSVKGRFTMSRDNAKNTVYLQMNSLNADDTAVYYCAARPTGTYDYWG
QGTQVTVSSGR

Molecular weight: 13296 Da; observed: 13295 Da.

$\varepsilon_{280} = 24785\ \mathrm{L \cdot mol^{-1} \cdot cm^{-1}}$

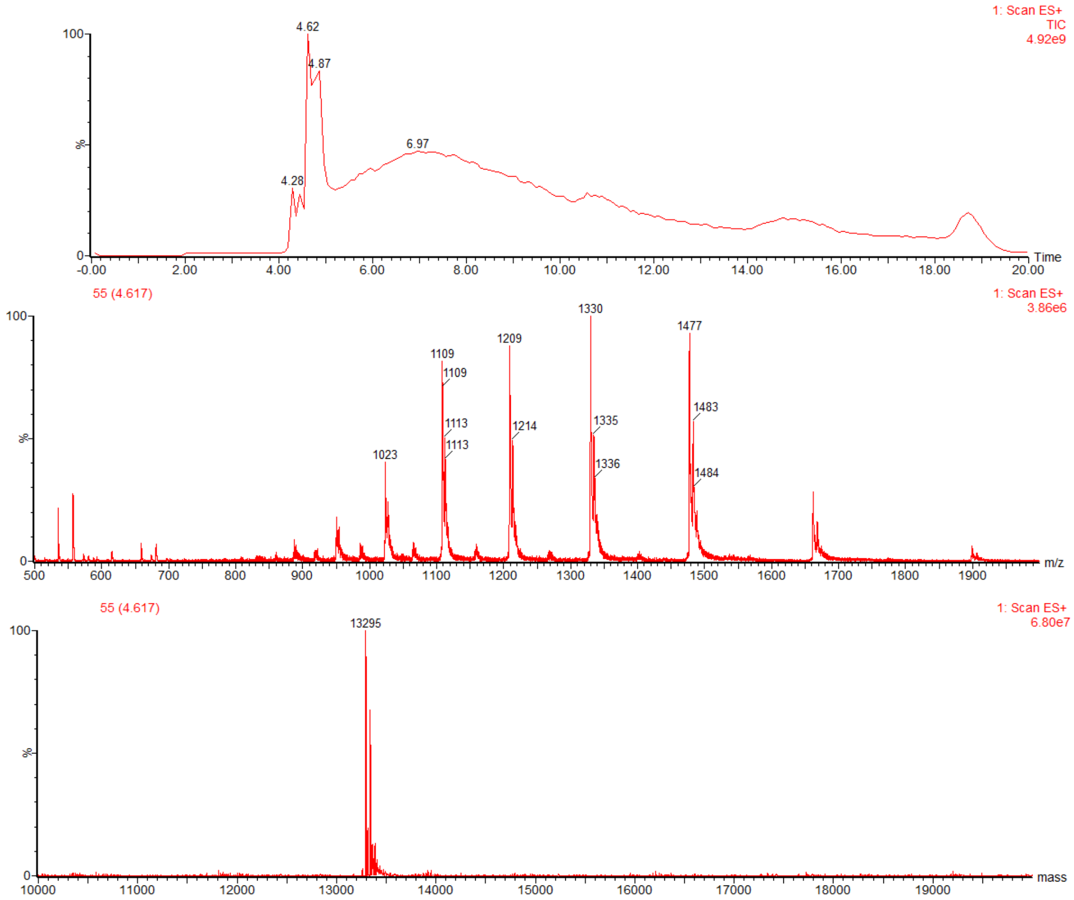


**Figure S12.** LC-MS analysis of a sample of the **427Nup93 C4** antibody.

**443Nup98 C4** (provided by Dr. Tino Pleiner and Prof. Dirk Görlich)[8]

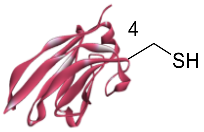


Amino acid sequence:

AGT**C**GSQVQLVESGGGPVEAGGSLRLSCAASGRSFSNSVMAWFRQAPGKEREFLSVLN
WSSGRTSIADSVKGRFTMSRDPAKITVYLQMNGLKPEDTAVYYCAASNRGSLYTLDNQ
NRYEDWGQGTQVTVSSGR

Molecular weight: 14386 Da; observed: 14384 Da.

$\varepsilon_{280} = 24325\ L \cdot mol^{-1} \cdot cm^{-1}$

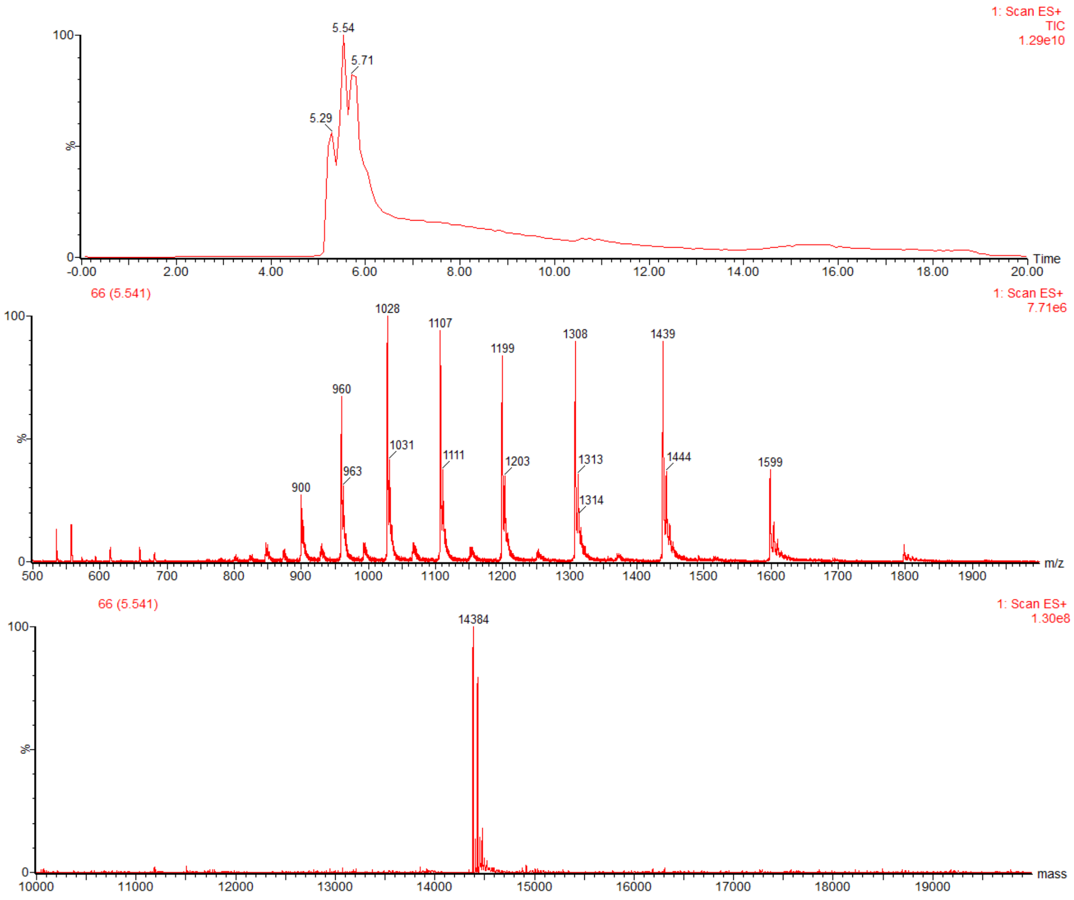


**Figure S13.** LC-MS analysis of a sample of the **443Nup98 C4** antibody.

**2Rb17c-C138** (provided by Dr. Sam Massa and Prof. Nick Devoogdt)[9]

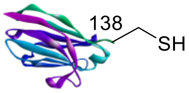


Amino acid sequence:

EVQLQESGGGLVQPGGSLRLSCAASGFIFSNDAMTWVRQAPGKGLEWVSSINWSGTHT

NYADSVKGRFTISRDNAKRTLYLQMNSLKDEDTALYYCVTGYGVTKTPTGQGTQVTVS

SHHHHHHSPSTPPTPSPSTPP**C**

Molecular weight: 14861 Da; observed: 14860 Da.

$\varepsilon_{280} = 24075\ \mathrm{L \cdot mol^{-1} \cdot cm^{-1}}$

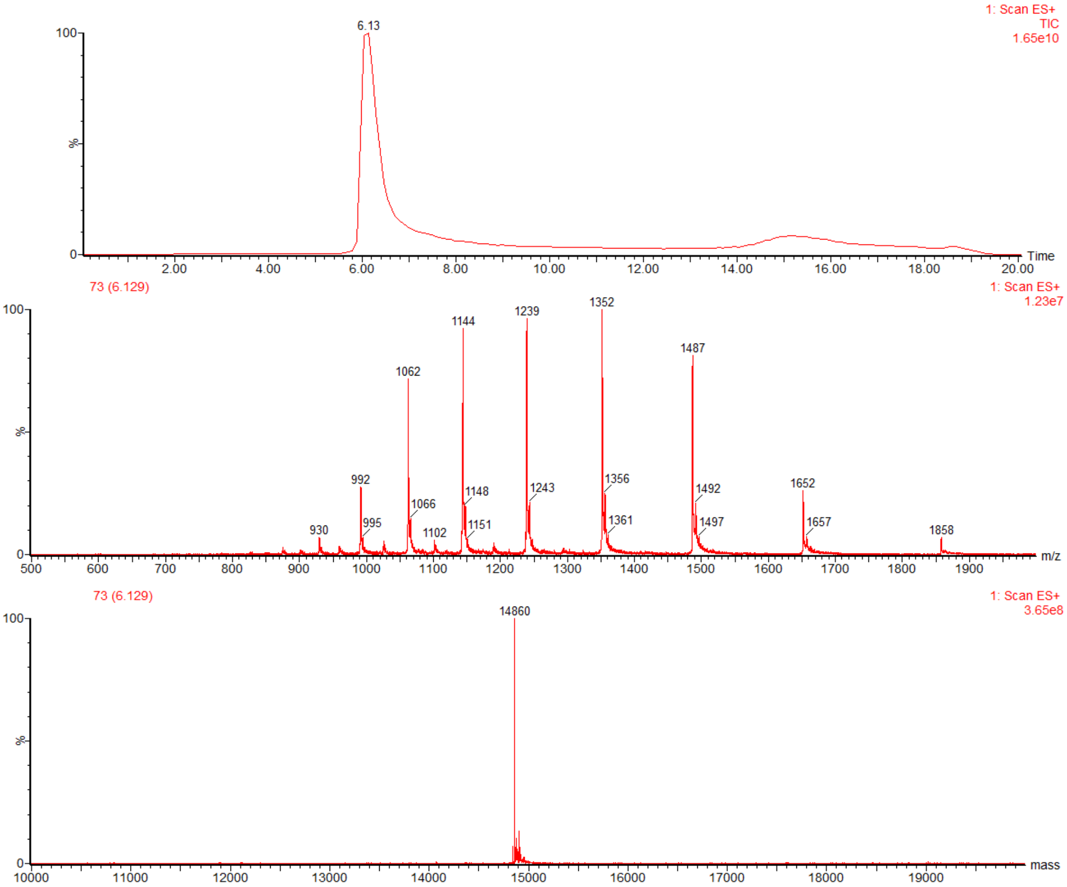


**Figure S14.** LC-MS analysis of a sample of the **2Rb17c-C138** antibody.

**Thiomab LC-V205C** (provided by Genentech)

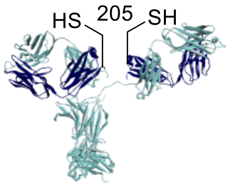


Molecular weight: N/A; observed: LC 23445 Da, HC 50597 Da.

$\varepsilon_{280} = 210896\ L \cdot mol^{-1} \cdot cm^{-1}$

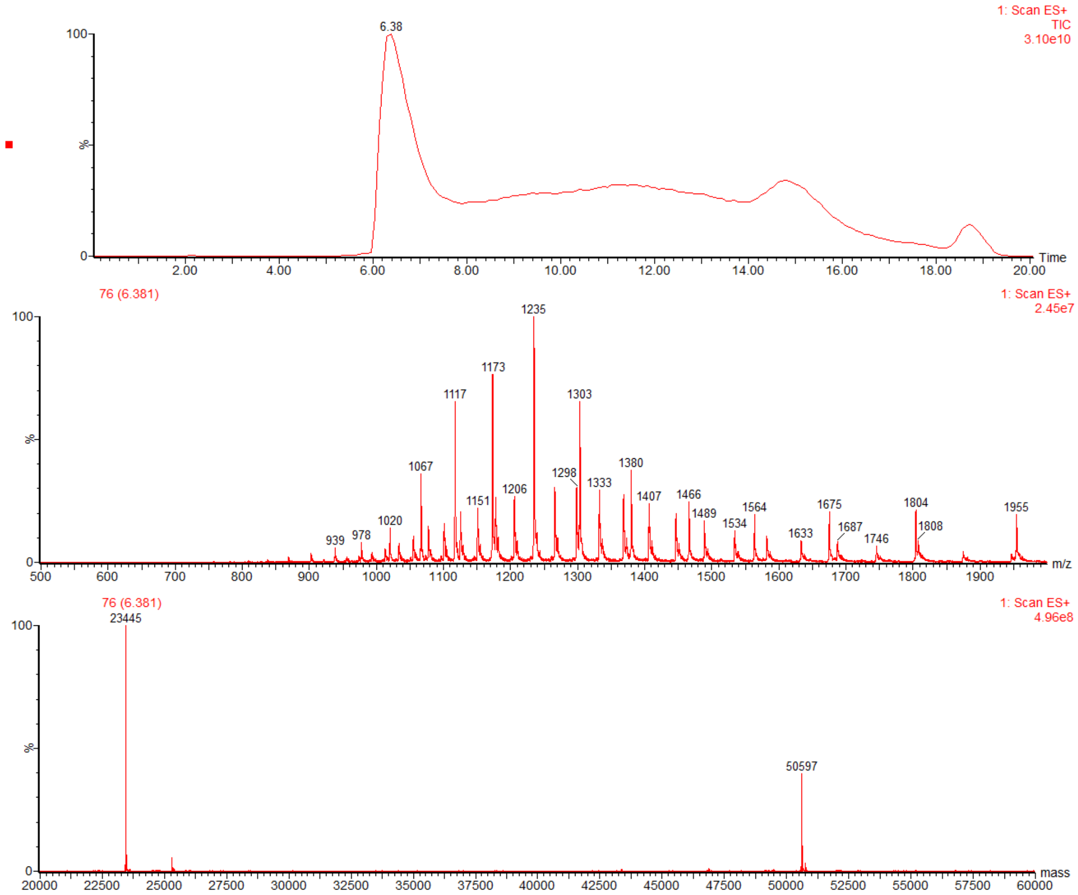


**Figure S15.** LC-MS analysis of a sample of the **Thiomab LC-V205C** antibody.

**Gemtuzumab LC-V205C** (provided by AstraZeneca)

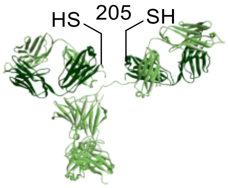


Amino acid sequence LC:

DIQLTQSPSTLSASVGDRVTITCRASESLDNYGIRFLTWFQQKPGKAPKLLMYAASNQGS
GVPSRFSGSGSGTEFTLTISSLQPDDFATYYCQQTKEVPWSFGQGTKVEVKRTVAAPSVF
IFPPSDEQLKSGTASVVCLLNNFYPREAKVQWKVDNALQSGNSQESVTEQDSKDSTYSL
SSTLTLSKADYEKHKVYACEVTHQGLSSP**C**TKSFNRGEC

Amino acid sequence HC:

EVQLVQSGAEVKKPGSSVKVSCKASGYTITDSNIHWVRQAPGQSLEWIGYIYPYNGGTD
YNQKFKNRATLTVDNPTNTAYMELSSLRSEDTAFYYCVNGNPWLAYWGQGTLVTVSS
ASTKGPSVFPLAPSSKSTSGGTAALGCLVKDYFPEPVTVSWNSGALTSGVHTFPAVLQSS
GLYSLSSVVTVPSSSLGTQTYICNVNHKPSNTKVDKRVEPKSCDKTHTCGPPCPAPELLG
GPSVFLFPPKPKDTLMISRTPEVTCVVVDVSHEDPEVKFNWYVDGVEVHNAKTKPREEQ
YNSTYRVVSVLTVLHQDWLNGKEYKCKVSNKALPAPIEKTISKAKGQPREPQVYTLPPS
REEMTKNQVSLTCLVKGFYPSDIAVEWESNGQPENNYKTTPPVLDSDGSFFLYSKLTVD
KSRWQQGNVFSCSVMHEALHNHYTQKSLSLSPGK

Molecular weight: LC 23826 Da, HC 50379; observed: LC 23827 Da, HC 50322 Da.

$\varepsilon_{280} = 220545\ L \cdot mol^{-1} \cdot cm^{-1}$

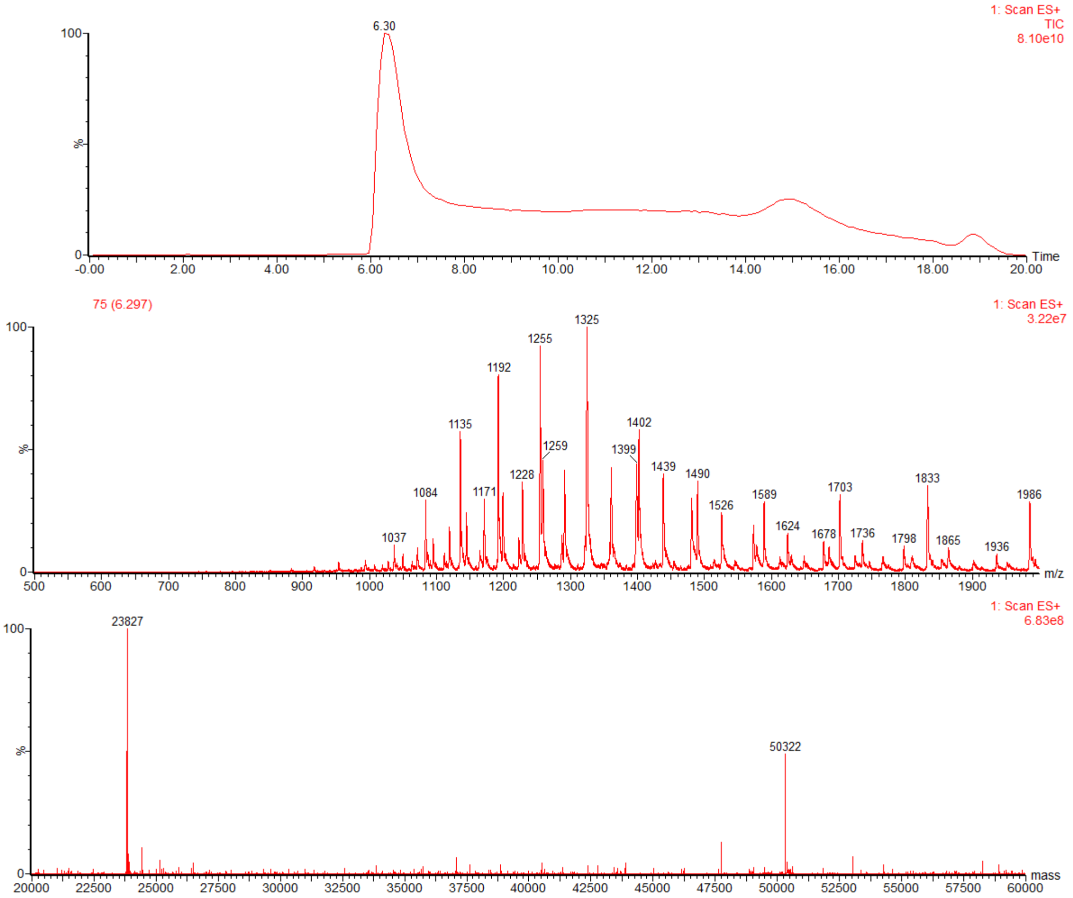


**Figure S16.** LC-MS analysis of a sample of the **Gemtuzumab LC-V205C** antibody.

**Gemtuzumab i239C** (provided by AstraZeneca)

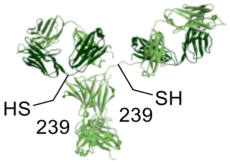


Amino acid sequence LC:

DIQLTQSPSTLSASVGDRVTITCRASESLDNYGIRFLTWFQQKPGKAPKLLMYAASNQGS
GVPSRFSGSGSGTEFTLTISSLQPDDFATYYCQQTKEVPWSFGQGTKVEVKRTVAAPSVF
IFPPSDEQLKSGTASVVCLLNNFYPREAKVQWKVDNALQSGNSQESVTEQDSKDSTYSL
SSTLTLSKADYEKHKVYACEVTHQGLSSPVTKSFNRGEC

Amino acid sequence HC:

EVQLVQSGAEVKKPGSSVKVSCKASGYTITDSNIHWVRQAPGQSLEWIGYIYPYNGGTD
YNQKFKNRATLTVDNPTNTAYMELSSLRSEDTAFYYCVNGNPWLAYWGQGTLVTVSS
ASTKGPSVFPLAPSSKSTSGGTAALGCLVKDYFPEPVTVSWNSGALTSGVHTFPAVLQSS
GLYSLSSVVTVPSSSLGTQTYICNVNHKPSNTKVDKRVEPKSCDKTHTCGPPCPAPELLG
GPS**C**VFLFPPKPKDTLMISRTPEVTCVVVDVSHEDPEVKFNWYVDGVEVHNAKTKPREE
QYNSTYRVVSVLTVLHQDWLNGKEYKCKVSNKALPAPIEKTISKAKGQPREPQVYTLPP
SREEMTKNQVSLTCLVKGFYPSDIAVEWESNGQPENNYKTTPPVLDSDGSFFLYSKLTV
DKSRWQQGNVFSCSVMHEALHNHYTQKSLSLSPGK

Molecular weight: LC 23823 Da, HC 50482 Da; observed: LC 23823 Da, HC 50428 Da.

$\varepsilon_{280} = 220545\ L \cdot mol^{-1} \cdot cm^{-1}$

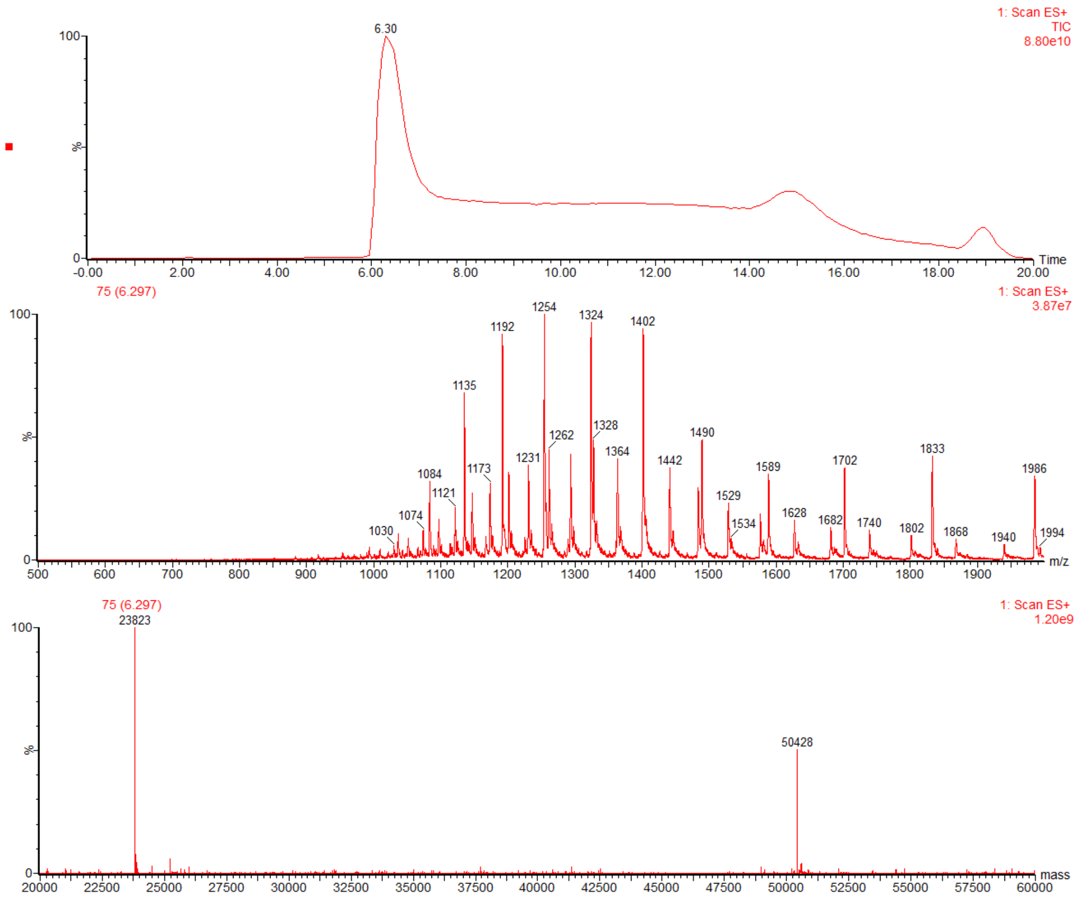


**Figure S17.** LC-MS analysis of a sample of the **Gemtuzumab i239C** antibody.

**Gemtuzumab HC-S442C** (provided by AstraZeneca)

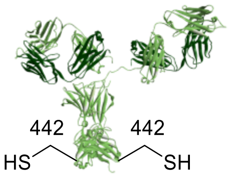


Amino acid sequence LC:

DIQLTQSPSTLSASVGDRVTITCRASESLDNYGIRFLTWFQQKPGKAPKLLMYAASNQGS
GVPSRFSGSGSGTEFTLTISSLQPDDFATYYCQQTKEVPWSFGQGTKVEVKRTVAAPSVF
IFPPSDEQLKSGTASVVCLLNNFYPREAKVQWKVDNALQSGNSQESVTEQDSKDSTYSL
SSTLTLSKADYEKHKVYACEVTHQGLSSPVTKSFNRGEC

Amino acid sequence HC:

EVQLVQSGAEVKKPGSSVKVSCKASGYTITDSNIHWVRQAPGQSLEWIGYIYPYNGGTD
YNQKFKNRATLTVDNPTNTAYMELSSLRSEDTAFYYCVNGNPWLAYWGQGTLVTVSS
ASTKGPSVFPLAPSSKSTSGGTAALGCLVKDYFPEPVTVSWNSGALTSGVHTFPAVLQSS
GLYSLSSVVTVPSSSLGTQTYICNVNHKPSNTKVDKRVEPKSCDKTHTCGPPCPAPELLG
GPSVFLFPPKPKDTLMISRTPEVTCVVVDVSHEDPEVKFNWYVDGVEVHNAKTKPREEQ
YNSTYRVVSVLTVLHQDWLNGKEYKCKVSNKALPAPIEKTISKAKGQPREPQVYTLPPS
REEMTKNQVSLTCLVKGFYPSDIAVEWESNGQPENNYKTTPPVLDSDGSFFLYSKLTVD
KSRWQQGNVFSCSVMHEALHNHYTQKSL**C**LSPGK

Molecular weight: LC 23823 Da, HC 50395 Da; observed: LC 23823 Da, HC 50343 Da.

$\varepsilon_{280}$ = 220545 L · mol$^{-1}$ · cm$^{-1}$

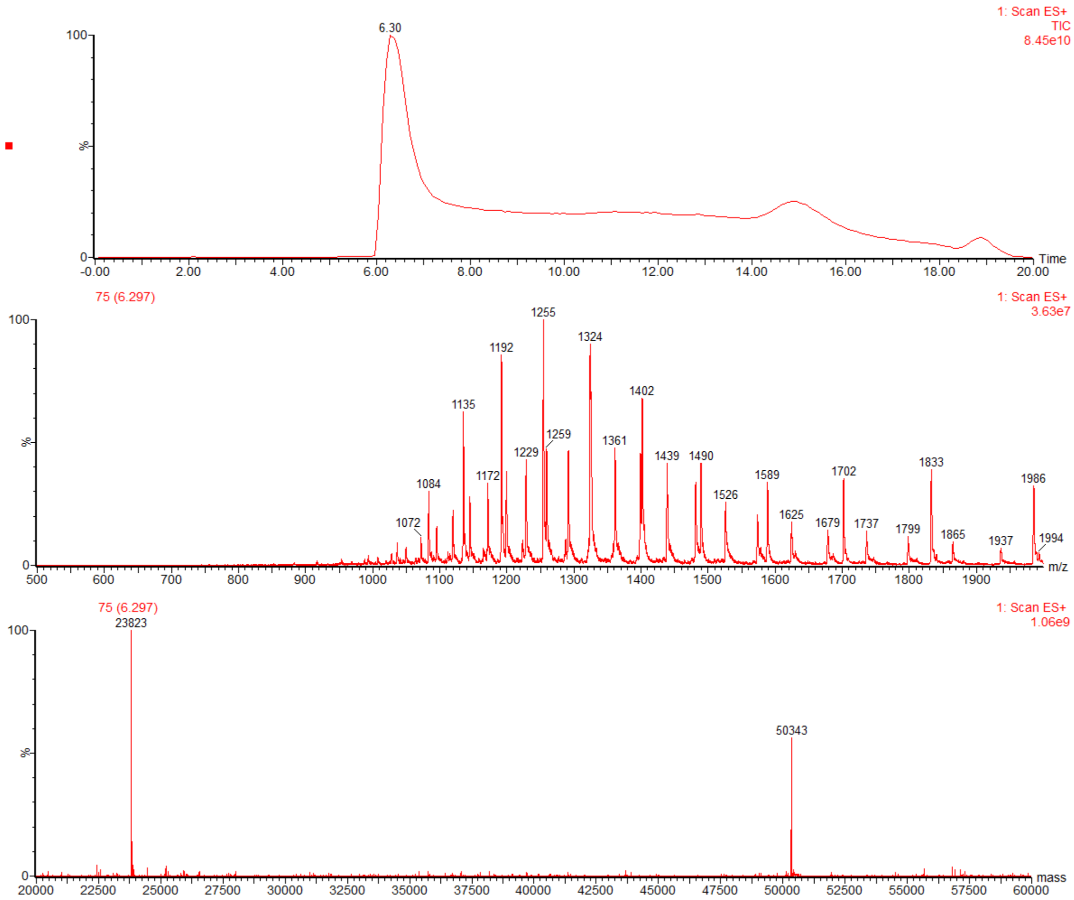


**Figure S18.** LC-MS analysis of a sample of the **Gemtuzumab HC-S442C** antibody.

**Trastuzumab**

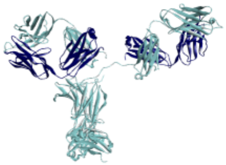

Amino acid sequence LC:

DIQMTQSPSSLSASVGDRVTITCRASQDVNTAVAWYQQKPGKAPKLLIYSASFLYSGVP
SRFSGSRSGTDFTLTISSLQPEDFATYYCQQHYTTPPTFGQGTKVEIKRTVAAPSVFIFPPS
DEQLKSGTASVVCLLNNFYPREAKVQWKVDNALQSGNSQESVTEQDSKDSTYSLSSTL
TLSKADYEKHKVYACEVTHQGLSSPVTKSFNRGEC

Amino acid sequence HC:

EVQLVESGGGLVQPGGSLRLSCAASGFNIKDTYIHWVRQAPGKGLEWVARIYPTNGYT
RYADSVKGRFTISADTSKNTAYLQMNSLRAEDTAVYYCSRWGGDGFYAMDYWGQGT
LVTVSSASTKGPSVFPLAPSSKSTSGGTAALGCLVKDYFPEPVTVSWNSGALTSGVHTFP
AVLQSSGLYSLSSVVTVPSSSLGTQTYICNVNHKPSNTKVDKKVEPKSCDKTHTCPPCPA
PELLGGPSVFLFPPKPKDTLMISRTPEVTCVVVDVSHEDPEVKFNWYVDGVEVHNAKTK
PREEQYNSTYRVVSVLTVLHQDWLNGKEYKCKVSNKALPAPIEKTISKAKGQPREPQV
YTLPPSREEMTKNQVSLTCLVKGFYPSDIAVEWESNGQPENNYKTTPPVLDSDGSFFLYS
KLTVDKSRWQQGNVFSCSVMHEALHNHYTQKSLSLSPGK

Molecular weight: LC 23443 Da, HC 49284; observed: LC 23444 Da, HC 50604 Da.

Note: Molecular weight of HC exceeds expected value due to glycosylation.

$\varepsilon^{280} = 225000\ L \cdot mol^{-1} \cdot cm^{-1}$

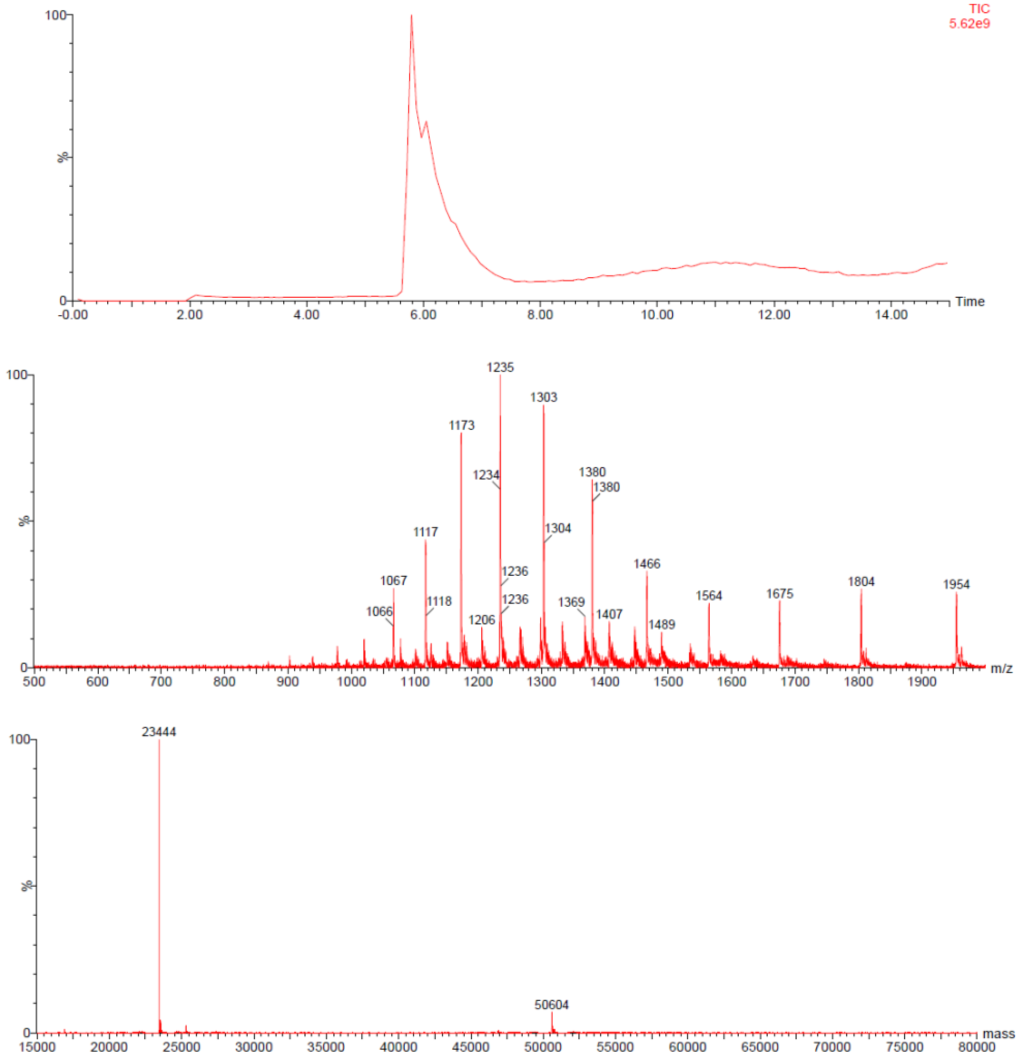


**Figure S19.** LC-MS analysis of a sample of the **Trastuzumab** antibody.

## 5'-$NH_2$ DNA ON BA-labelling reaction optimization

To an Eppendorf tube with **5'-$NH_2$-ss11-mer** ON (50 µM final concentration, 1 equiv.) in 50 mM $NaP_i$ pH 8.0, BA-PFP **2** (100 mM in DMF) and DMF were added. The reaction mixture was vortexed and shaken at 37 ºC, overnight after which 5 µL aliquot of the reaction mixture was analyzed by LC-MS.

**Table S1.** Optimization of DNA **5'-$NH_2$-ss11-mer** ON labelling using BA-PFP reagent **2**.

| BA-PFP **2** (equiv.) | DMF (%) | Conversion[a] |
|---|---|---|
| 50 | 20 | 90% |
| 100 | 10 | 90% |
| 100 | 20 | >95% |
| 200 | 20 | >95% |
| 200 | 30 | >95% |
| 300 | 20 | >95% (~7% double modif.) |

[a] Determined by LC-MS analysis. Highlighted in grey are optimized reaction conditions that were used for the BA-labelling of 5'-$NH_2$-ONs.

BA-labelling reactions can be performed under above optimized reaction conditions in 20 µL–1 mL reaction volume with 25–100 µM final ON concentration, however, 50 µM ON sample concentration was ideal for the LC-MS analysis. Each labelling reaction was attempted at least 3 times under the optimized reaction conditions and similar results were obtained for each ON.

Prepared BA-labelled ONs were purified, and buffer exchanged using illustra™ MicroSpin™ G-25 columns, lyophilised and redissolved in nuclease-free water or buffer of choice for subsequent bioconjugation reactions. Alternatively, BA-labelled ONs can be purified by RP-HPLC. BA-labelled ONs can be stored at 4 ºC for weeks and at -20 ºC for months.

## Preparation of BA-labelled ONs

### 5'-BA-ss11-mer

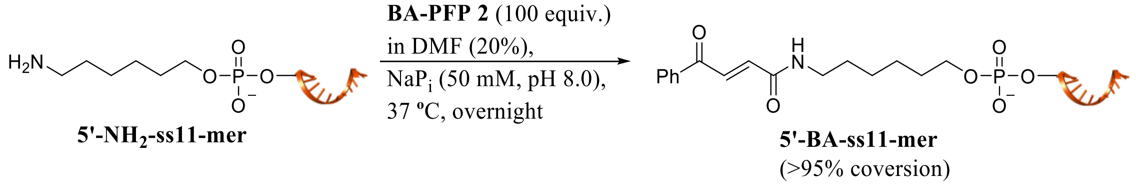


To an Eppendorf tube with **5'-NH$_2$-ss11-mer** ON (50 µM final concentration, 1 equiv.) in 160 µL of 50 mM NaP$_i$ pH 8.0, 10 µL of BA-PFP **2** (100 mM in DMF, 100 equiv.) and 30 µL of DMF (20% final concentration) were added. The reaction mixture was vortexed and shaken at 37 ºC, overnight. 5 µL aliquot of the reaction mixture was analyzed by LC-MS and complete conversion of the starting material to the desired **5'-BA-ss11-mer** ON product was obtained (calculated: 3627 Da; observed: 3629 Da).

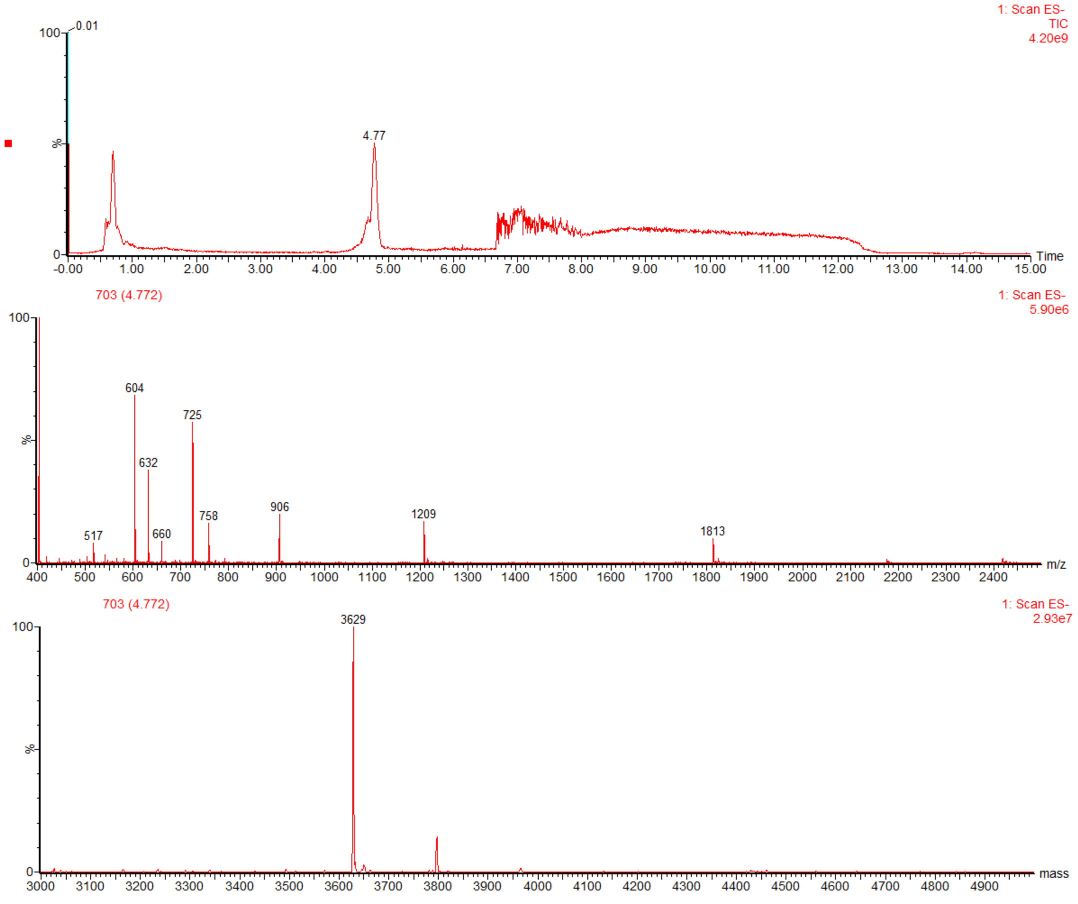


**Figure S20.** LC-MS analysis of the reaction mixture from the preparation of the **5'-BA-ss11-mer** ON.

**5'-BA-ss25-mer**

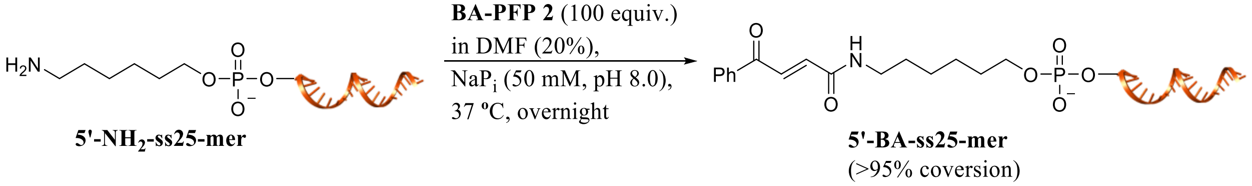


To an Eppendorf tube with **5'-NH₂-ss25-mer** ON (50 μM final concentration, 1 equiv.) in 160 μL of 50 mM NaP$_i$ pH 8.0, 10 μL of BA-PFP **2** (100 mM in DMF, 100 eq.) and 30 μL of DMF (20% final concentration) were added. The reaction mixture was vortexed and shaken at 37 ºC, overnight. 5 μL aliquot of the reaction mixture was analyzed by LC-MS and complete conversion of the starting material to the desired **5'-BA-ss25-mer** ON product was obtained (calculated: 7998 Da; observed: 8001 Da).

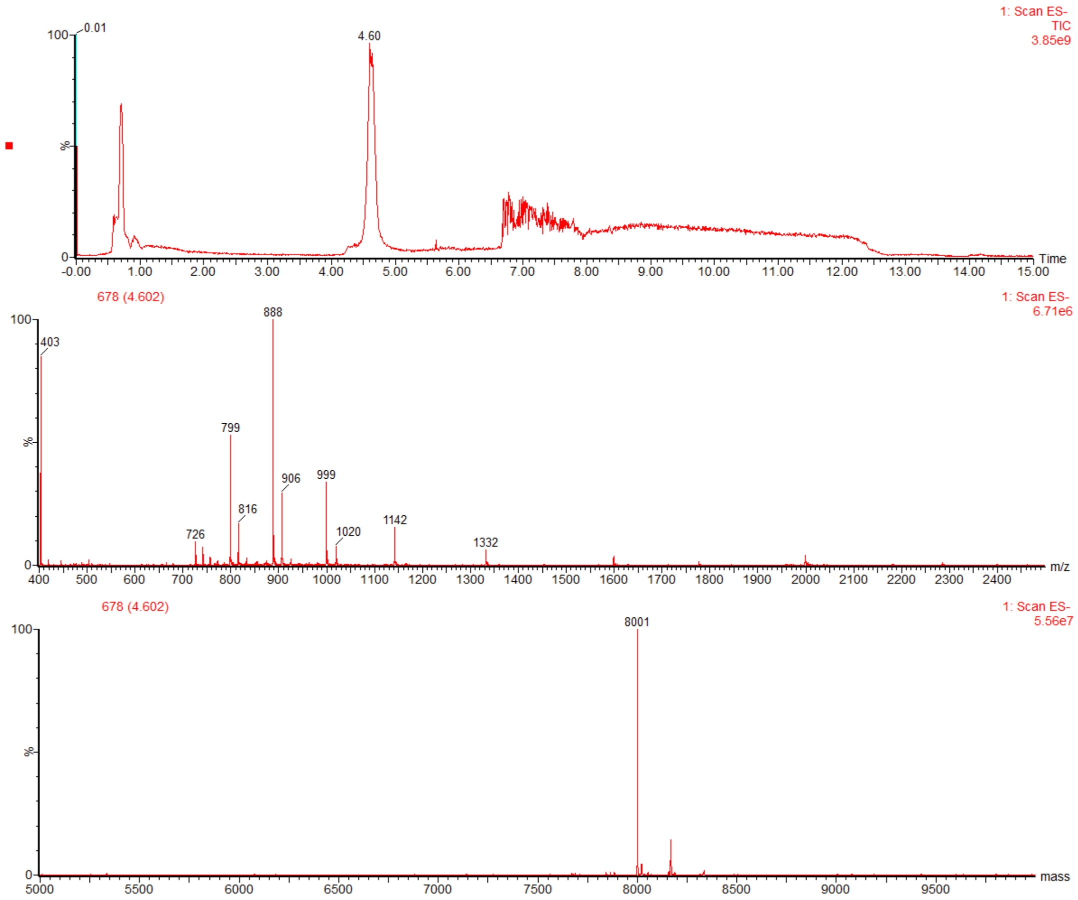


**Figure S21.** LC-MS analysis of the reaction mixture from the preparation of the **5'-BA-ss25-mer** ON.

## 5'-BA-ss50-mer

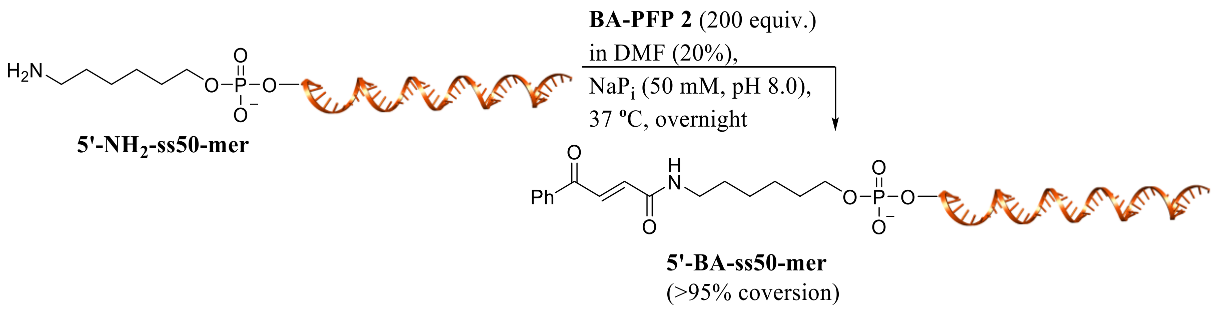


To an Eppendorf tube with **5'-NH$_2$-ss50-mer** ON (30 µM final concentration, 1 equiv.) in 124 µL of 50 mM NaP$_i$ pH 8.0, 9.3 µL of BA-PFP **2** (100 mM in DMF, 200 equiv.) and 21.7 µL of DMF (20% final concentration) were added. The reaction mixture was vortexed and shaken at 37 ºC, overnight. 5 µL aliquot of the reaction mixture was analyzed by LC-MS and complete conversion of the starting material to the desired **5'-BA-ss50-mer** ON product was obtained (calculated: 15683 Da; observed: 15691 Da).

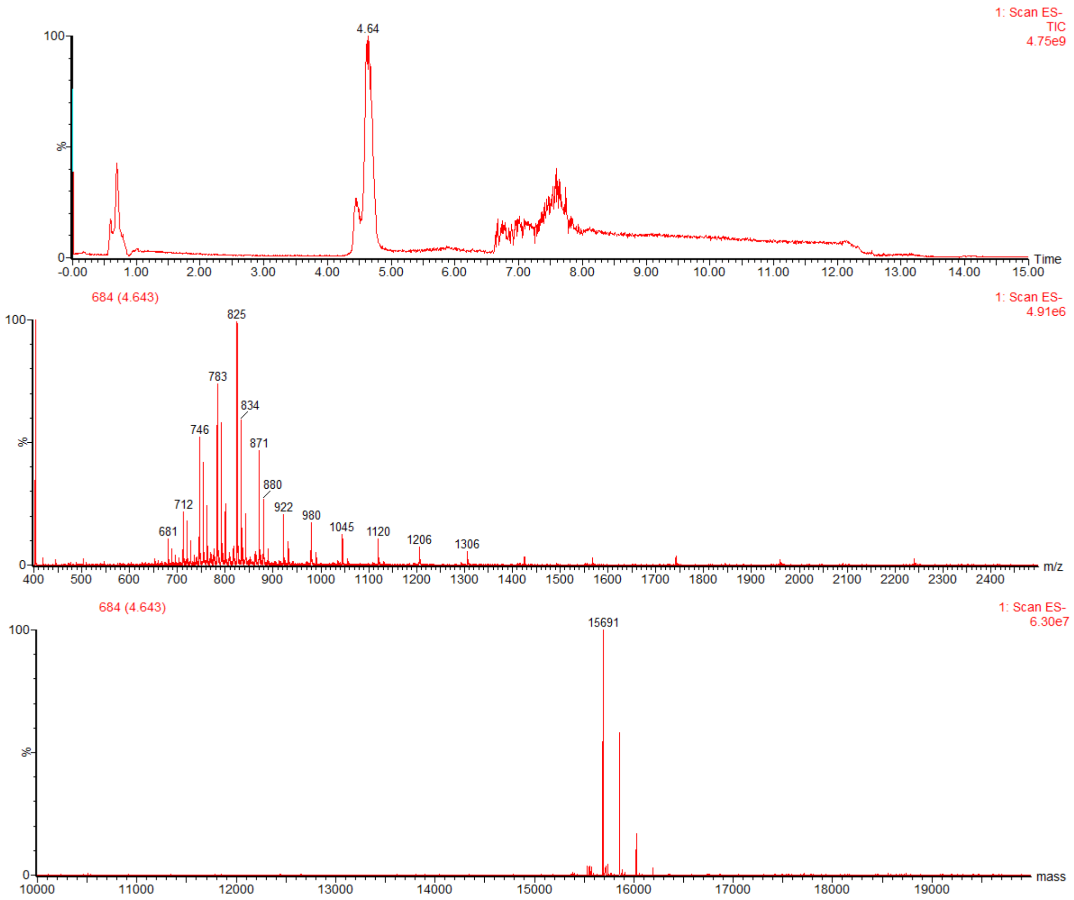


**Figure S22.** LC-MS analysis of the reaction mixture from the preparation of the **5'-BA-ss50-mer** ON.

**5'-BA-ds19-mer**

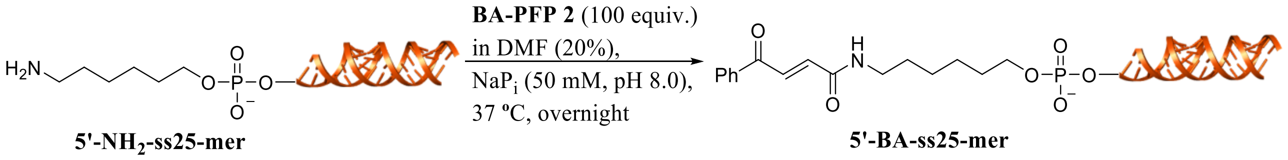


To an Eppendorf tube with **5'-NH₂-ds19-mer** ON (50 µM final concentration, 1 equiv.) in 240 µL of 50 mM $NaP_i$ pH 8.0, 60 µL of BA-PFP **2** (25 mM in DMF, 100 equiv., 20% DMF final concentration) were added. The reaction mixture was vortexed and shaken at 37 ºC, overnight. 5 µL aliquot of the reaction mixture was analyzed by LC-MS and complete conversion of the starting material to the desired **5'-BA-ds19-mer** ON product was obtained (calculated: (sense) 6134 Da, (antisense) 5824 Da; observed: (sense) 6134 Da, (antisense) 5824 Da).

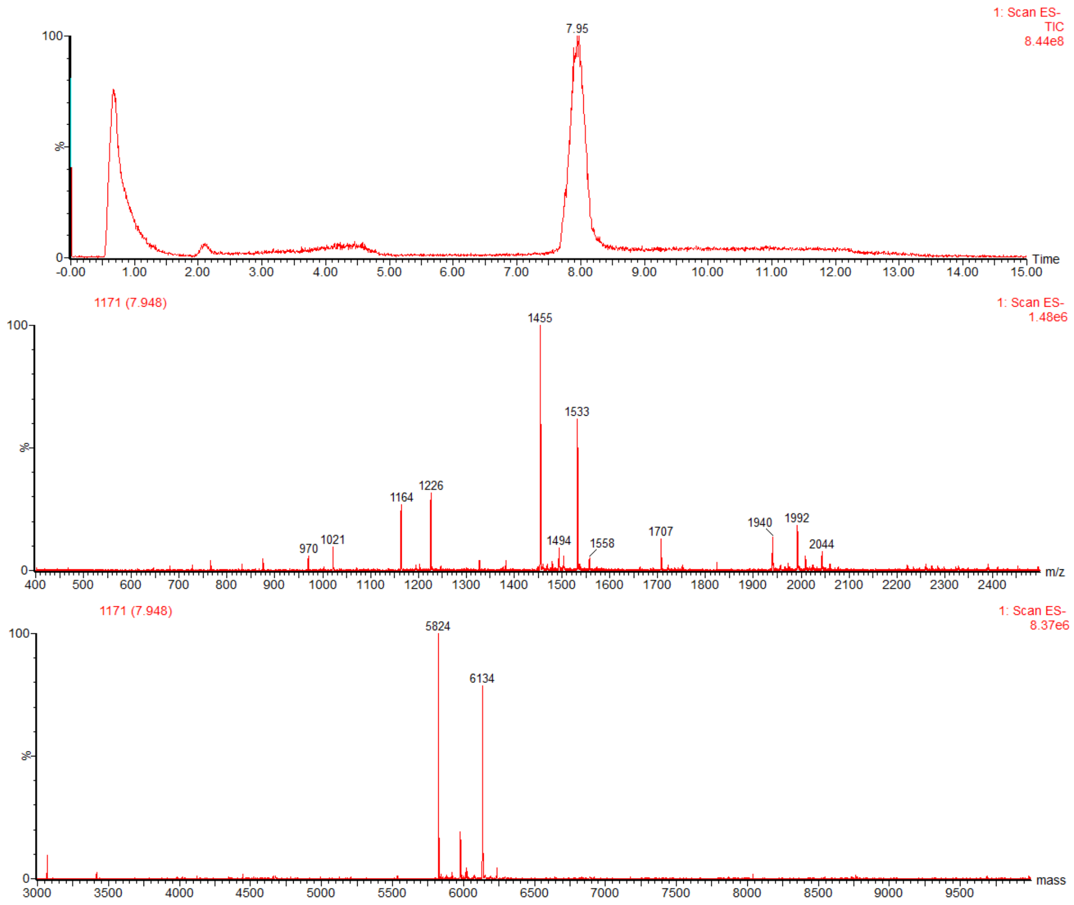


**Figure S23.** LC-MS analysis of the reaction mixture from the preparation of the **5'-BA-ds19-mer** ON.

## 5'-BA-Cy3-3'-ss25-mer

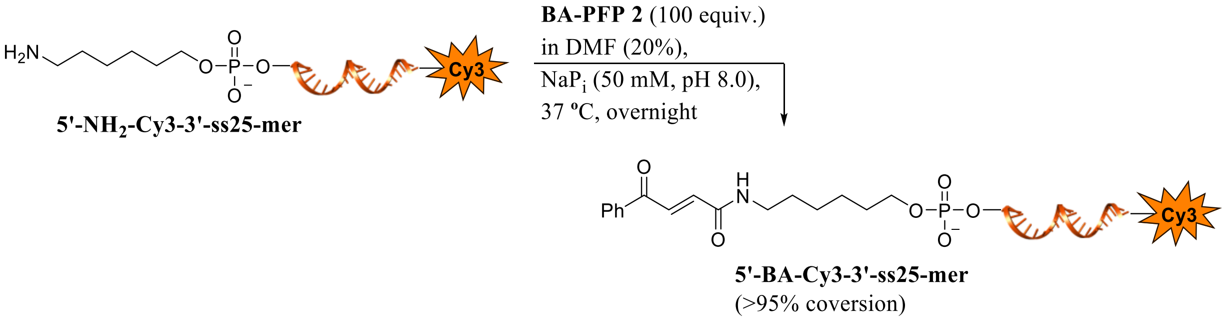


To an Eppendorf tube with **5'-NH₂-Cy3-3'-ss25-mer** ON (50 µM final concentration, 1 equiv.) in 400 µL of 50 mM $NaP_i$ pH 8.0, 100 µL of BA-PFP **2** (25 mM in DMF, 100 equiv., 20% DMF final concentration) were added. The reaction mixture was vortexed and shaken at 37 ºC, overnight. 5 µL aliquot of the reaction mixture was analyzed by LC-MS and complete conversion of the starting material to the desired **5'-BA-Cy3-3'-ss25-mer** ON product was obtained (calculated: 8643 Da; observed: 8645 Da).

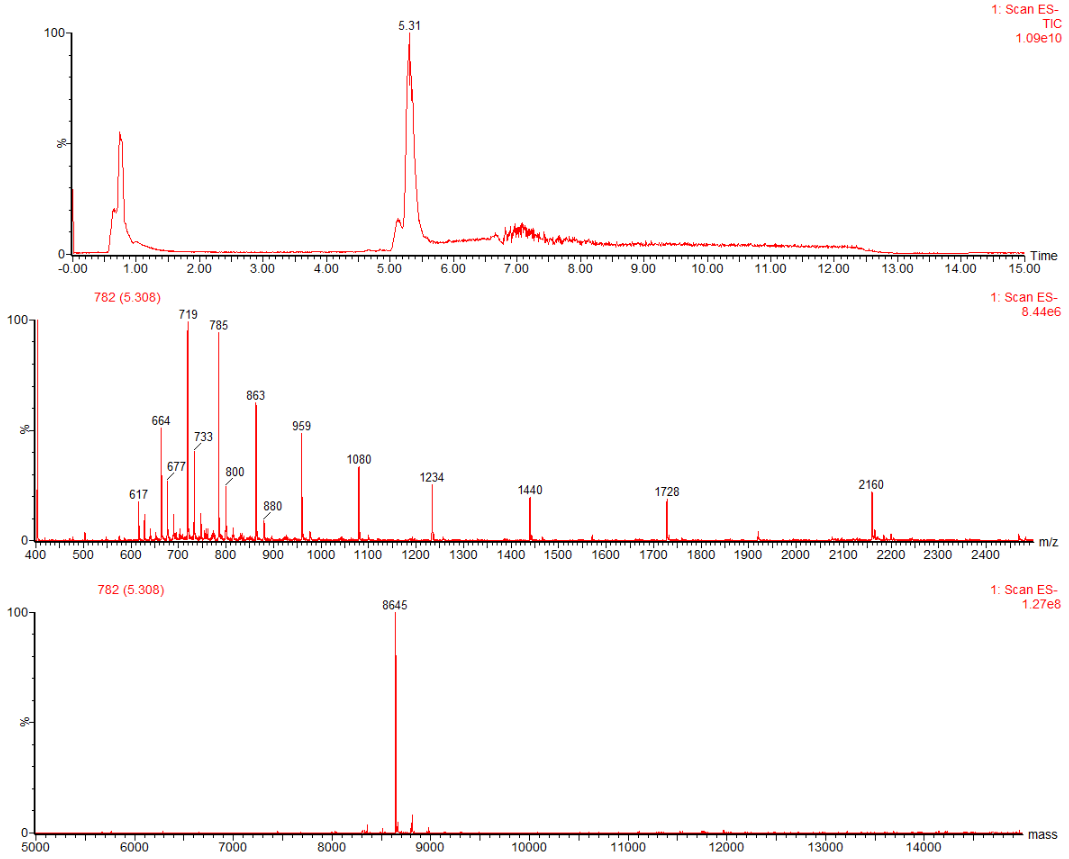


**Figure S24.** LC-MS analysis of the reaction mixture from the preparation of the **5'-BA-Cy3-3'-ss25-mer** ON.

## Preparation of maleimide-labelled ONs

### 5’-Maleimide-ss11-mer + 5’-maleamic acid-ss11-mer (RMss11)

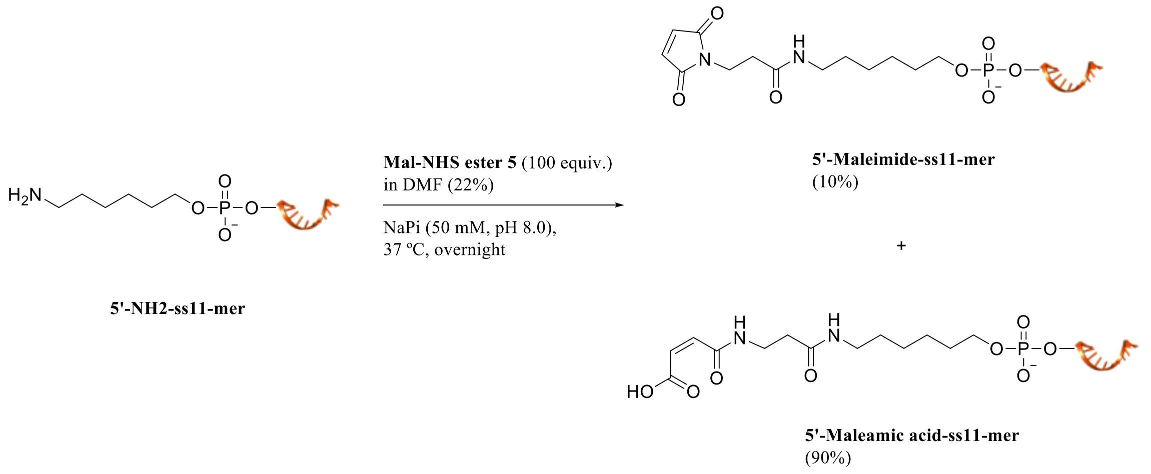


To an Eppendorf tube with **5’-NH$_2$-ss11-mer** ON (50 µM final concentration, 1 equiv.) in 355 µL of 50 mM NaPi pH 8.0, 100 µL of **3-(maleimido)propionic acid *N*-hydroxysuccinimide ester 5** (Mal-NHS ester, 25 mM in DMF, 100 equiv., 22% DMF final concentration) was added. The reaction mixture was vortexed and shaken at 37 ºC, overnight. 5 µL aliquot of the reaction mixture was analysed by LC-MS. Complete conversion of the starting material to ON reaction mixture **RMss11** - 10% **5’-maleimide-ss11-mer** (calculated: 3620 Da; observed: 3622 Da) and 90% **5’-maleamic acid-ss11-mer** (calculated: 3638 Da; observed: 3641 Da) - was obtained.

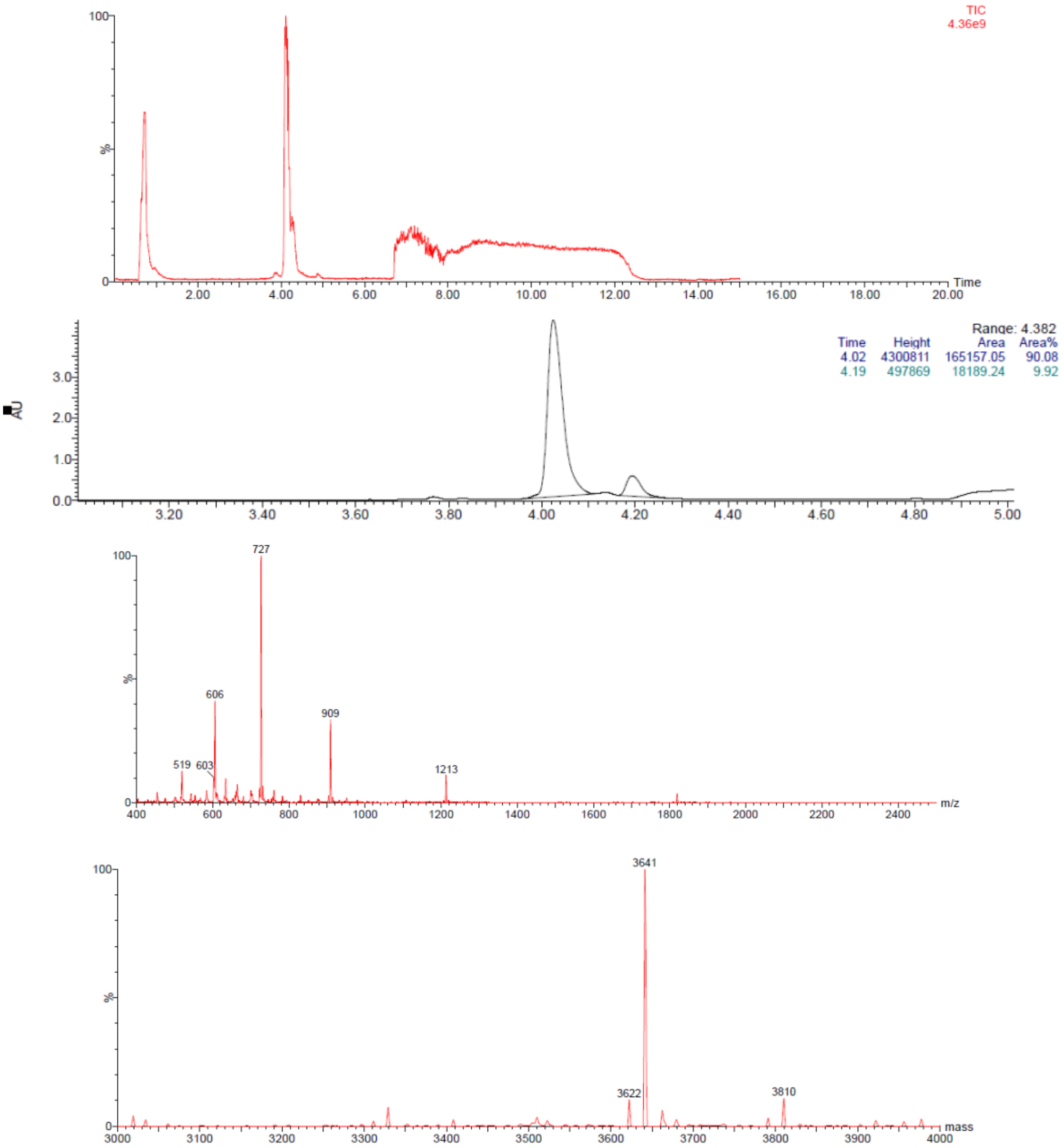


**Figure S25.** LC-MS analysis of the reaction mixture from the preparation of **5'-maleimide-ss11-mer** ON.

**5’-Maleimide-ss25-mer + 5’-maleamic acid-ss25-mer (RMss25)**

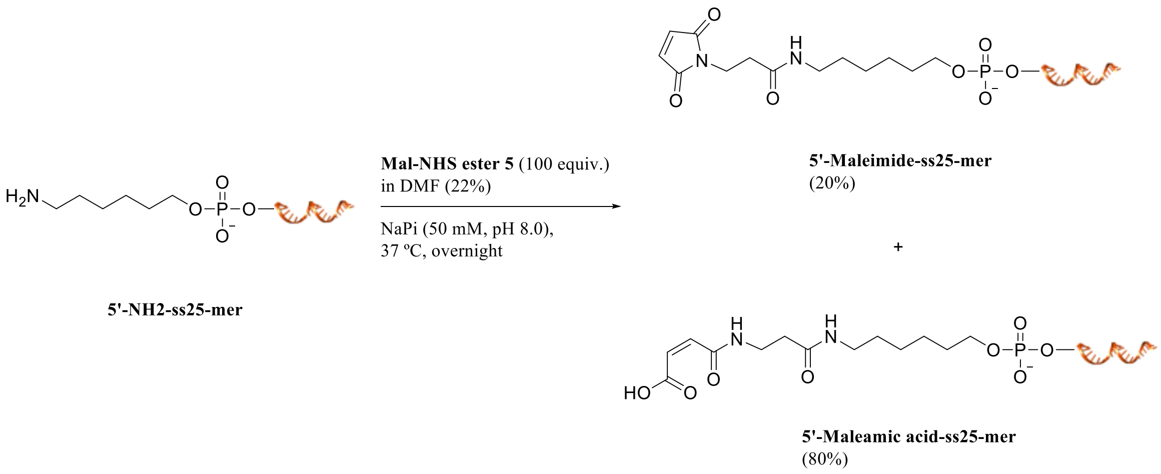


To an Eppendorf tube with **5’-NH$_2$-ss25-mer** ON (50 µM final concentration, 1 equiv.) in 355 µL of 50 mM NaPi pH 8.0, 100 µL of **3-(maleimido)propionic acid *N*-hydroxysuccinimide ester 5** (Mal-NHS ester, 25 mM in DMF, 100 equiv. 22% DMF final concentration) was added. The reaction mixture was vortexed and shaken at 37 ºC, overnight. 5 µL aliquot of the reaction mixture was analysed by LC-MS. Complete conversion of the starting material to ON reaction mixture **RMss25** - 21% **5’-Maleimide-ss25-mer** (calculated: 7991 Da; observed: 7993 Da) and 79% **5’-Maleamic acid-ss25-mer** (calculated: 8009 Da; observed: 8011 Da) - was obtained.

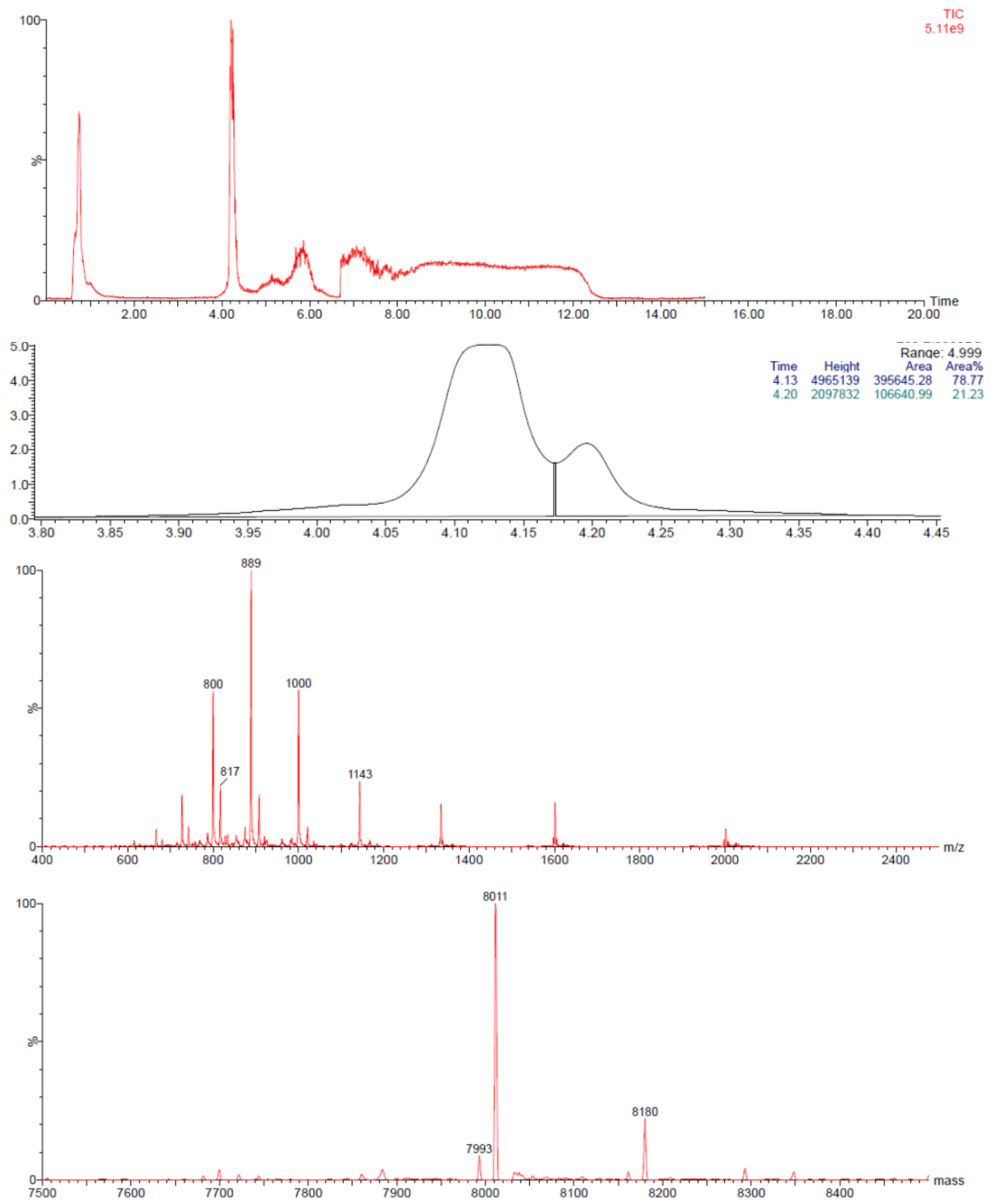


**Figure S26.** LC-MS analysis of the reaction mixture from the preparation of **5'-maleimide-ss25-mer** ON.

## Hydrolytic stability of maleimide vs BA-labelled ONs

### 5'-Maleimide-ss11-mer

**RMss11** (20 µM final concentration) in 20 µL of 50 mM NaPi pH 8.0 was shaken for 24 h at 37 ºC. 5 µL of the reaction mixture was analysed by LC-MS. Complete hydrolysis of **5'-maleimide-ss11-mer** to **5'-maleamic acid-ss11-mer** was obtained (calculated: 3638 Da; observed: 3641 Da).

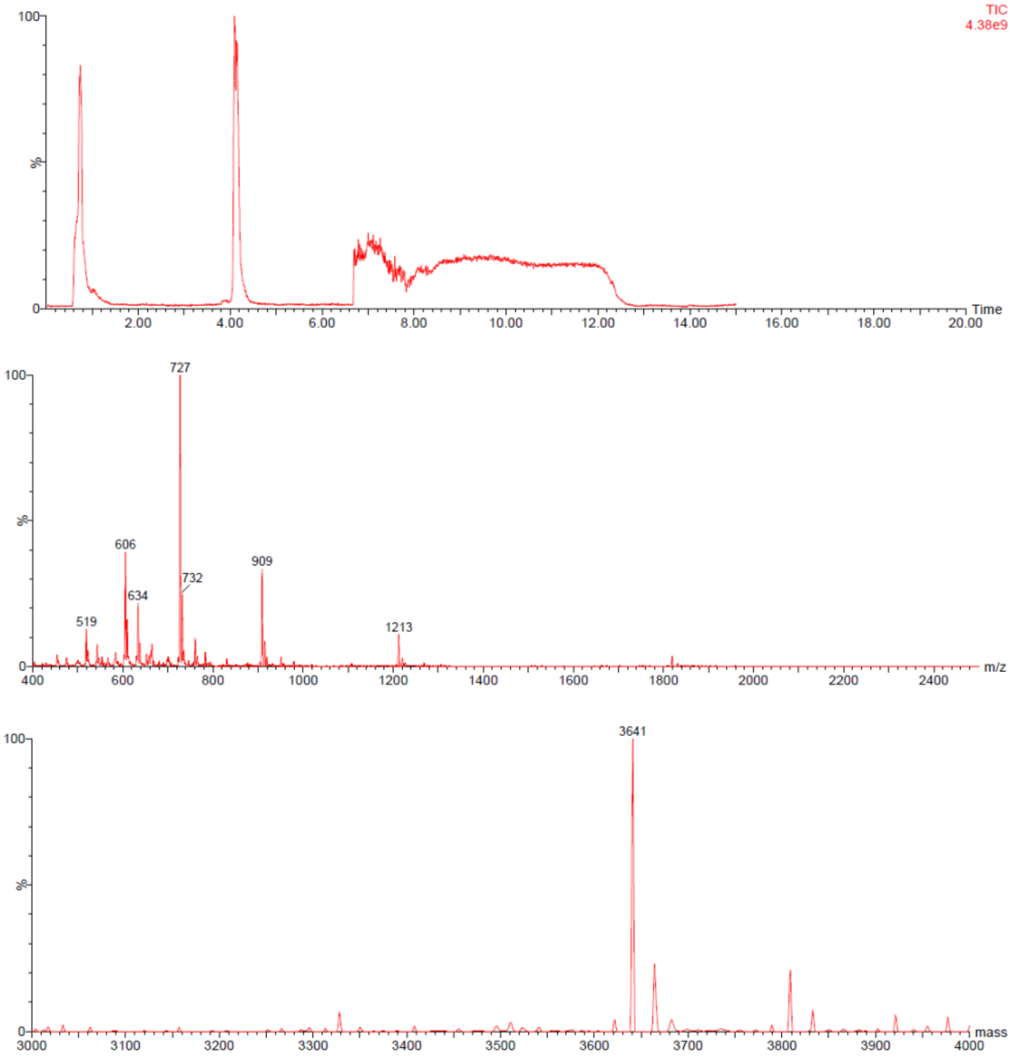


**Figure S27.** LC-MS analysis of the reaction mixture from the preparation of **5'maleimide-ss11-mer** after 24 h at 37 ºC.

Control reaction between **2Rb17c-C138** and **5'-maleamic acid-ss11-mer**

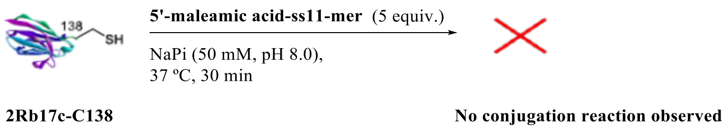


In an Eppendorf tube, the reaction mixture consisting of freshly reduced **2Rb17c-C138** (15 µM final concentration, 1 equiv.) and **5'-maleamic acid-ss11-mer** (75 µM final concentration, 5 equiv.) in 14 µL of 50 mM NaPi pH 8.0 was vortexed and shaken at 37 ºC for 30 min. 5 µL aliquot of the reaction mixture was then analysed by LC-MS and only the starting material **2Rb17c-C138** was obtained (calculated: 14861 Da; observed: 14859 Da).

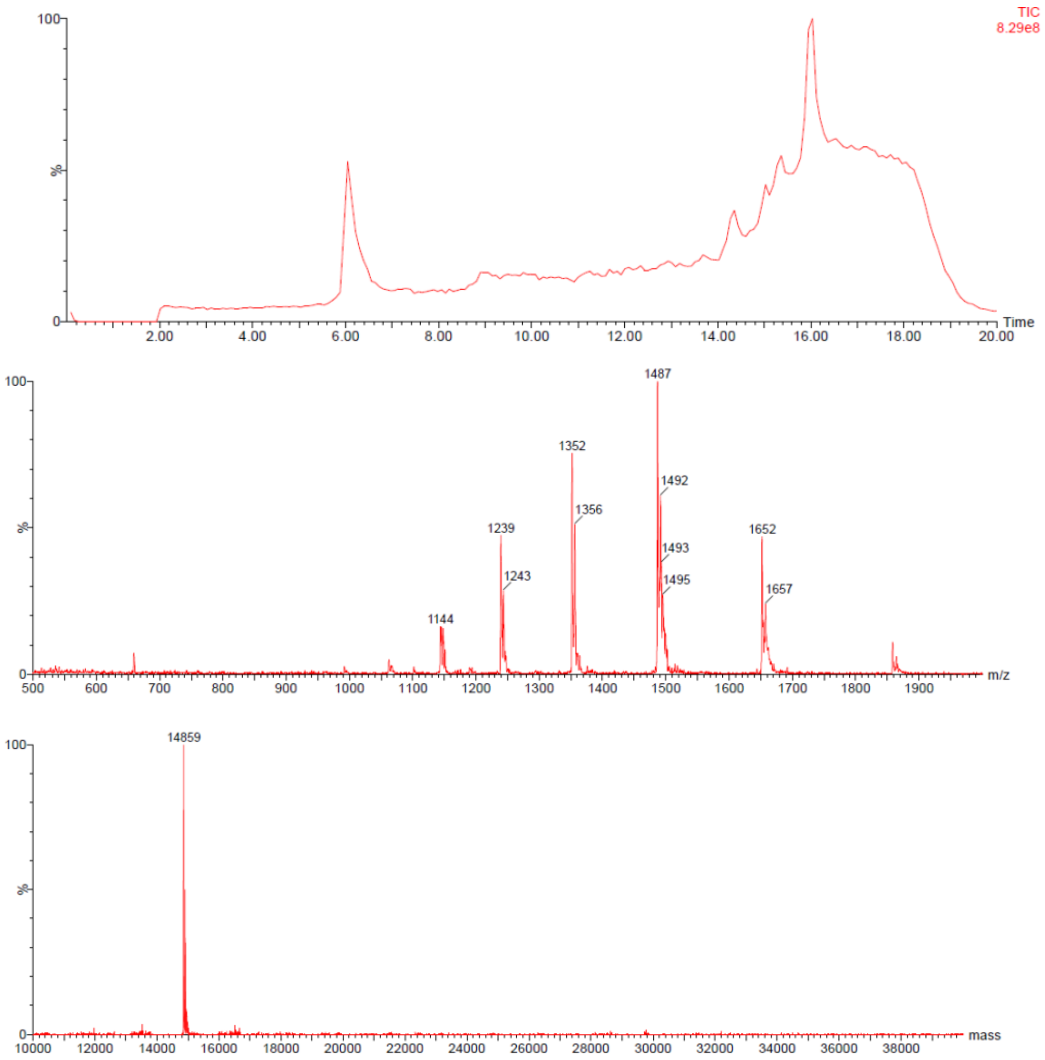


**Figure S28.** LC-MS analysis of the reaction mixture from the reaction between **2Rb17c-C138** and **5'-maleamic acid-ss11-mer**.

**5’-BA-ss11-mer**

**5’-BA-ss11-mer** (20 µM final concentration) in 20 µL of 50 mM NaPi pH 8.0 was shaken for 24 h at 37 ºC. 5 µL of the reaction mixture was analyzed by LC-MS and only the starting material **5’-BA-ss11-mer** was observed (calculated: 3627 Da; observed: 3629 Da).

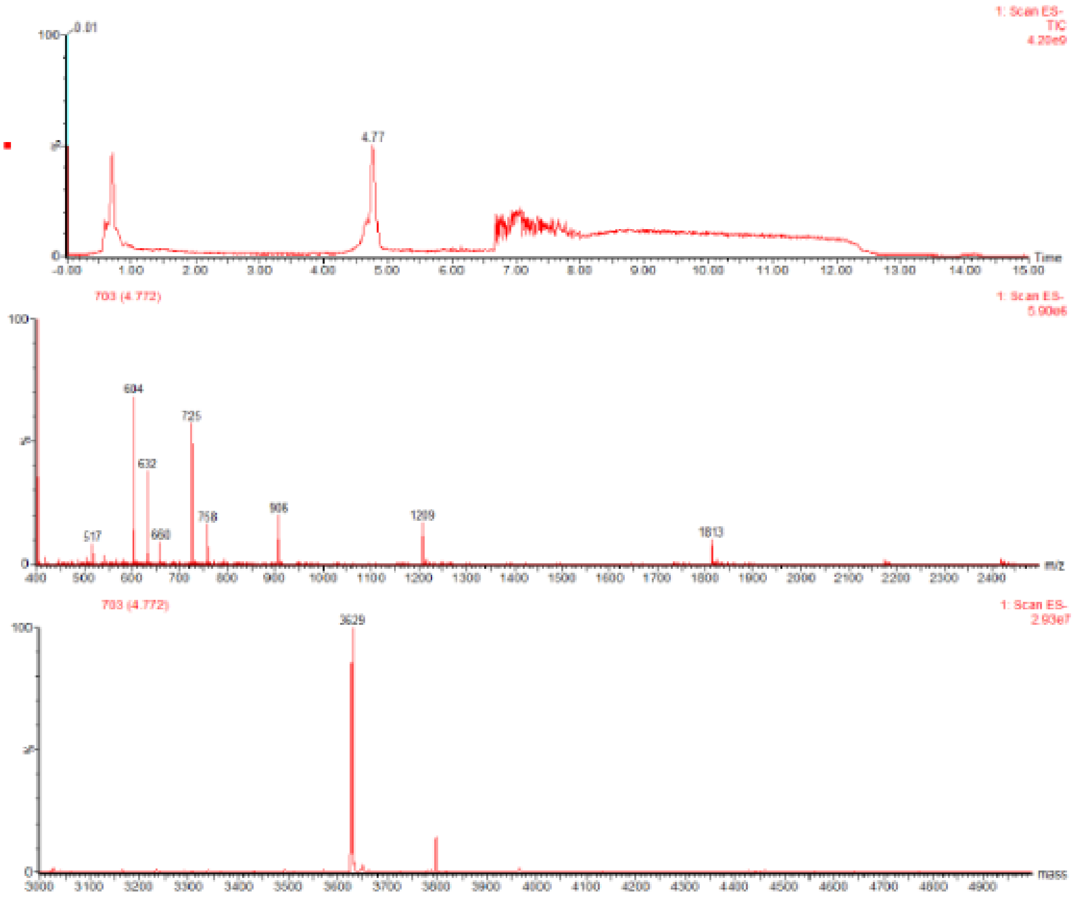


**Figure S29.** LC-MS analysis of the reaction mixture from the preparation of **5’BA-ss11-mer** after 24 h at 37 ºC.

## DNA-protein conjugate LC-MS method optimization

Preparation of **594Nup98 G85C-ss11-mer** conjugate for the LC-MS method optimization

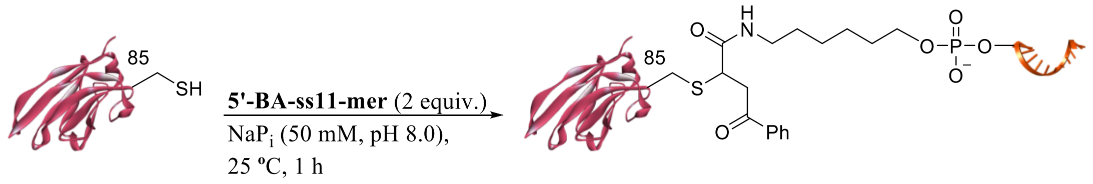


**594Nup98 G85C** **594Nup98 G85C-ss11-mer conjugate** (>95% conversion)

In an Eppendorf tube, the reaction mixture consisting of **594Nup98 G85C** (20 μM final concentration, 1 equiv.) and **5'-BA-ss11-mer** (40 μM final concentration, 2 equiv.) in 90 μL of 50 mM $NaP_i$ pH 8.0 was vortexed and shaken at 25 ºC for 1 h. 5 μL aliquots of the reaction mixture were then analyzed by LC-MS and complete conversion of the starting material to the desired **594Nup98 G85C-ss11-mer** conjugate product was obtained (calculated: 17513 Da; observed: 17516 Da).

**594Nup98 G85C-ss11-mer** conjugate was prepared as a model conjugate to test the effect of the mobile phase additive on the LC-MS analysis. At first, standard LC-MS conditions for the analysis of protein bioconjugates with formic acid as the LC-MS additive were tested and deconvolution of obtained ion series led to multiple peaks with masses corresponding to the expected product (~17.5 kDa).

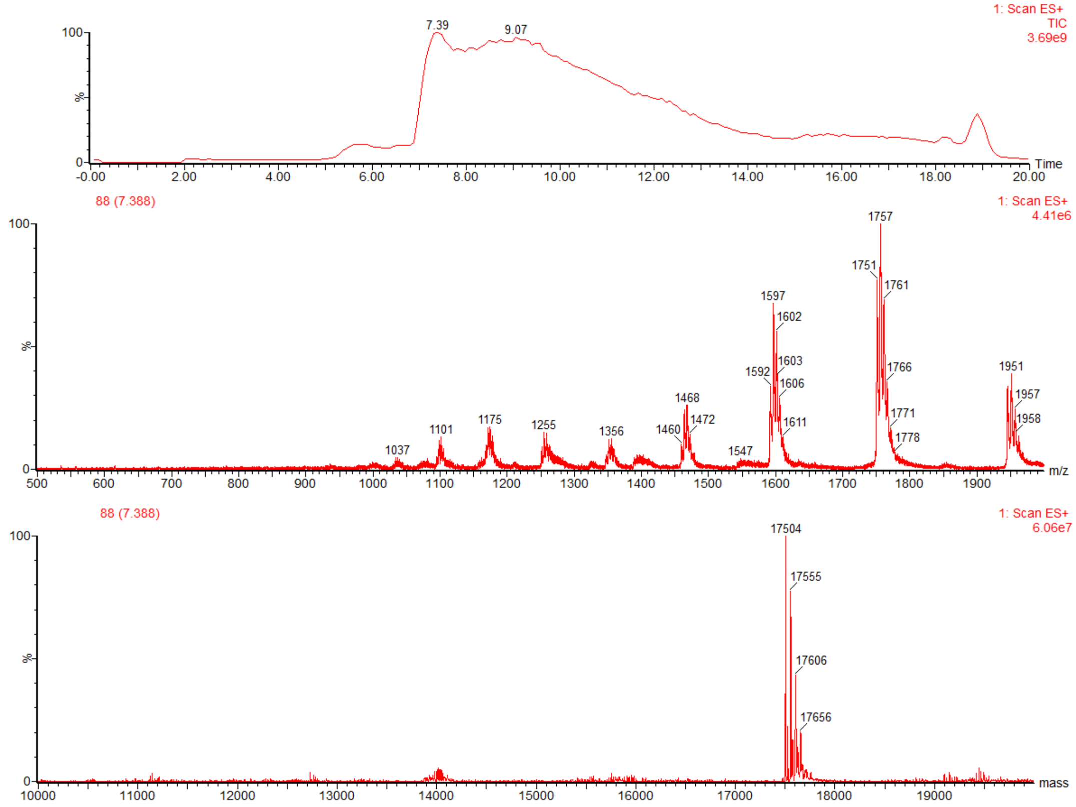


**Figure S30.** LC-MS analysis of the reaction mixture from the preparation of the **594Nup98 G85C-ss11-mer** conjugate with formic acid as the LC-MS mobile phase additive.

Without any LC-MS additive, the conjugate was not effectively ionized, and no ion series was obtained.

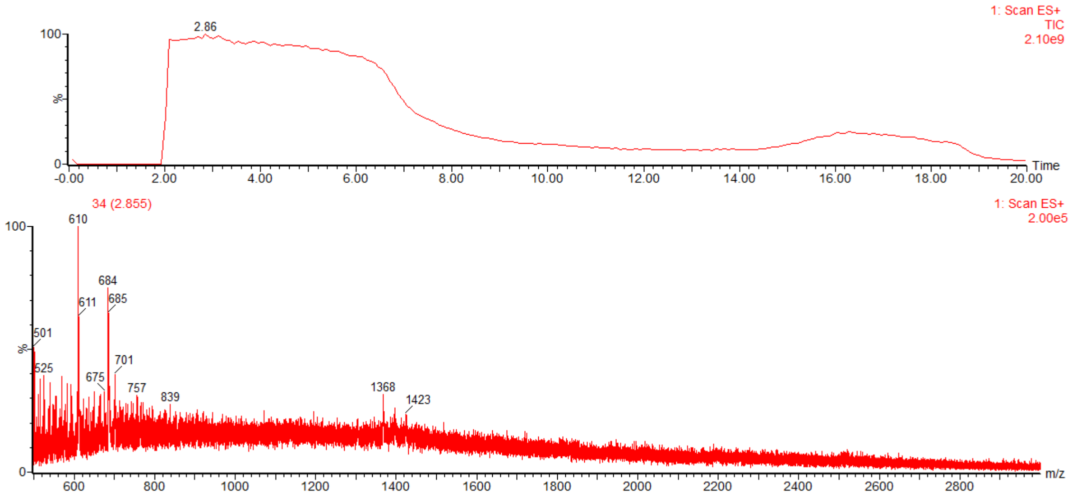


**Figure S31.** LC-MS analysis of the reaction mixture from the preparation of the **594Nup98 G85C-ss11-mer** conjugate without any LC-MS mobile phase additive.

When 10 mM ammonium acetate was used as the LC-MS additive, much cleaner extracted ion series was obtained, and the deconvolution gave a single peak with mass corresponding to the calculated mass of the desired conjugate (17516 Da).

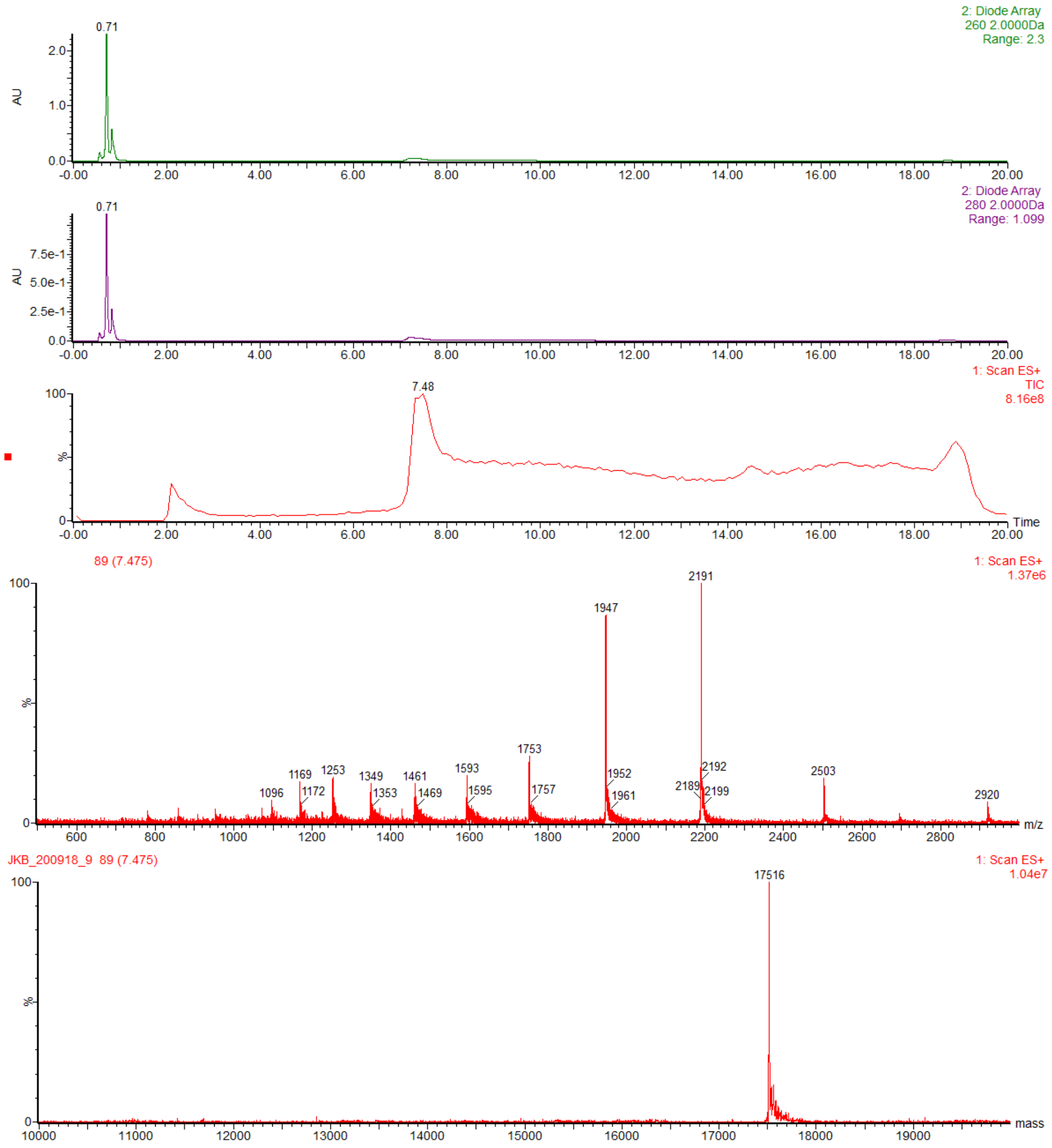


**Figure S32.** LC-MS analysis of the reaction mixture from the preparation of the **594Nup98 G85C-ss11-mer** conjugate with 10 mM ammonium acetate as the LC-MS mobile phase additive.

Lower concentrations of ammonium acetate as the LC-MS additive gave similar results in the signal quality of the extracted ion series or deconvoluted mass spectrum. However, the use of lower additive concentration (5 mM, 2 mM or 1 mM) was noticeable in the UV trace of LC where lower background noise was detected.

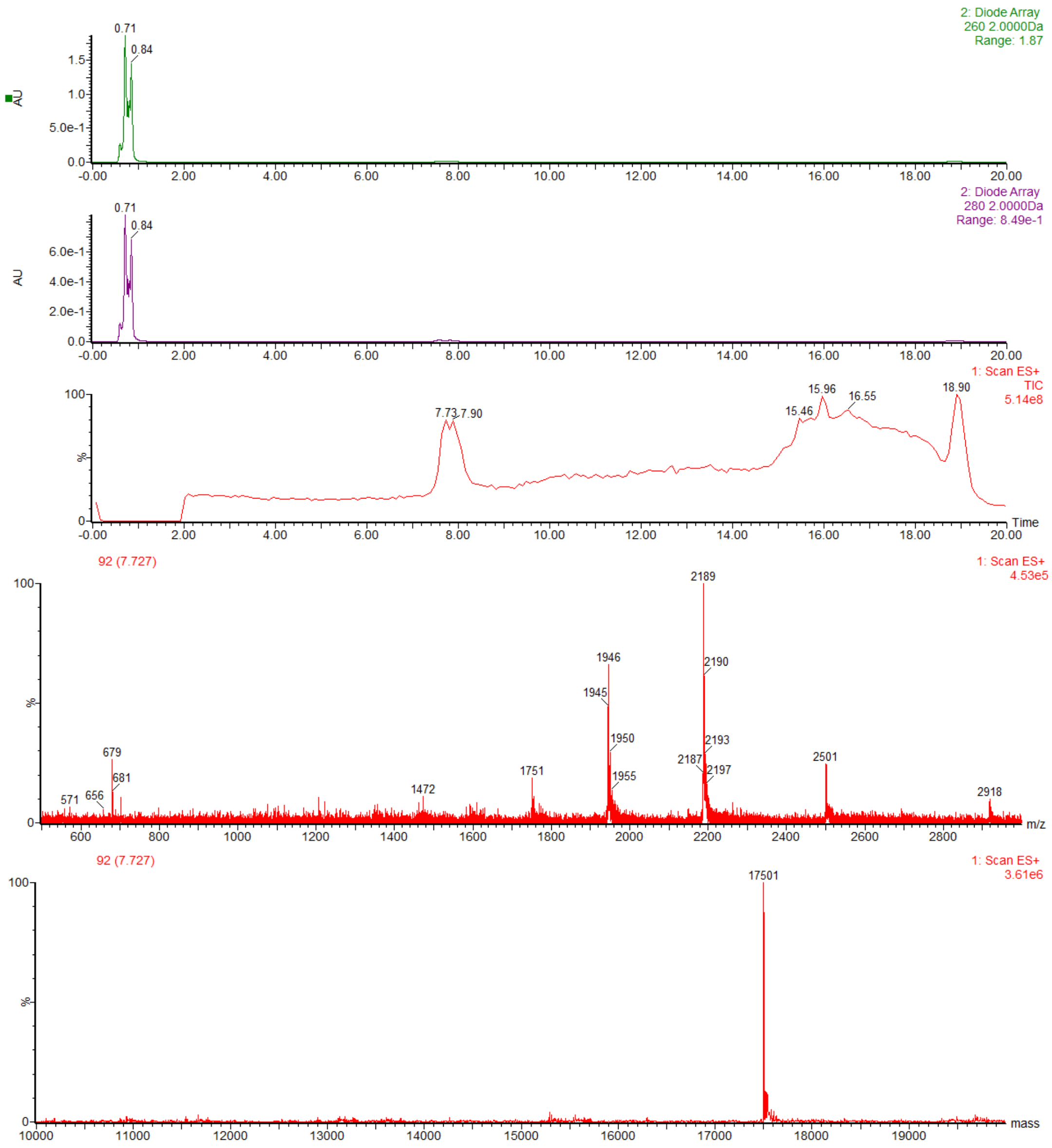


**Figure S33.** LC-MS analysis of the reaction mixture from the preparation of the **594Nup98 G85C-ss11-mer** conjugate with 5 mM ammonium acetate as the LC-MS mobile phase additive.

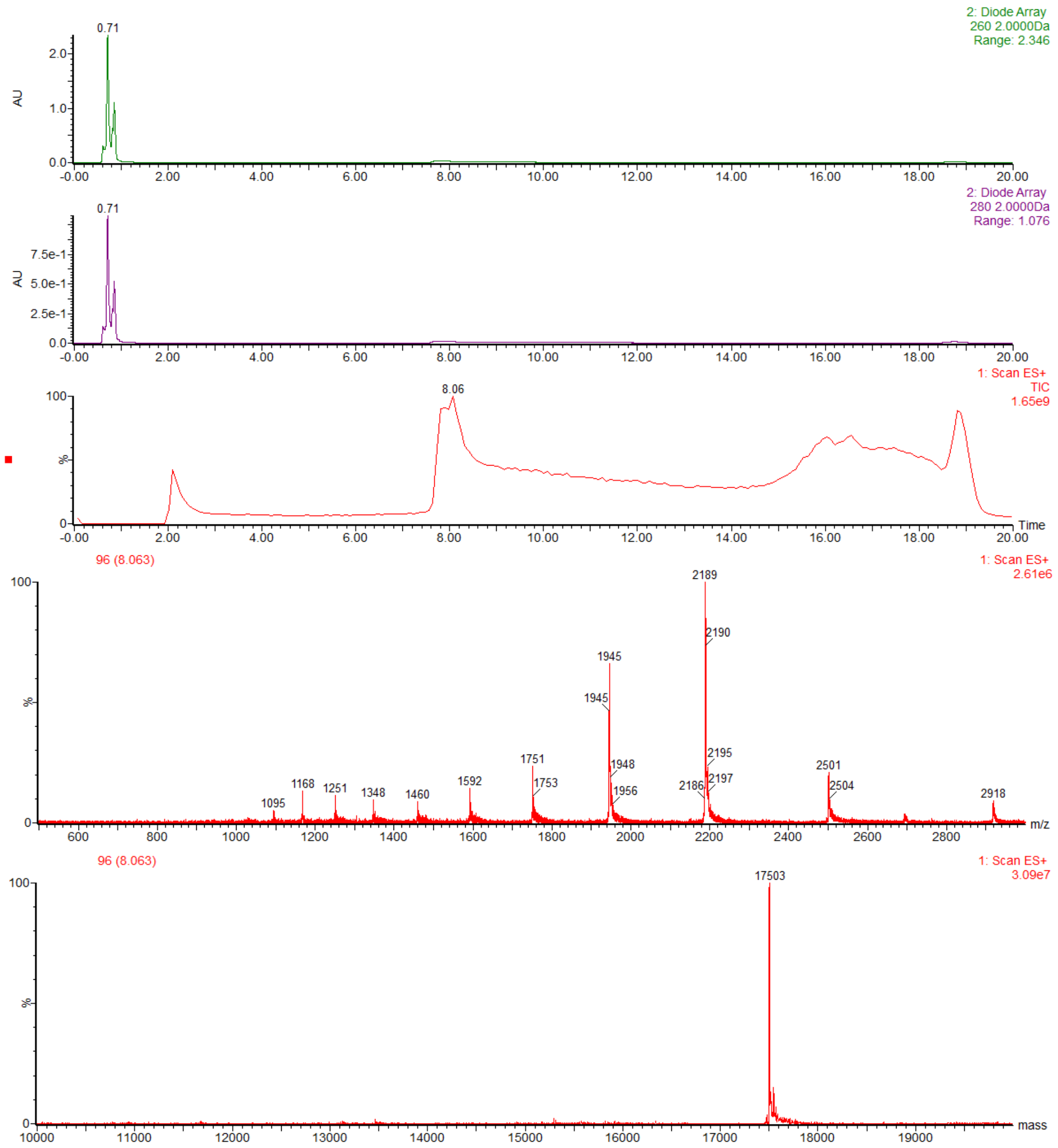


**Figure S34.** LC-MS analysis of the reaction mixture from the preparation of the **594Nup98 G85C-ss11-mer** conjugate with 2 mM ammonium acetate as the LC-MS mobile phase additive.

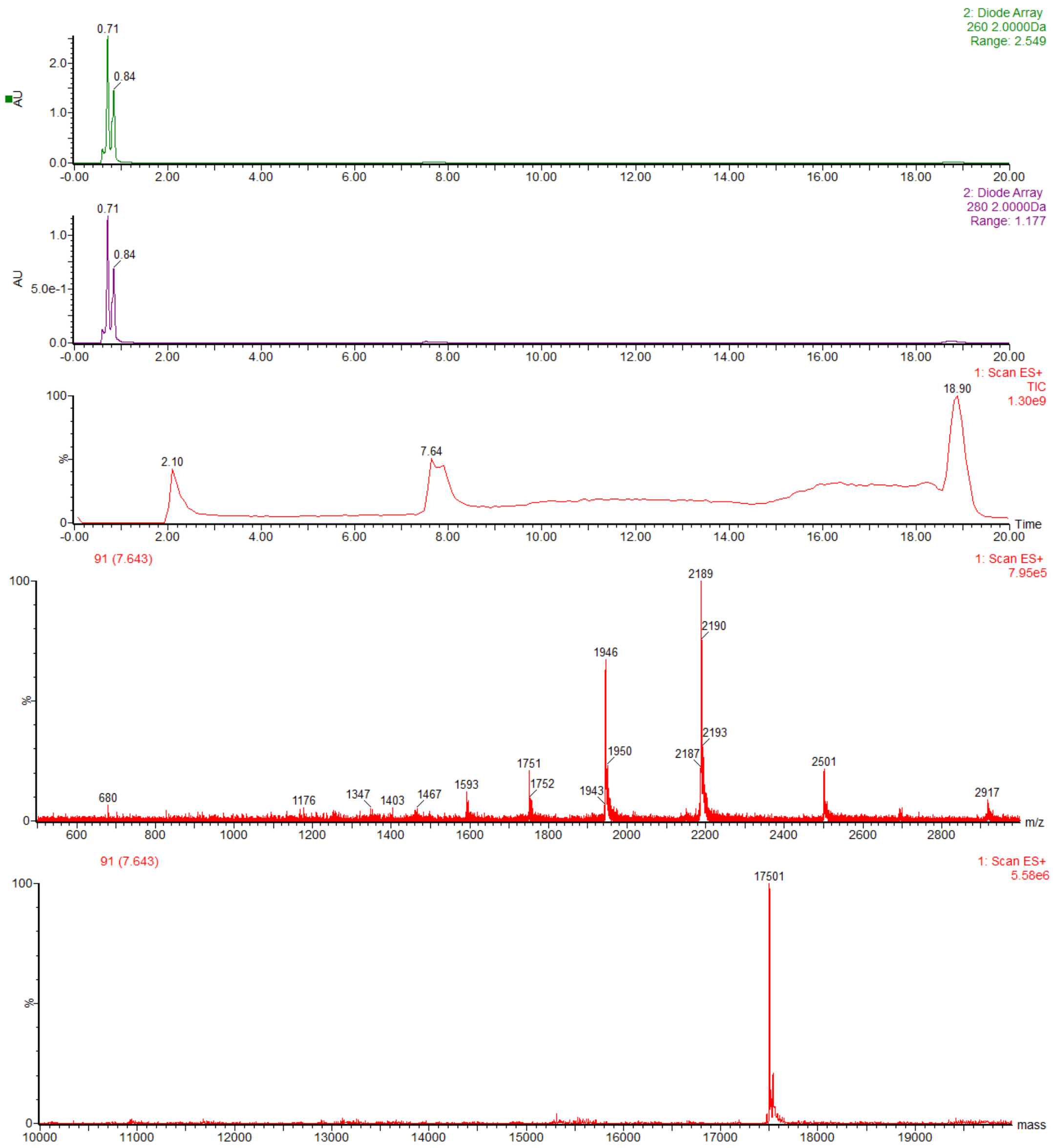


**Figure S35.** LC-MS analysis of the reaction mixture from the preparation of the **594Nup98 G85C-ss11-mer** conjugate with 1 mM ammonium acetate as the LC-MS mobile phase additive.

Use of ammonium formate as the LC-MS additive influenced the ionization of the sample, but the deconvolution and reconstruction of the total mass ion gave similar results to the ones obtained with ammonium acetate as the LC-MS additive. However, the change in the signal of the UV trace is noticeable.

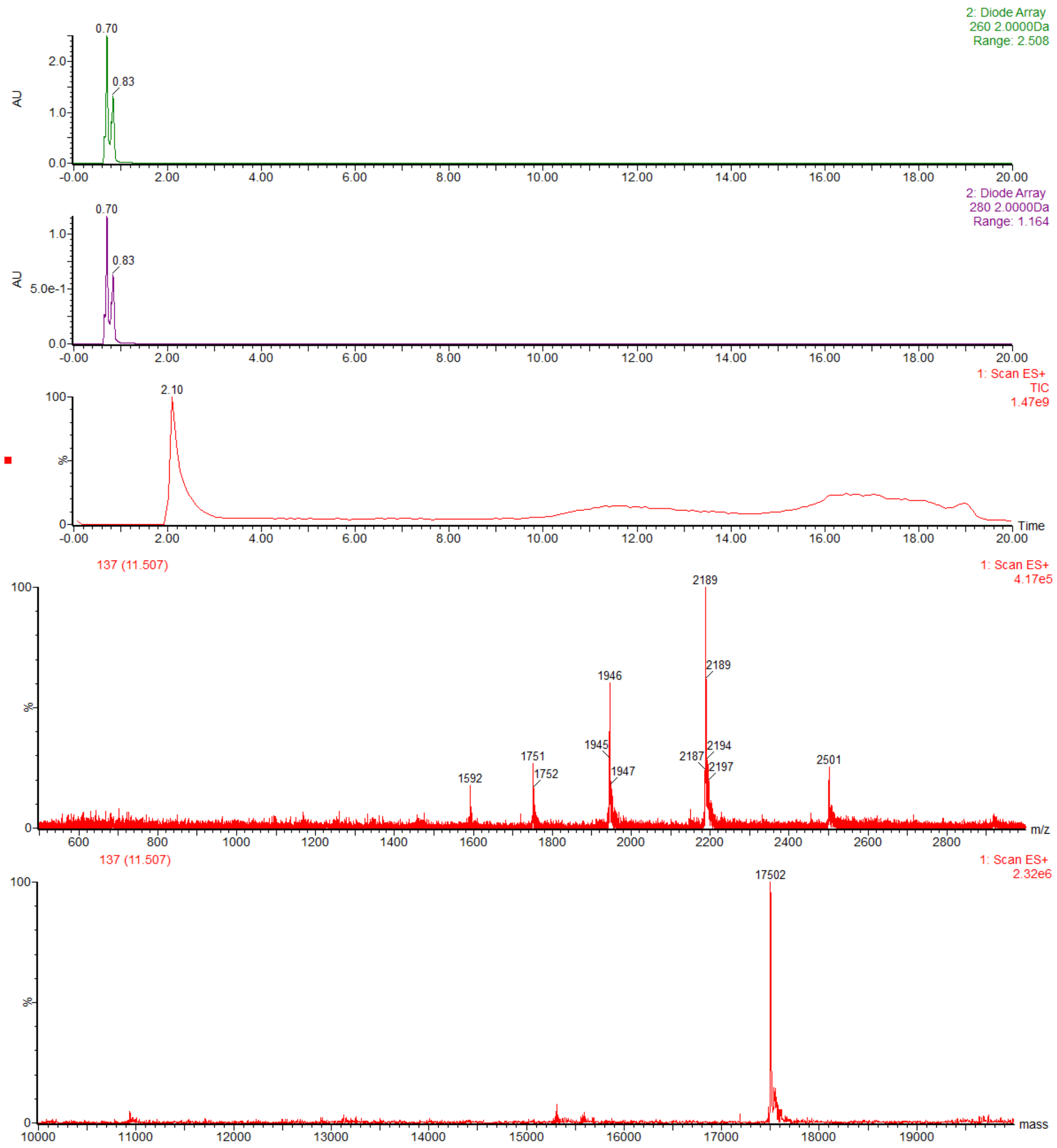


**Figure S36.** LC-MS analysis of the reaction mixture from the preparation of the **594Nup98 G85C-ss11-mer** conjugate with 1 mM ammonium formate as the LC-MS mobile phase additive.

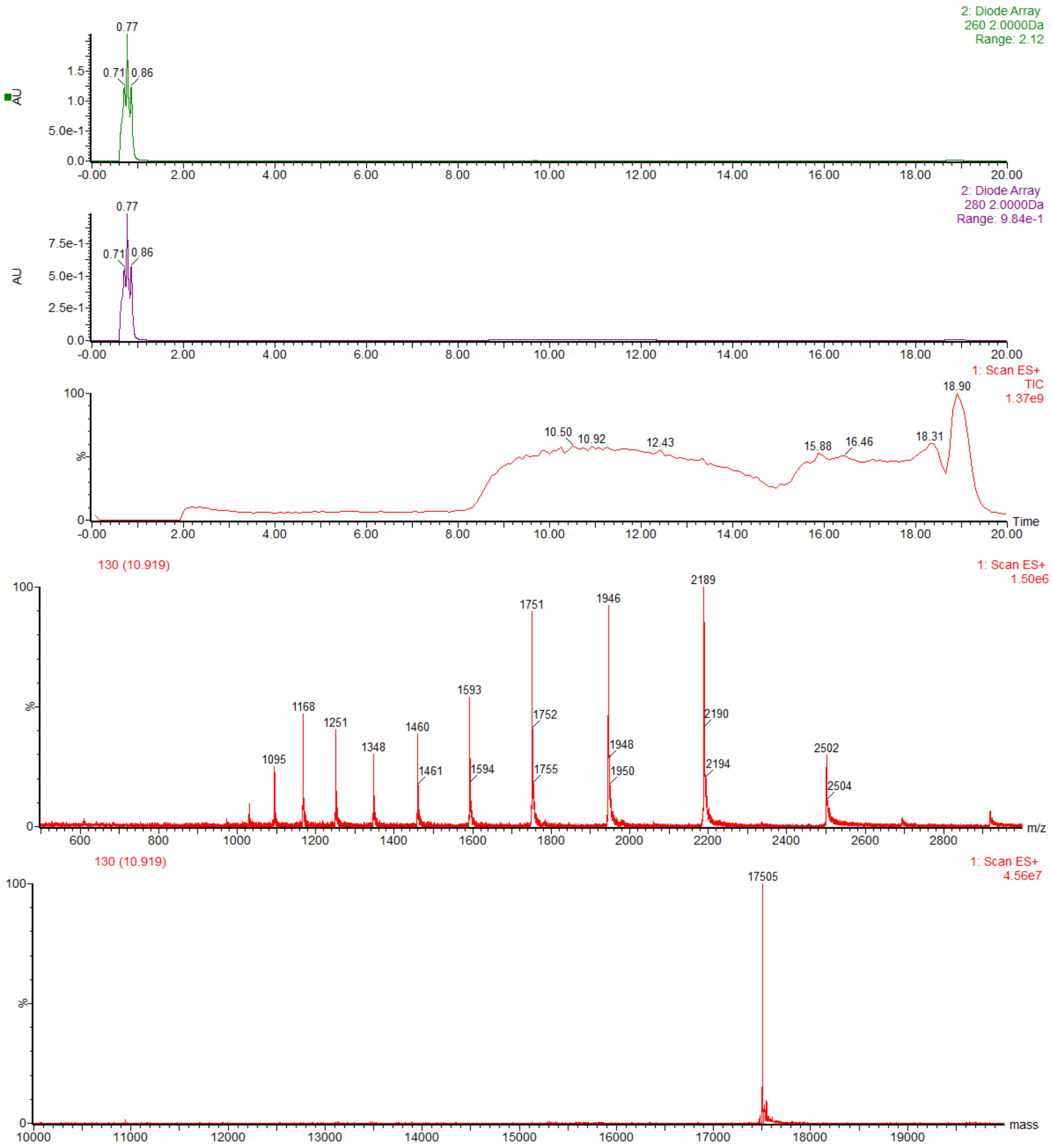


**Figure S37.** LC-MS analysis of the reaction mixture from the preparation of the **594Nup98 G85C-ss11-mer** conjugate with 10 mM ammonium formate as the LC-MS mobile phase additive.

Therefore, 1–10 mM ammonium acetate was chosen as the LC-MS additive for the optimized LC-MS analysis and characterization of prepared DNA-protein conjugates. The applicability and accuracy of the optimized LC-MS method was then tested on other three 11-mer-conjugates of anti-nucleoporin antibody variants.

In addition to the LC-MS additive, other MS parameters influencing mainly the sample ionization were fine-tuned as well. Although ammonium acetate as the LC-MS additive is suitable for the use in both positive and negative ESI MS modes,[10] performed tests have revealed that prepared DNA-protein conjugates get efficiently ionized only using positive ion mode under the conditions with ammonium acetate as the LC-MS additive. Furthermore, the effect of the sample cone voltage as well as the capillary voltage on the sample ionization was screened in the range of 20–40 V and 2–3.5 kV, respectively. 30 V for the cone voltage and 3.0 kV for the capillary voltage were found to be optimal for the ionization of DNA-protein samples on a single quadrupole SQD 2 mass detector from Waters® (see **Protein and oligonucleotide LC-MS** for details).

Preparation of **576Nup98 A75C-ss11-mer**, **427Nup93 C4-ss11-mer** and **443Nup98 C4-ss11-mer** conjugates for the tests of the optimized LC-MS method.

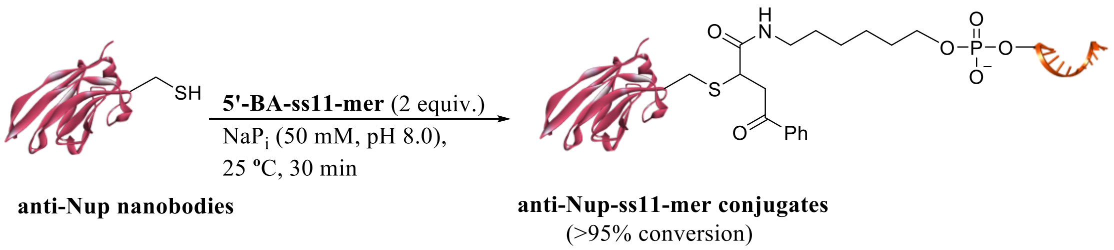


In an Eppendorf tube, the reaction mixture consisting of the **anti-Nup nanobody** (20 µM final concentration, 1 equiv.) and **5'-BA-ss11-mer** (40 µM final concentration, 2 equiv.) in 15 µL of 50 mM NaP$_i$ pH 8.0 was vortexed and shaken at 25 ºC for 30 min. 5 µL aliquots of reaction mixtures were then analyzed by LC-MS and complete conversion of starting materials to desired **576Nup98 A75C-ss11-mer** (calculated: 17483 Da; observed: 17486 Da), **427Nup93 C4-ss11-mer** (calculated: 16923 Da; observed: 16928 Da) and **443Nup98 C4-ss11-mer** (calculated: 18013 Da; observed: 18017 Da) conjugate products were obtained.

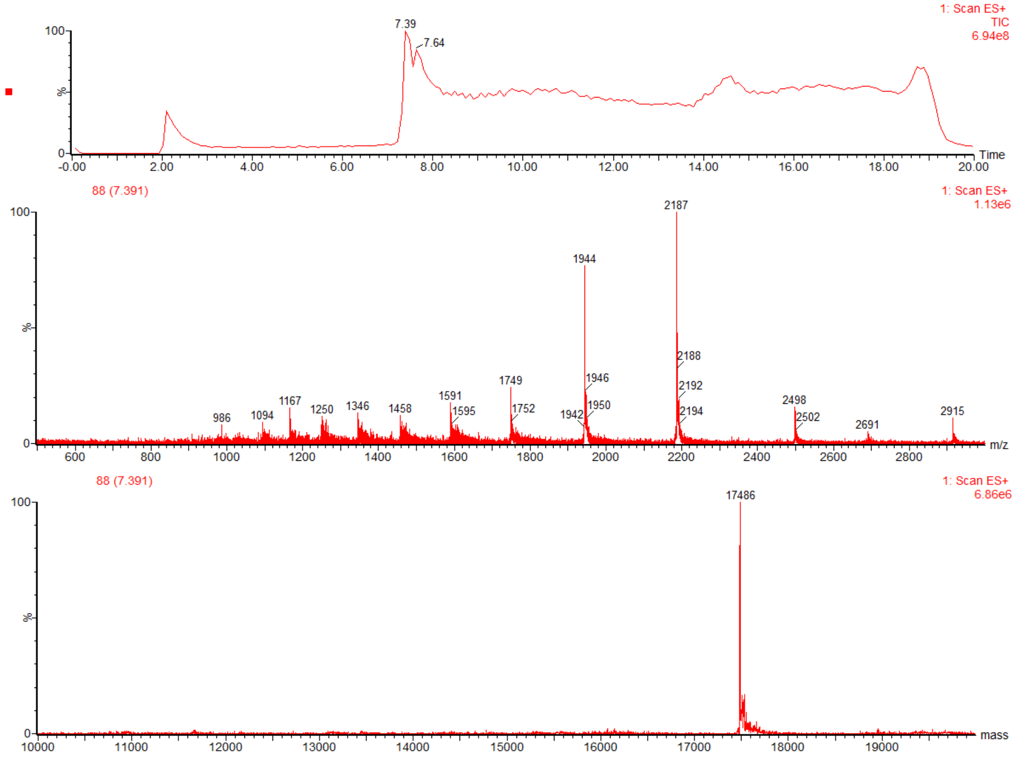


**Figure S38.** LC-MS analysis of the reaction mixture from the preparation of the **576Nup98 A75C-ss11-mer** conjugate.

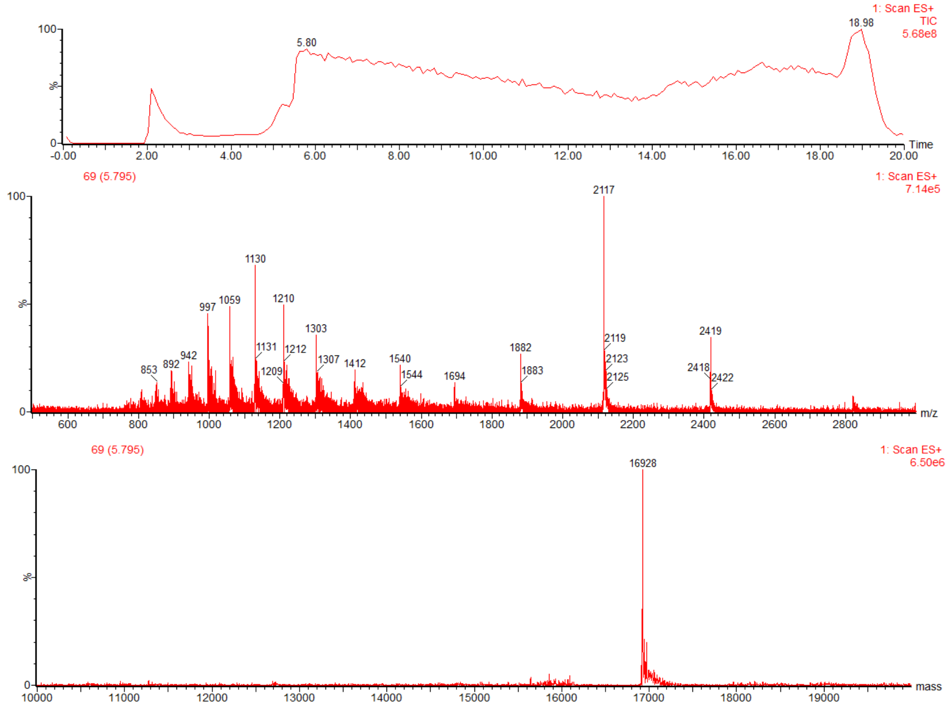


**Figure S39.** LC-MS analysis of the reaction mixture from the preparation of the **427Nup93 C4-ss11-mer** conjugate.

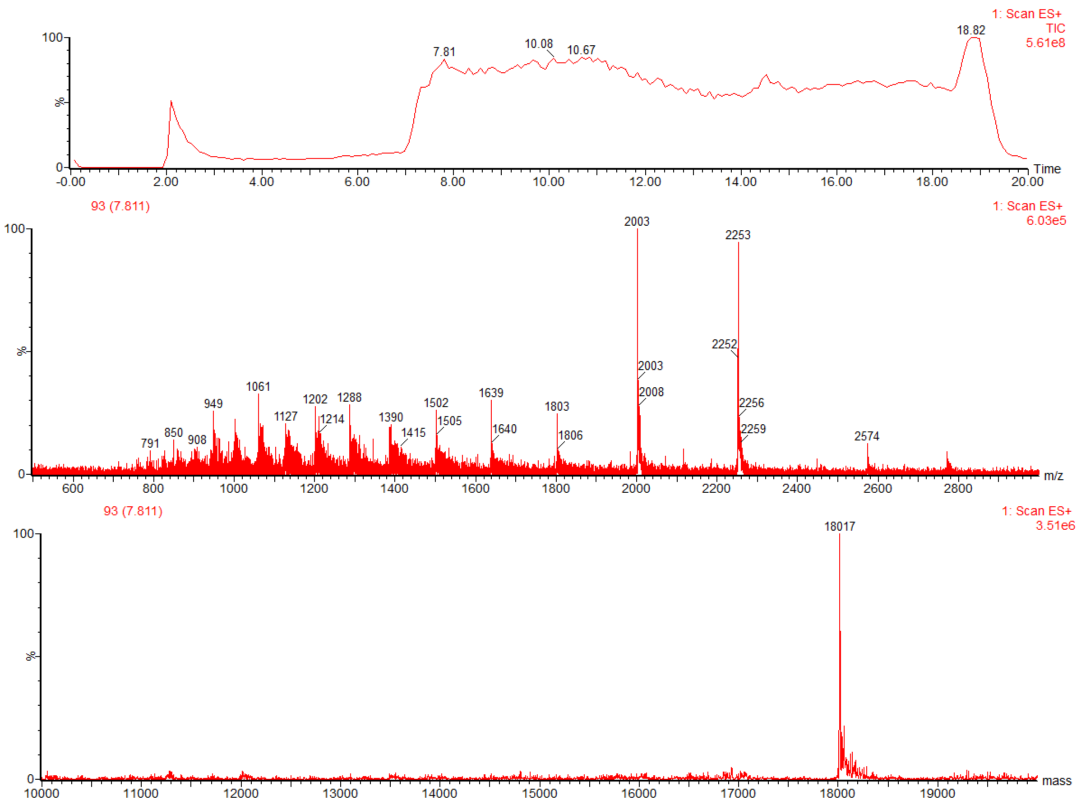


**Figure S40.** LC-MS analysis of the reaction mixture from the preparation of the **443Nup98 C4-ss11-mer** conjugate.

In order to save in some cases expensive ON material, to work out the correct stoichiometric ratio of the protein and BA-labelled ONs required for the bioconjugation reaction, amount of the BA-labelled ON can be optimized in a test protein modification reaction using the BA-*N*-Et reagent **3**.[4,11] Amount of the BA-labelled ON necessary for the complete conversion of the starting protein material to the desired bioconjugate then directly corresponds to the amount of the small molecule linker **3** needed for the complete protein modification.

## DNA-protein and DNA-antibody conjugation

### Ubiquitin K63C-ss11-mer

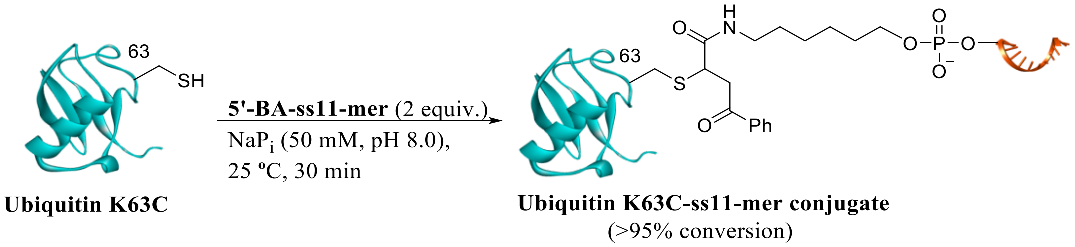


In an Eppendorf tube, the reaction mixture consisting of **Ubiquitin K63C** (20 µM final concentration, 1 equiv.) and **5'-BA-ss11-mer** (40 µM final concentration, 2 equiv.) in 20 µL of 50 mM NaP$_i$ pH 8.0 was vortexed and shaken at 25 ºC for 30 min. 5 µL aliquot of the reaction mixture was then analyzed by LC-MS and complete conversion of the starting material to the desired **Ubiquitin K63C-ss11-mer** conjugate product was obtained (calculated: 12194 Da; observed: 12198 Da).

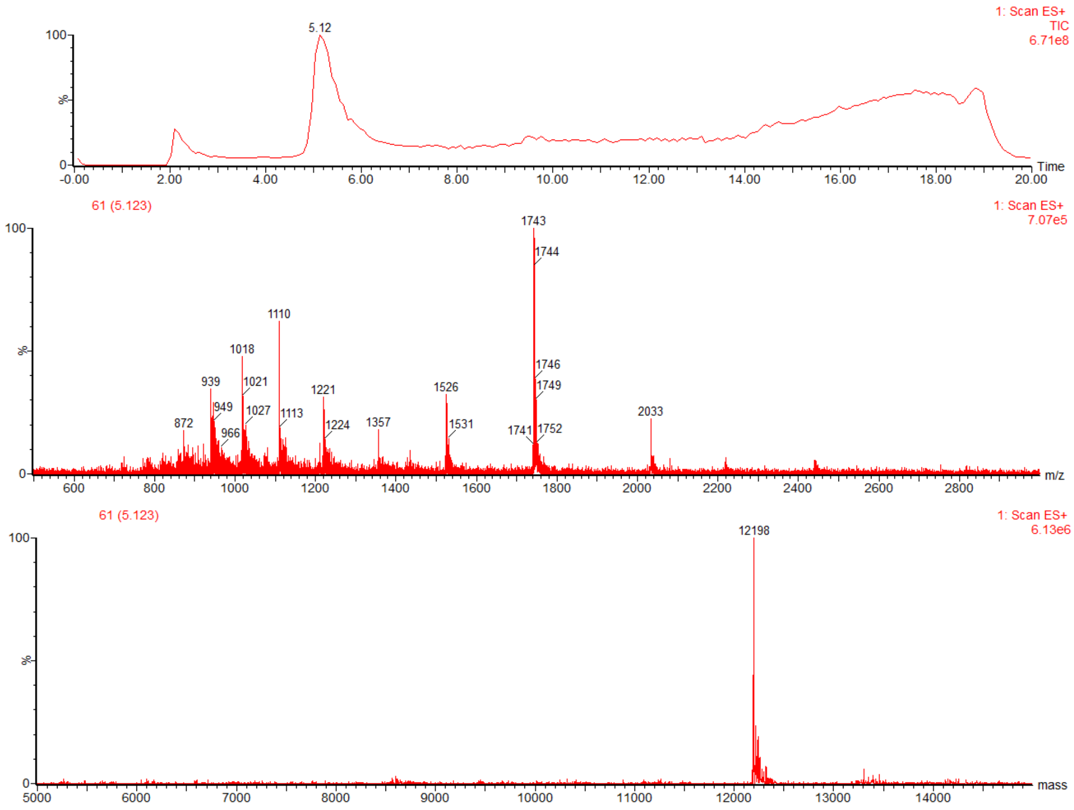


**Figure S41.** LC-MS analysis of the reaction mixture from the preparation of the **Ubiquitin K63C-ss11-mer** conjugate.

Control reaction between **Ubiquitin K63C** and **5'-NH$_2$-ss11-mer**

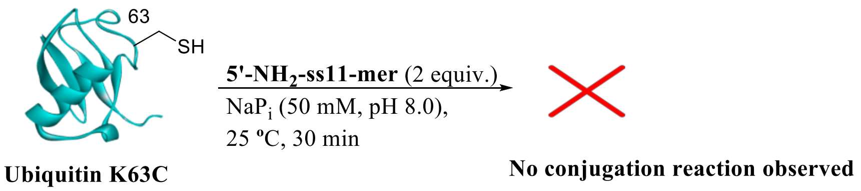


In an Eppendorf tube, the reaction mixture consisting of **Ubiquitin K63C** (20 µM final concentration, 1 equiv.) and **5'-NH$_2$-ss11-mer** (40 µM final concentration, 2 equiv.) in 20 µL of 50 mM NaP$_i$ pH 8.0 was vortexed and shaken at 25 ºC for 30 min. 5 µL aliquot of the reaction mixture was then analyzed by LC-MS and only the starting material **Ubiquitin K63C** without any modification was obtained (calculated: 8567 Da; observed: 8569 Da).

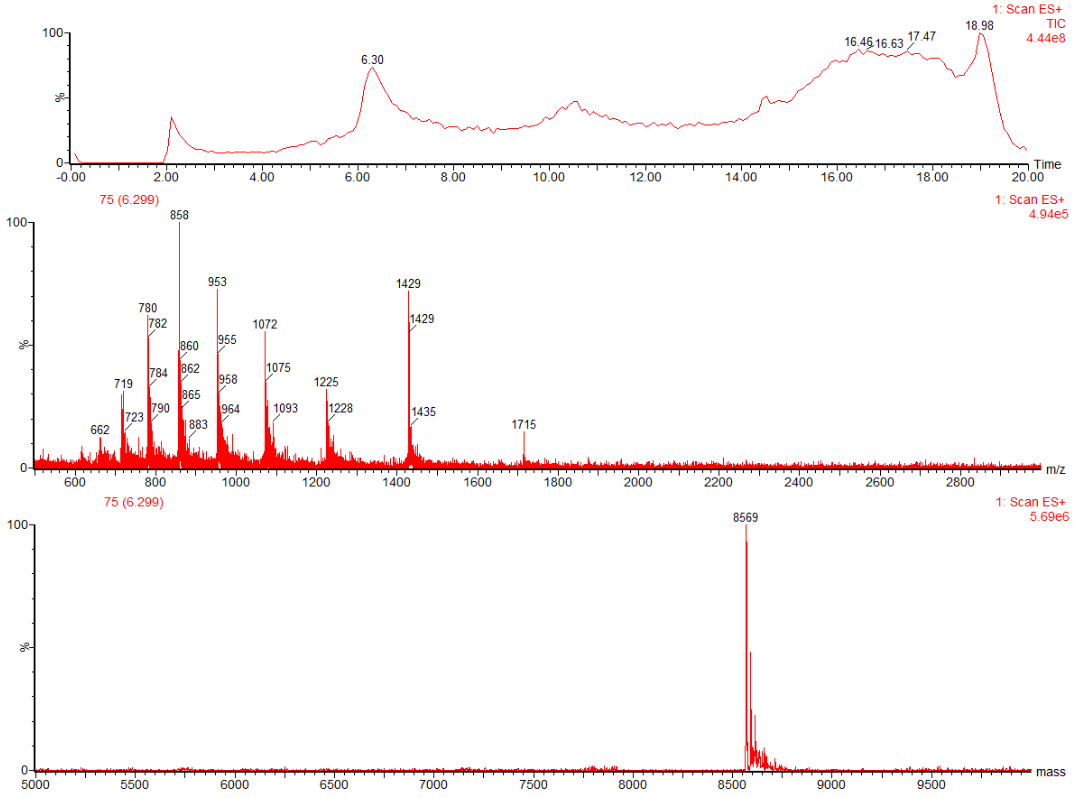


**Figure S42.** LC-MS analysis of the reaction mixture from the reaction between **Ubiquitin K63C** and **5'-NH$_2$-ss11-mer.**

**C2Am-C95-ss11-mer**

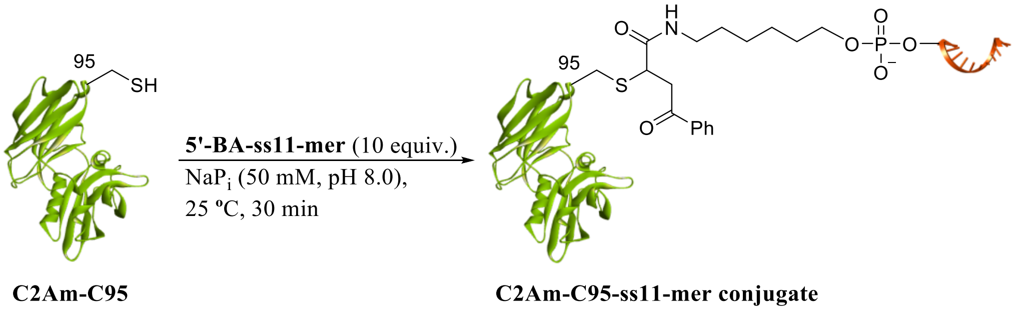


In an Eppendorf tube, the reaction mixture consisting of freshly reduced **C2Am-C95** (18 µM final concentration, 1 equiv.) and **5'-BA-ss11-mer** (180 µM final concentration, 10 equiv.) in 22 µL of 50 mM NaP$_i$ pH 8.0 was vortexed and shaken at 25 ºC for 30 min. 5 µL aliquot of the reaction mixture was then analyzed by LC-MS and complete conversion of the starting material to the desired **C2Am-C95-ss11-mer** conjugate product was obtained (calculated: 19849 Da; observed: 19855 Da).

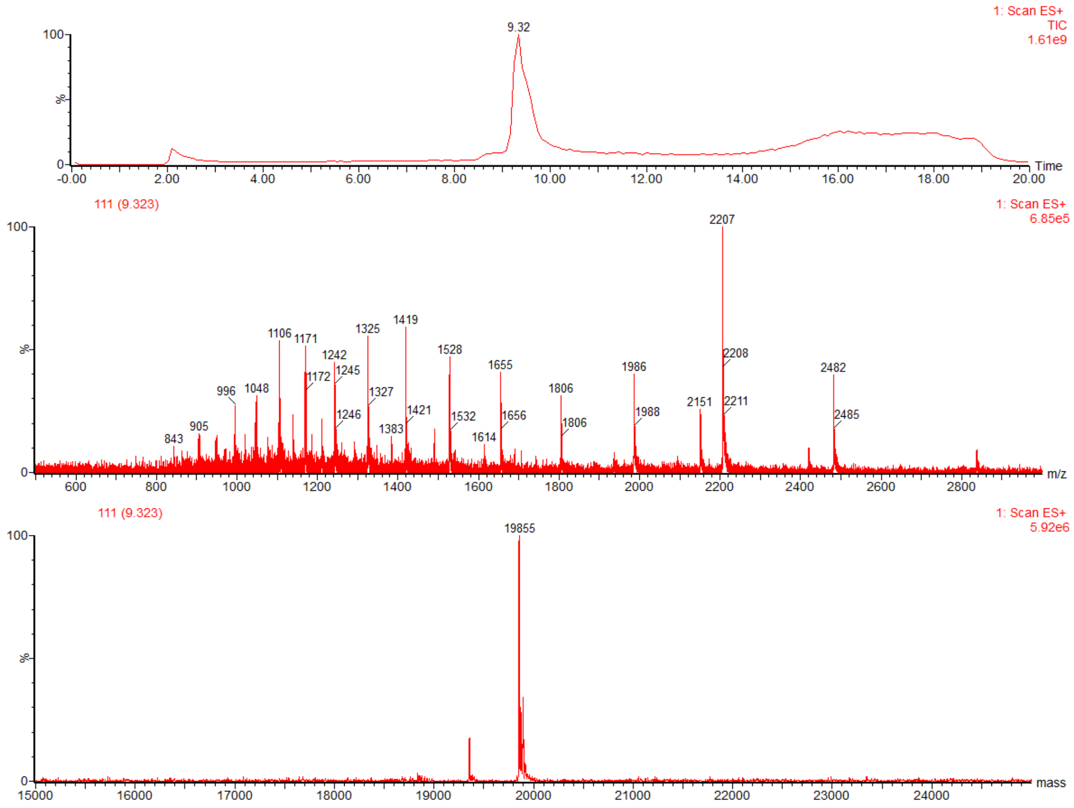


**Figure S43.** LC-MS analysis of the reaction mixture from the preparation of the **C2Am-C95-ss11-mer** conjugate.

## Annexin V-C315-ss11-mer

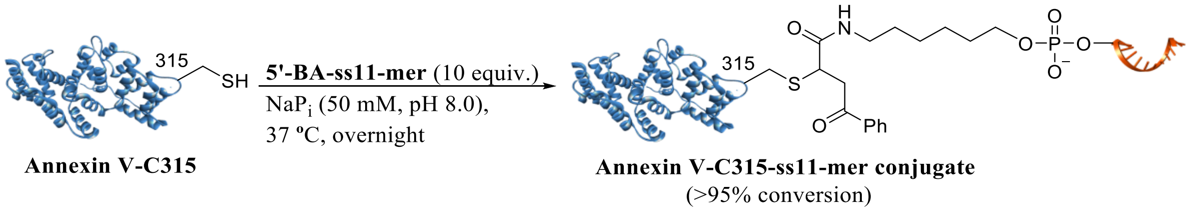


In an Eppendorf tube, the reaction mixture consisting of **Annexin V-C315** (20 µM final concentration, 1 equiv.) and **5'-BA-ss11-mer** (200 µM final concentration, 10 equiv.) in 30 µL of 50 mM NaP$_i$ pH 8.0 was vortexed and shaken at 37 ºC, overnight. 5 µL aliquot of the reaction mixture was then analyzed by LC-MS and complete conversion of the starting material to the desired **Annexin V-C315-ss11-mer** conjugate product was obtained (calculated: 39432 Da; observed: 39442 Da).

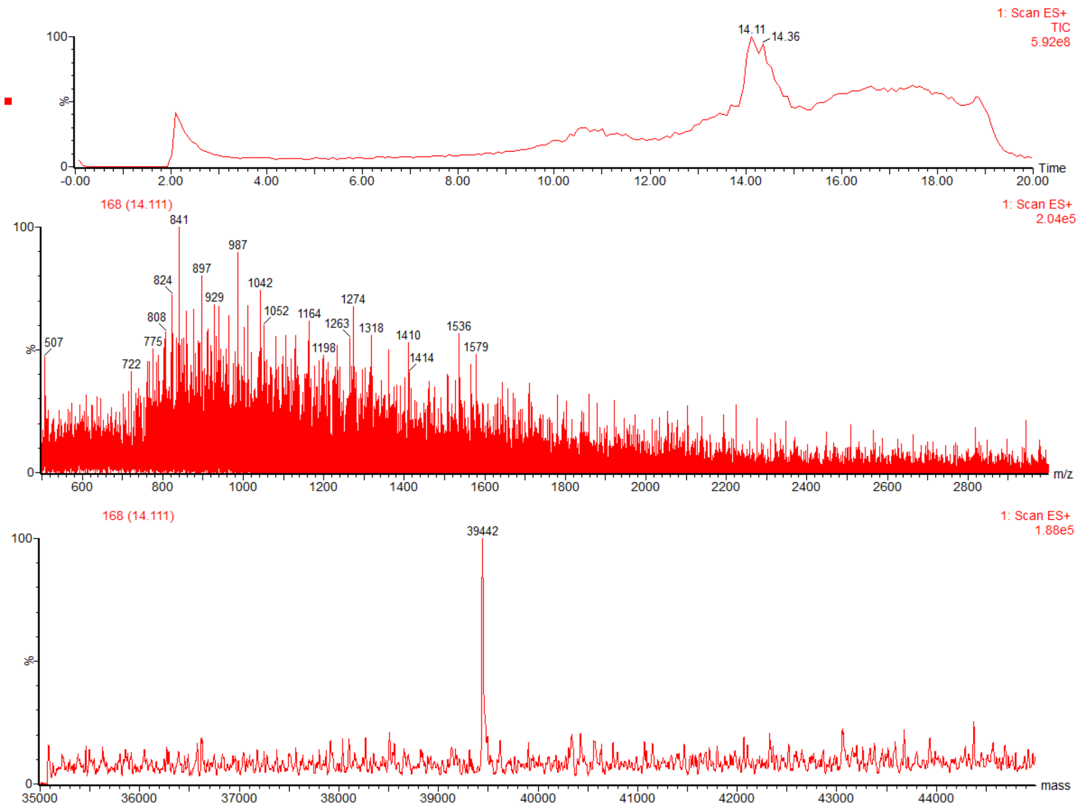


**Figure S44.** LC-MS analysis of the reaction mixture from the preparation of the **Annexin V-C315-ss11-mer** conjugate.

## HSA-C34-ss11-mer

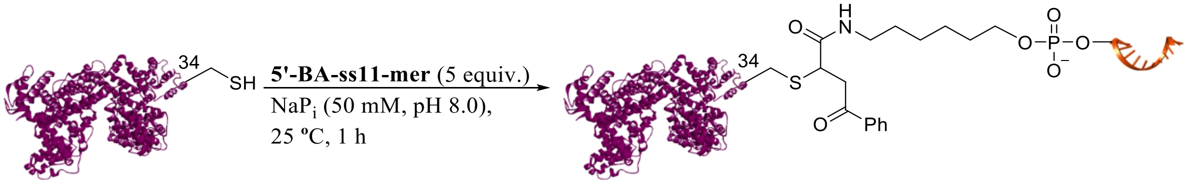


**HSA-C34** **HSA-C34-ss11-mer conjugate** (>95% conversion)

In an Eppendorf tube, the reaction mixture consisting of **HSA-C34** (20 µM final concentration, 1 equiv.) and **5'-BA-ss11-mer** (100 µM final concentration, 5 equiv.) in 20 µL of 50 mM $NaP_i$ pH 8.0 was vortexed and shaken at 25 ºC for 1 h. 5 µL aliquot of the reaction mixture was then analyzed by LC-MS and complete conversion of the starting material to the desired **HSA-C34-ss11-mer** conjugate product was obtained (calculated: 70067 Da; observed: 70087 Da).

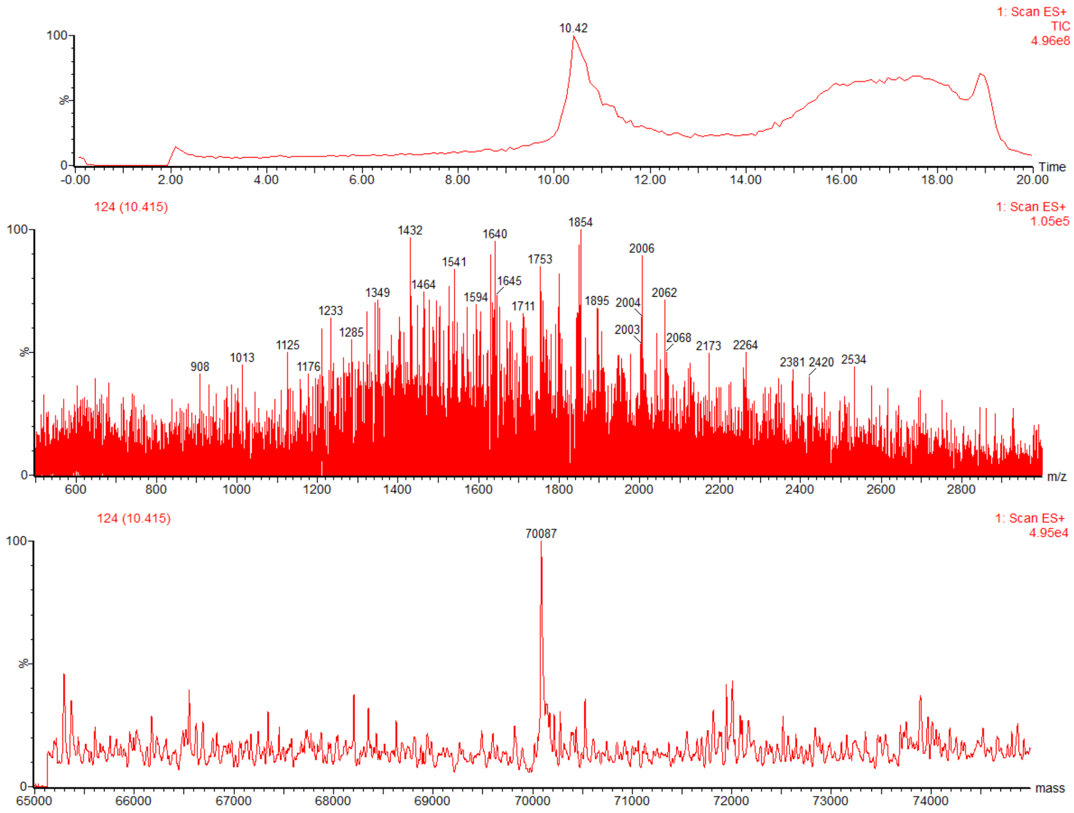


**Figure S45.** LC-MS analysis of the reaction mixture from the preparation of the **HSA-C34-ss11-mer** conjugate.

**C2Am-C95-ss25-mer**

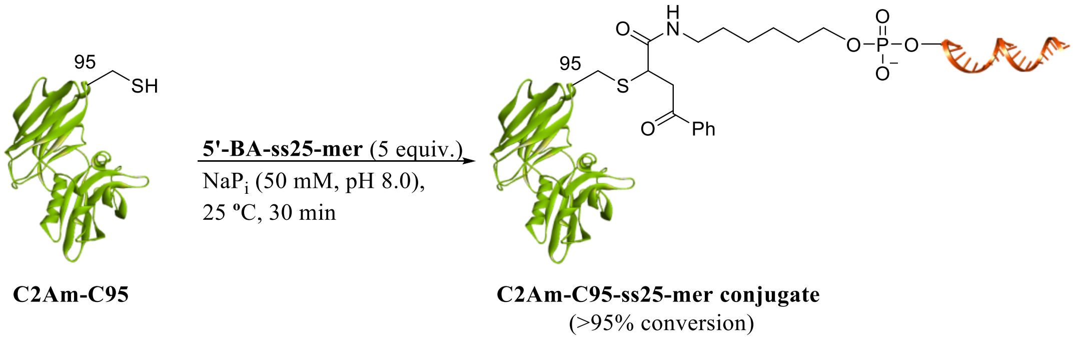


In an Eppendorf tube, the reaction mixture consisting of the freshly reduced **C2Am-C95** (13 µM final concentration, 1 equiv.) and **5'-BA-ss25-mer** (180 µM final concentration, 5 equiv.) in 23 µL of 50 mM $NaP_i$ pH 8.0 was vortexed and shaken at 25 ºC for 30 min. 5 µL aliquot of the reaction mixture was then analyzed by LC-MS and complete conversion of the starting material to the desired **C2Am-C95-ss25-mer** conjugate product was obtained (calculated: 24220 Da; observed: 24227 Da).

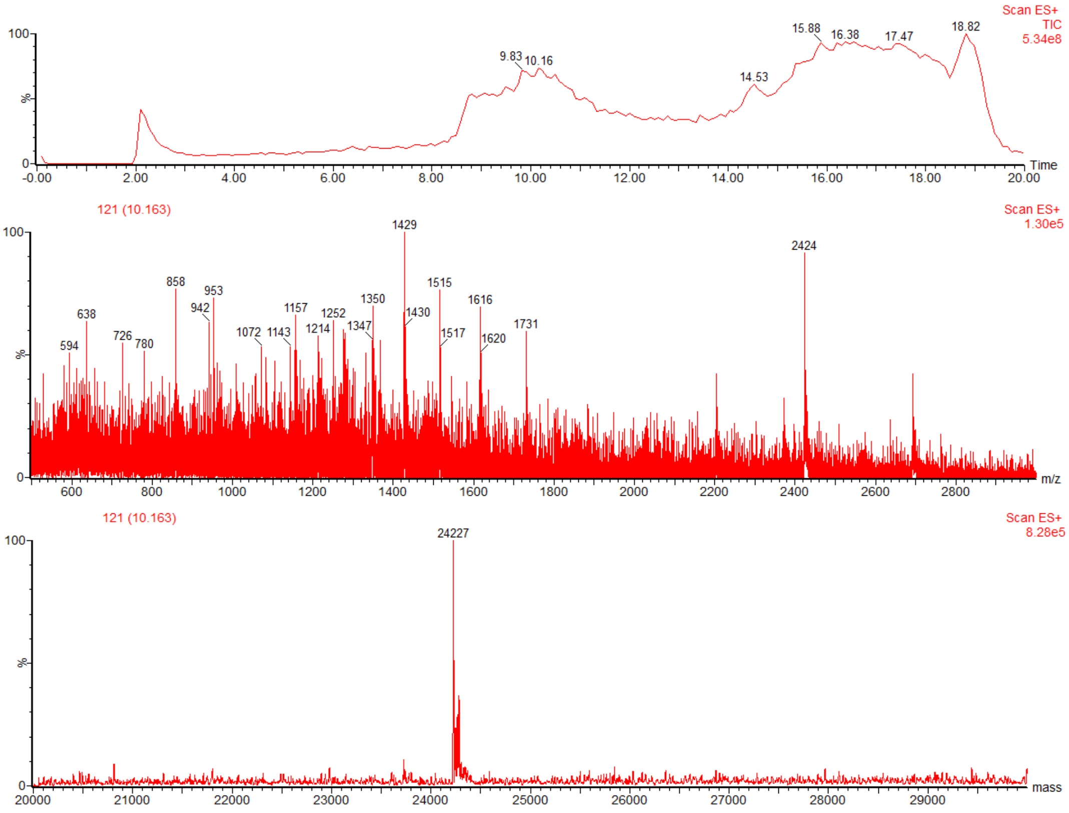


**Figure S46.** LC-MS analysis of the reaction mixture from the preparation of the **C2Am-C95-ss25-mer** conjugate.

**C2Am-C95-ss50-mer**

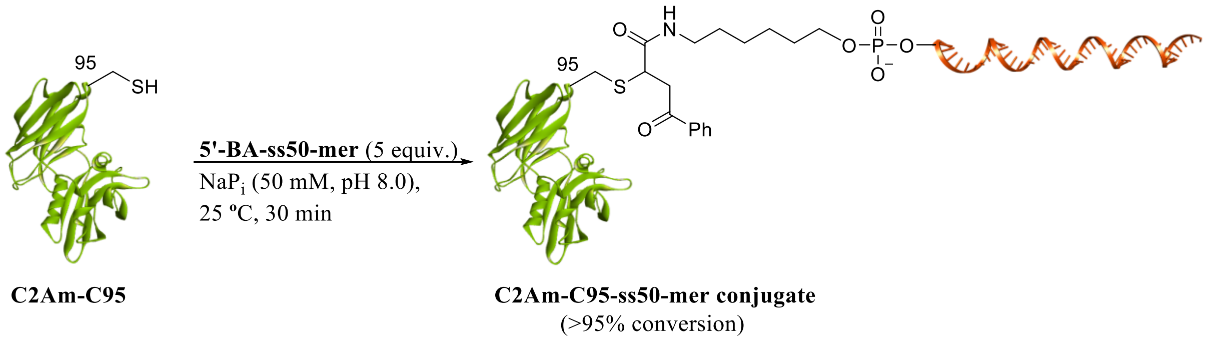


In an Eppendorf tube, the reaction mixture consisting of the freshly reduced **C2Am-C95** (13 µM final concentration, 1 equiv.) and **5'-BA-ss50-mer** (180 µM final concentration, 5 equiv.) in 23 µL of 50 mM $NaP_i$ pH 8.0 was vortexed and shaken at 25 ºC for 30 min. 5 µL aliquot of the reaction mixture was then analyzed by LC-MS and complete conversion of the starting material to the desired **C2Am-C95-ss50-mer** conjugate product was obtained (calculated: 31905 Da; observed: 31915 Da).

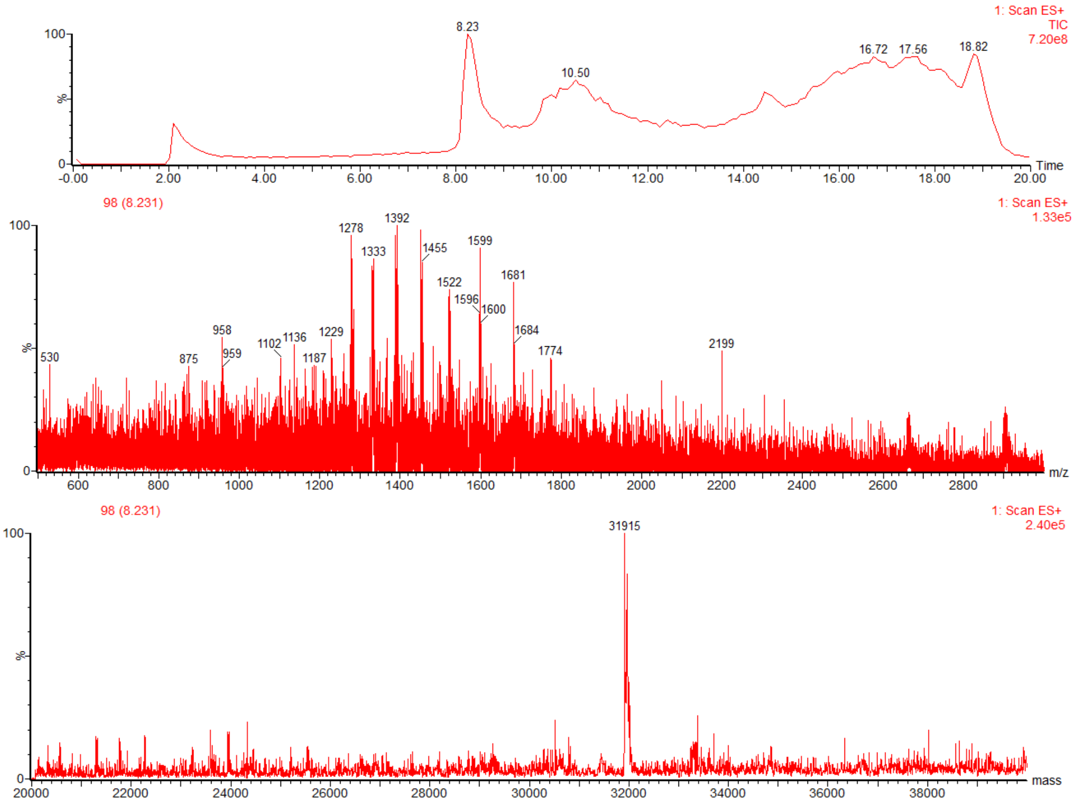


**Figure S47.** LC-MS analysis of the reaction mixture from the preparation of the **C2Am-C95-ss50-mer** conjugate.

## C2Am-C95-ds19-mer

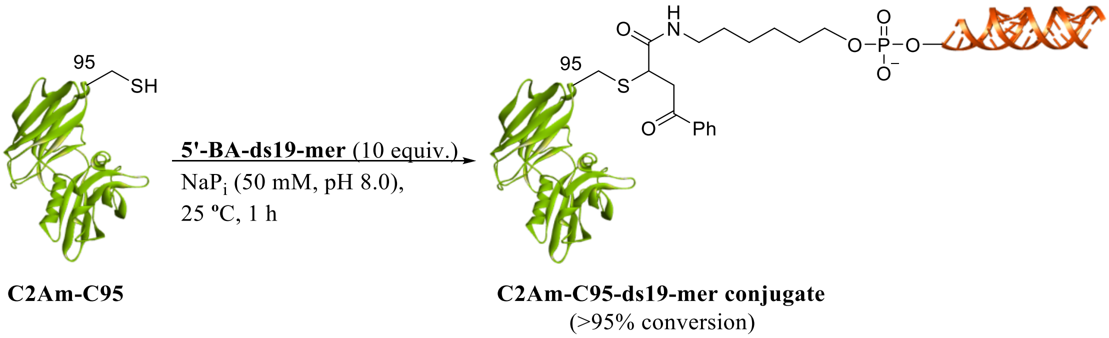


In an Eppendorf tube, the reaction mixture consisting of the freshly reduced **C2Am-C95** (15 µM final concentration, 1 equiv.) and **5'-BA-ss50-mer** (180 µM final concentration, 10 equiv.) in 26 µL of 50 mM NaP$_i$ pH 8.0 was vortexed and shaken at 25 ºC for 1 h. 5 µL aliquot of the reaction mixture was then analyzed by LC-MS and complete conversion of the starting material to the desired **C2Am-C95-ds19-mer** conjugate product was obtained (calculated with the sense strand: 22356 Da; observed: 22354 Da).

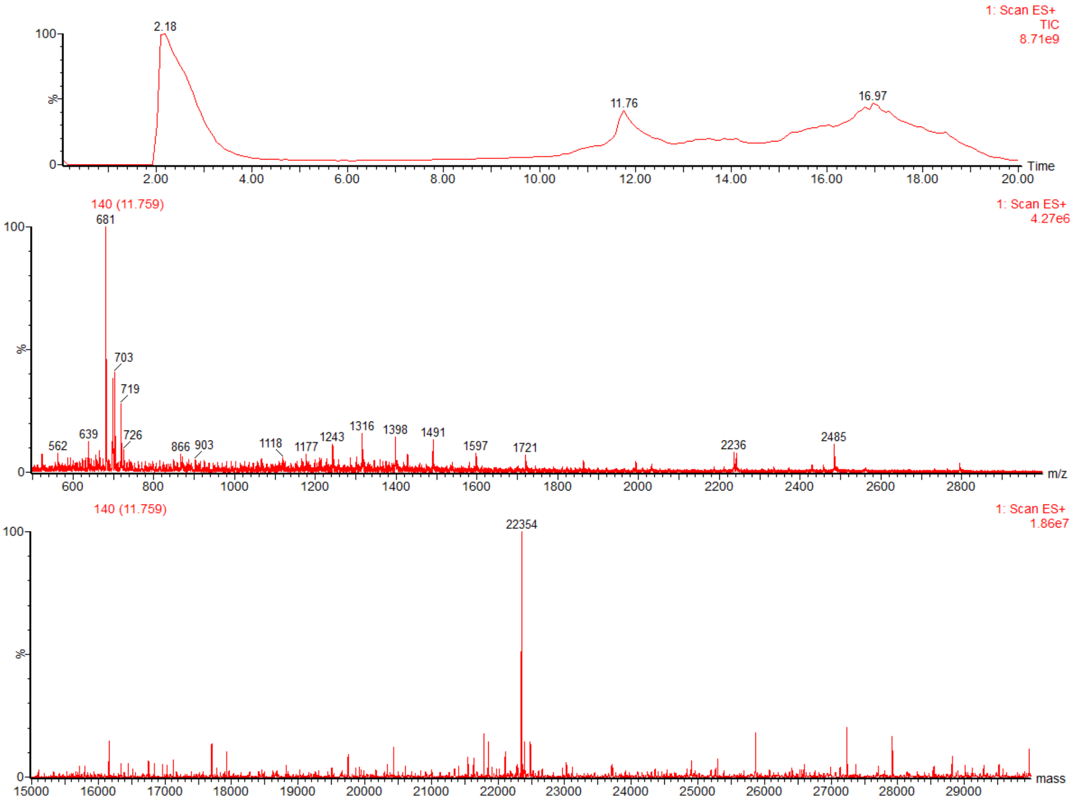


**Figure S48.** LC-MS analysis of the reaction mixture from the preparation of the **C2Am-C95-ds19-mer** conjugate.

## Thiomab LC-V205C-ss11-mer

## Thiomab LC-V205C-ss11-mer

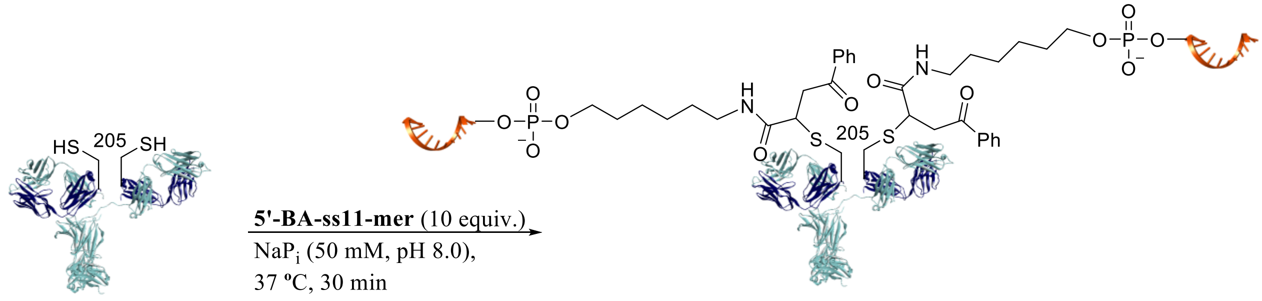


**Thiomab LC-V205C**

**Thiomab LC-V205C-ss11-mer conjugate**
(>95% conversion)

In an Eppendorf tube, the reaction mixture consisting of Cys-decapped **Thiomab LC-V205C** (10 µM final concentration, 1 equiv.) and **5'-BA-ss11-mer** (100 µM final concentration, 10 equiv.) in 20 µL of 50 mM NaP$_i$ pH 8.0 was vortexed and shaken at 37 ºC for 30 min. Unreacted ON was removed by Zeba™ Micro Spin Desalting Column 7K MWCO and antibody disufide bonds were reduced prior to LC-MS analysis according to the general pre-LC-MS reduction method. 5 µL aliquot of the reaction mixture was then analyzed by LC-MS and complete conversion of the starting material to the desired **Thiomab LC-V205C-ss11-mer** conjugate product was obtained (calculated LC: 27068 Da; observed LC: 27069 Da, no HC modification was observed).

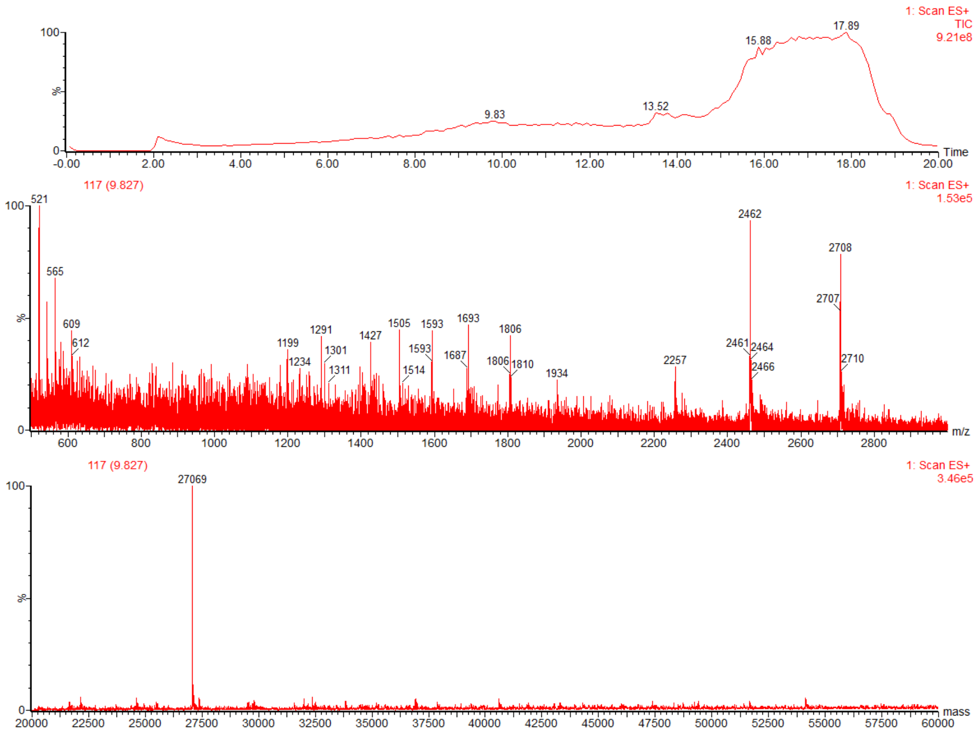


**Figure S49.** LC-MS analysis of the reaction mixture from the preparation of the **Thiomab LC-V205C-ss11-mer** conjugate.

## Gemtuzumab LC-V205C-ss11-mer

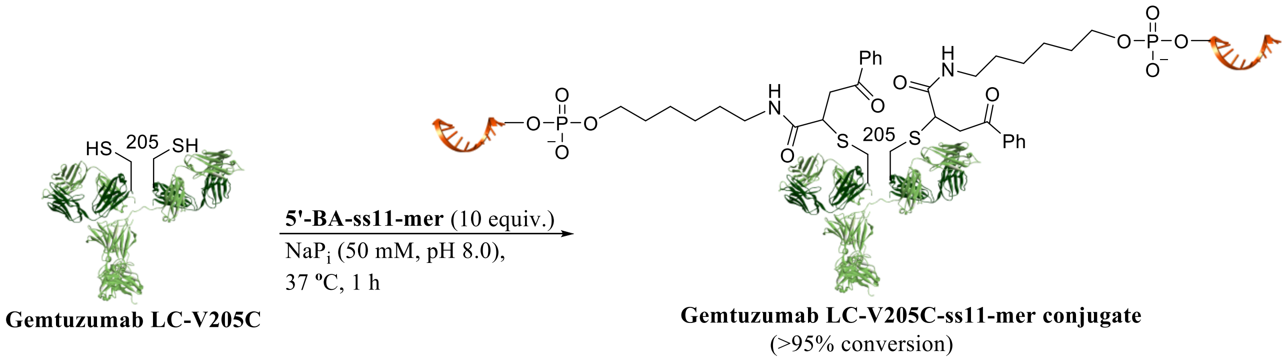


In an Eppendorf tube, the reaction mixture consisting of Cys-decapped **Gemtuzumab LC-V205C** (18 µM final concentration, 1 equiv.) and **5'-BA-ss11-mer** (180 µM final concentration, 10 equiv.) in 15 µL of 50 mM $NaP_i$ pH 8.0 was vortexed and shaken at 37 ºC for 1 h. Unreacted ON was removed by Zeba™ Micro Spin Desalting Column 7K MWCO and antibody disufide bonds were reduced prior to the LC-MS analysis according to the general pre-LC-MS reduction method. 5 µL aliquot of the reaction mixture was then analyzed by LC-MS and complete conversion of the starting material to the desired **Gemtuzumab LC-V205C-ss11-mer** conjugate product was obtained (calculated LC: 27452 Da; observed LC: 27451 Da, no HC modification observed).

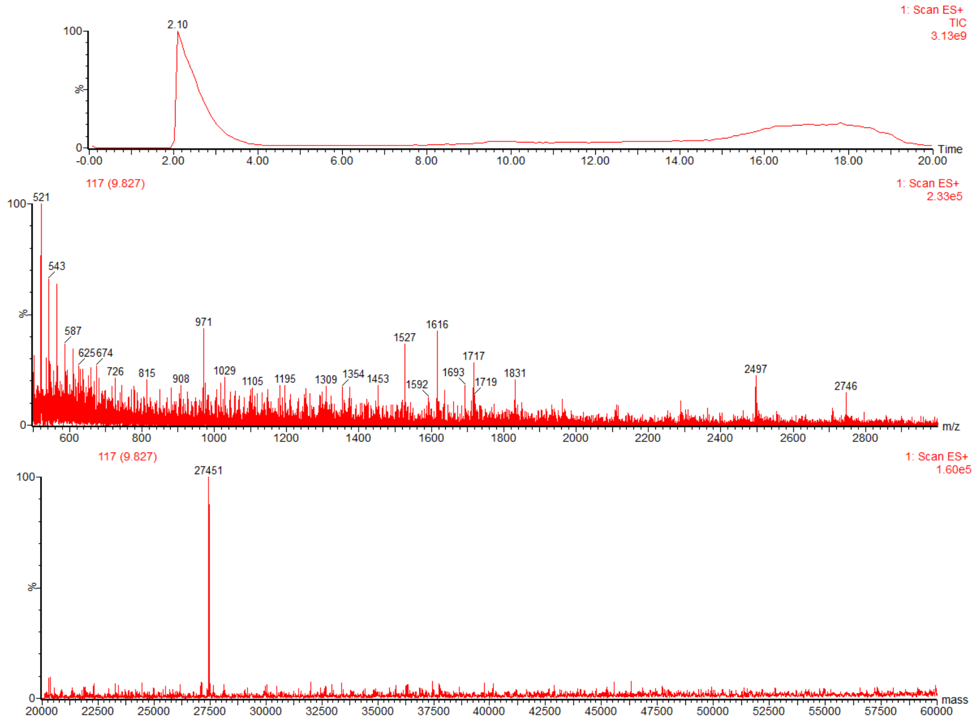


**Figure S50.** LC-MS analysis of the reaction mixture from the preparation of the **Gemtuzumab LC-V205C-ss11-mer** conjugate.

## Gemtuzumab i239C-ss11-mer

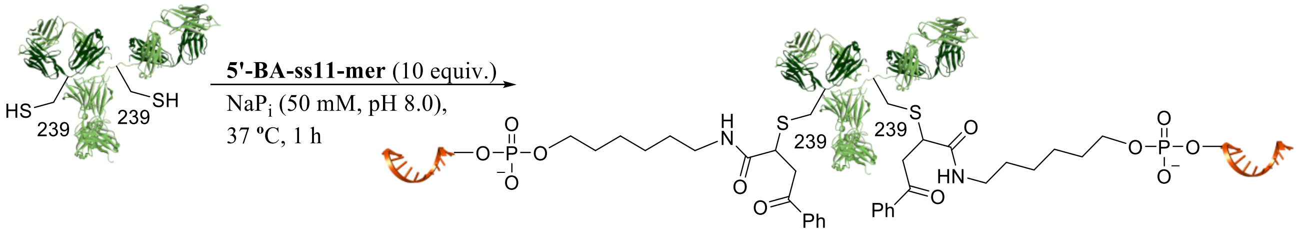


**Gemtuzumab i239C**

**Gemtuzumab i239C-ss11-mer conjugate**
(>95% conversion)

In an Eppendorf tube, the reaction mixture consisting of Cys-decapped **Gemtuzumab i239C** (20 µM final concentration, 1 equiv.) and **5'-BA-ss11-mer** (200 µM final concentration, 10 equiv.) in 12 µL of 50 mM NaP$_i$ pH 8.0 was vortexed and shaken at 37 ºC for 1 h. Unreacted ON was removed by Zeba™ Micro Spin Desalting Column 7K MWCO and antibody disufide bonds were reduced prior to the LC-MS analysis according to the general pre-LC-MS reduction method. 5 µL aliquot of the reaction mixture was then analyzed by LC-MS and complete conversion of the starting material to the desired **Gemtuzumab i239-ss11-mer** conjugate product was obtained (calculated HC: 54066 Da; observed HC: 54052 Da, no LC modification observed).

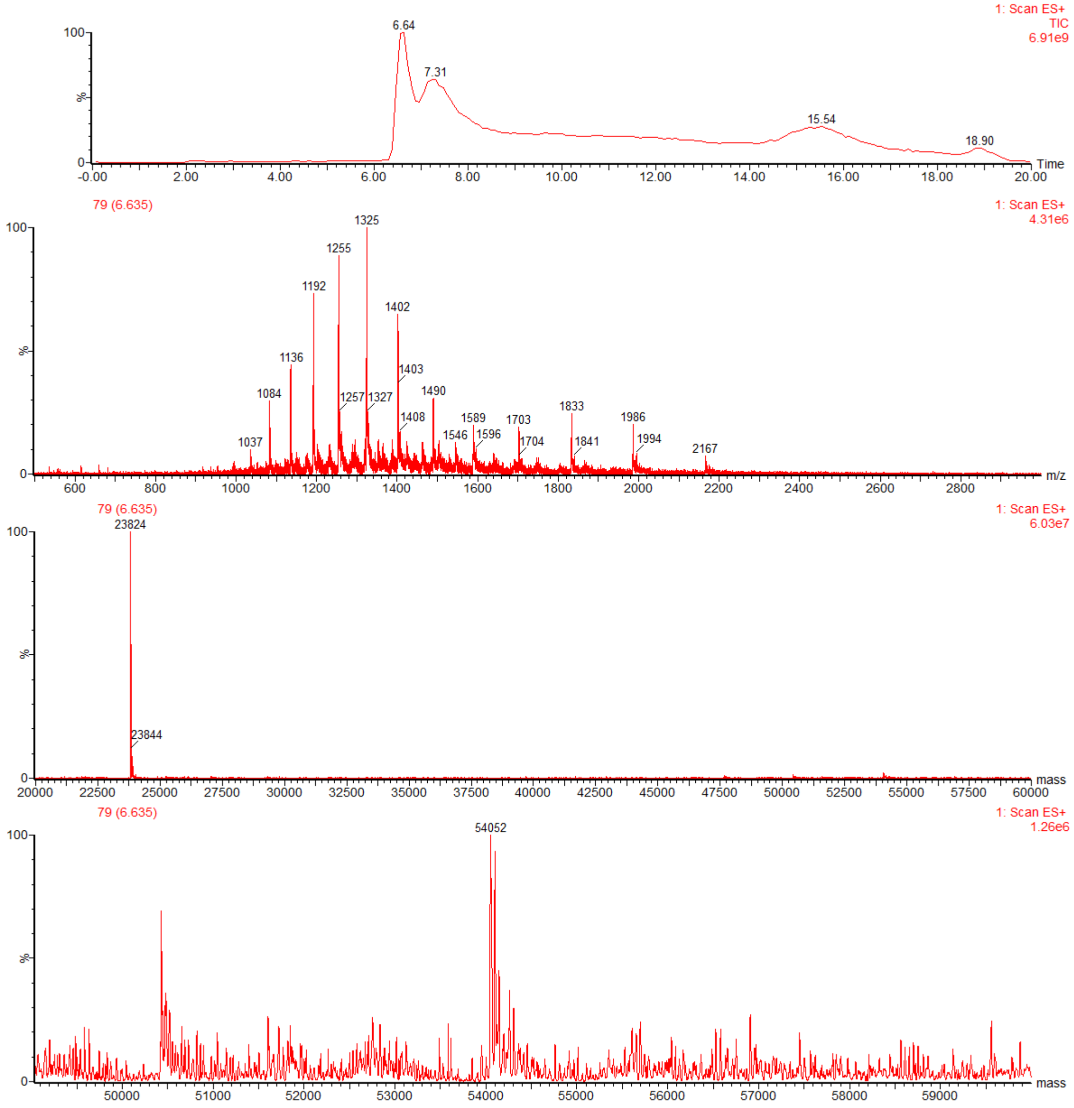


**Figure S51.** LC-MS analysis of the reaction mixture from the preparation of the **Gemtuzumab i239-ss11-mer** conjugate. Characterized by the protein LC-MS method using formic acid as the LC-MS additive.

**Gemtuzumab HC-S442C-ss11-mer**

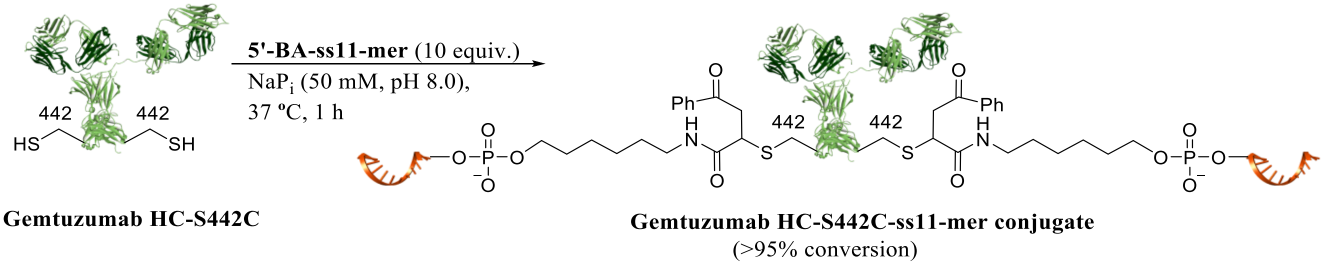


In an Eppendorf tube, the reaction mixture consisting of Cys-decapped **Gemtuzumab HC-S442C** (20 µM final concentration, 1 equiv.) and **5'-BA-ss11-mer** (200 µM final concentration, 10 equiv.) in 12 µL of 50 mM $NaP_i$ pH 8.0 was vortexed and shaken at 37 ºC for 1 h. Unreacted ON was removed by Zeba™ Micro Spin Desalting Column 7K MWCO and antibody disufide bonds were reduced prior to the LC-MS analysis according to the general pre-LC-MS reduction method. 5 µL aliquot of the reaction mixture was then analyzed by LC-MS and complete conversion of the starting material to the desired **Gemtuzumab HC-S442C-ss11-mer** conjugate product was obtained (calculated HC: 53979 Da; observed LC: 53968 Da, no LC modification observed).

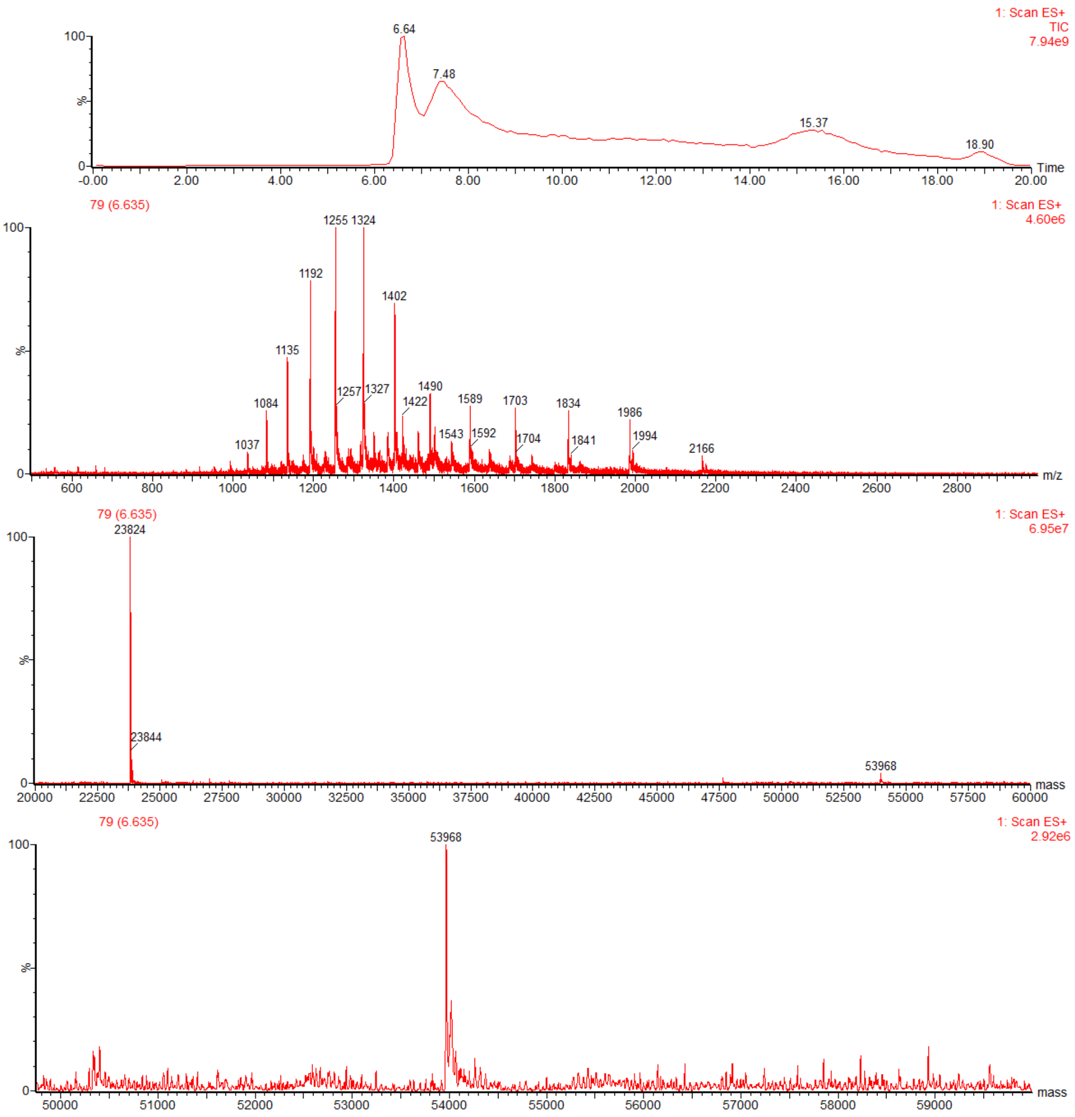


**Figure S52.** LC-MS analysis of the reaction mixture from the preparation of the **Gemtuzumab HC-S442C-ss11-mer** conjugate. Characterized by the protein LC-MS method using formic acid as the LC-MS additive.

Control reaction between **Trastuzumab** and **5'-BA-ss11-mer**

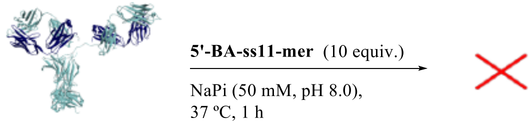


**Trastuzumab** **No conjugation reaction observed**

In an Eppendorf tube, the reaction mixture consisting of **Trastuzumab** (20 µM final concentration, 1 equiv.) and **5’-BA-ss11-mer** (200 µM final concentration, 10 equiv.) in 14 µL of 50 mM NaPi pH 8.0 was vortexed and shaken at 37 ºC for 1 h. Unreacted ON was removed by Zeba™ Micro Spin Desalting Column 7K MWCO and antibody disufide bonds were reduced prior to the LC-MS analysis according to the general pre-LC-MS reduction method. 5 µL aliquot of the reaction mixture was then analyzed by LC-MS and no light or heavy chain modification was observed.

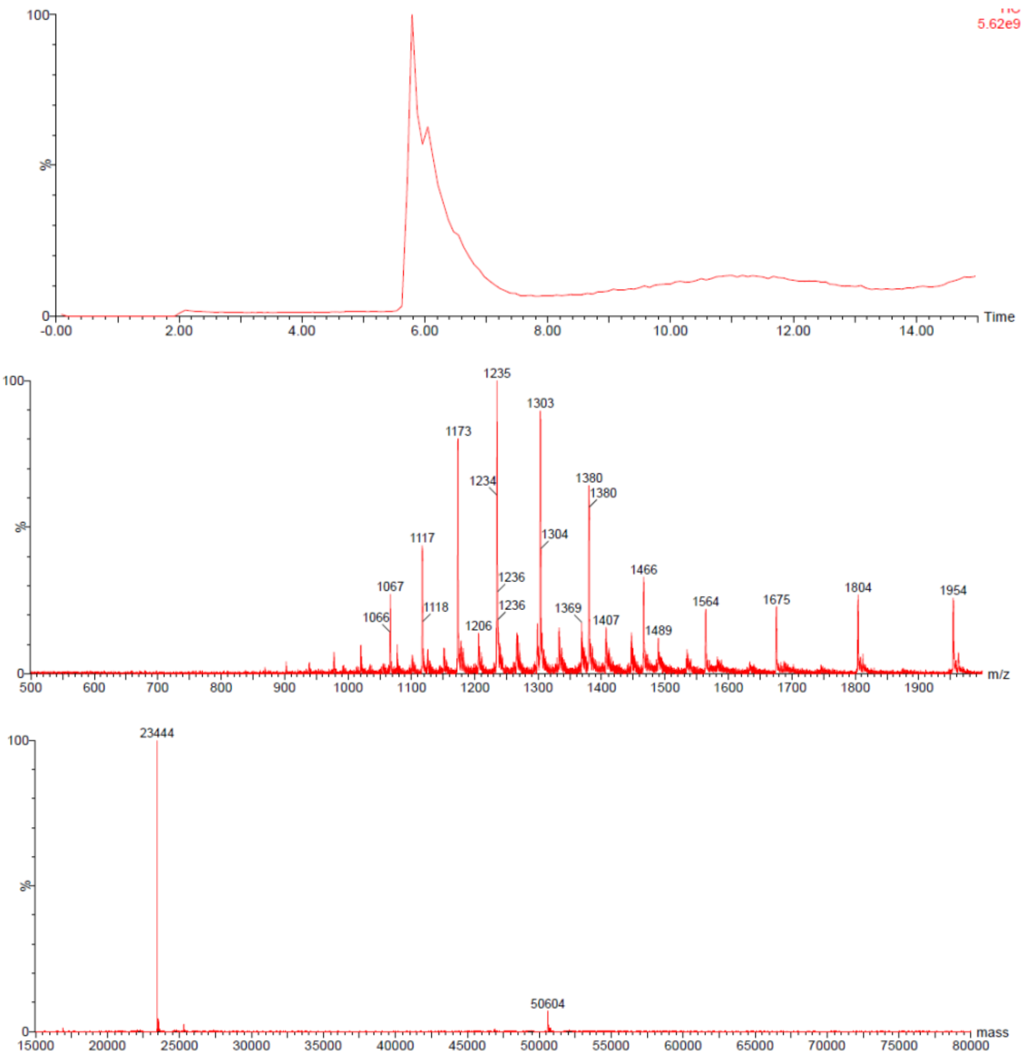


**Figure S53.** LC-MS analysis of the reaction mixture from the reaction between **Trastuzumab** and **5'-BA-ss11-mer**.

**2Rb17c-C138-ss11-mer**

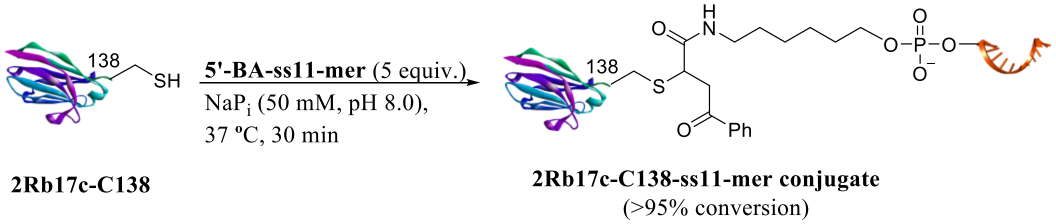


In an Eppendorf tube, the reaction mixture consisting of the freshly reduced **2Rb17c-C138** (20 µM final concentration, 1 equiv.) and **5'-BA-ss11-mer** (100 µM final concentration, 5 equiv.) in 80 µL of 50 mM $NaP_i$ pH 8.0 was vortexed and shaken at 37 ºC for 30 min. 5 µL aliquot of the reaction mixture was then analyzed by LC-MS and complete conversion of the starting material to the desired **2Rb17c-C138-ss11-mer** conjugate product was obtained (calculated: 18488 Da; observed: 18484 Da).

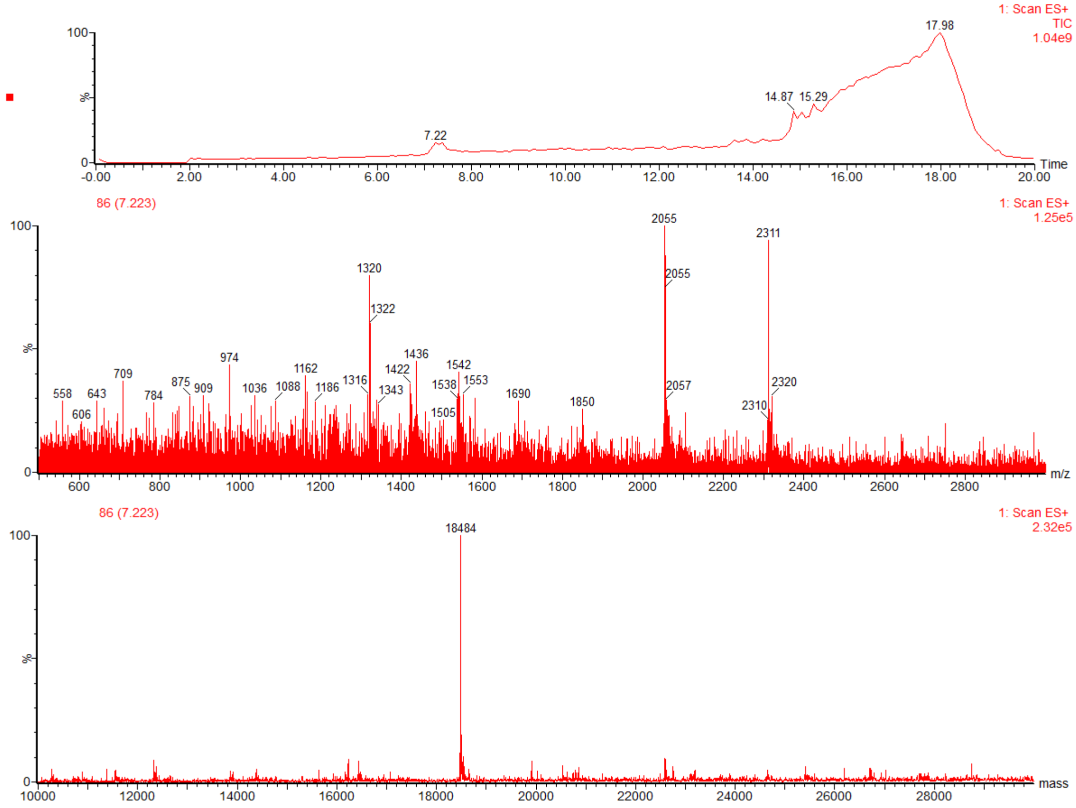


**Figure S54.** LC-MS analysis of the reaction mixture from the preparation of the **2Rb17c-C138-ss11-mer** conjugate.

**2Rb17c-C138-maleimide-ss11-mer**

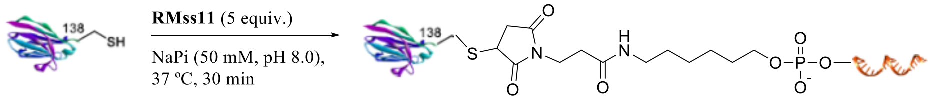


**2Rb17c-C138** **2Rb17c-C138-maleimide-ss11-mer conjugate**
(~64% conversion)

To an Eppendorf tube with **5'-NH$_2$-ss11-mer** ON (50 µM final concentration, 1 equiv.) in 355 µL of 50 mM NaPi pH 8.0, 100 µL of **3-(maleimido)propionic acid *N*-hydroxysuccinimide ester 3** (Mal-NHS ester, 25 mM in DMF, 100 equiv., 22% DMF final concentration) was added. The reaction mixture was vortexed and shaken at 37 ºC, overnight. 5 µL aliquot of the reaction mixture was analysed by LC-MS. Complete conversion of the starting material to ON reaction mixture **RMss11** - 10% **5'-maleimide-ss11-mer** (calculated: 3620 Da; observed: 3622 Da) and 90 % **5'-maleamic acid-ss11-mer** (calculated: 3638 Da; observed: 3641 Da) - was obtained.

In an Eppendorf tube, the reaction mixture consisting of freshly reduced **2Rb17c-C138** (15 µM final concentration, 1 equiv.) and **RMss11** (75 µM final concentration, 5 equiv.) in 14 µL of 50 mM NaPi pH 8.0 was vortexed and shaken at 37 ºC for 30 min. 5 µL aliquot of the reaction mixture was then analyzed by LC-MS and ~ 64 % conversion of the starting material to the desired **2Rb17c-C138-maleimide-ss11-mer** was obtained (calculated: 18481 Da; observed: 18483 Da).

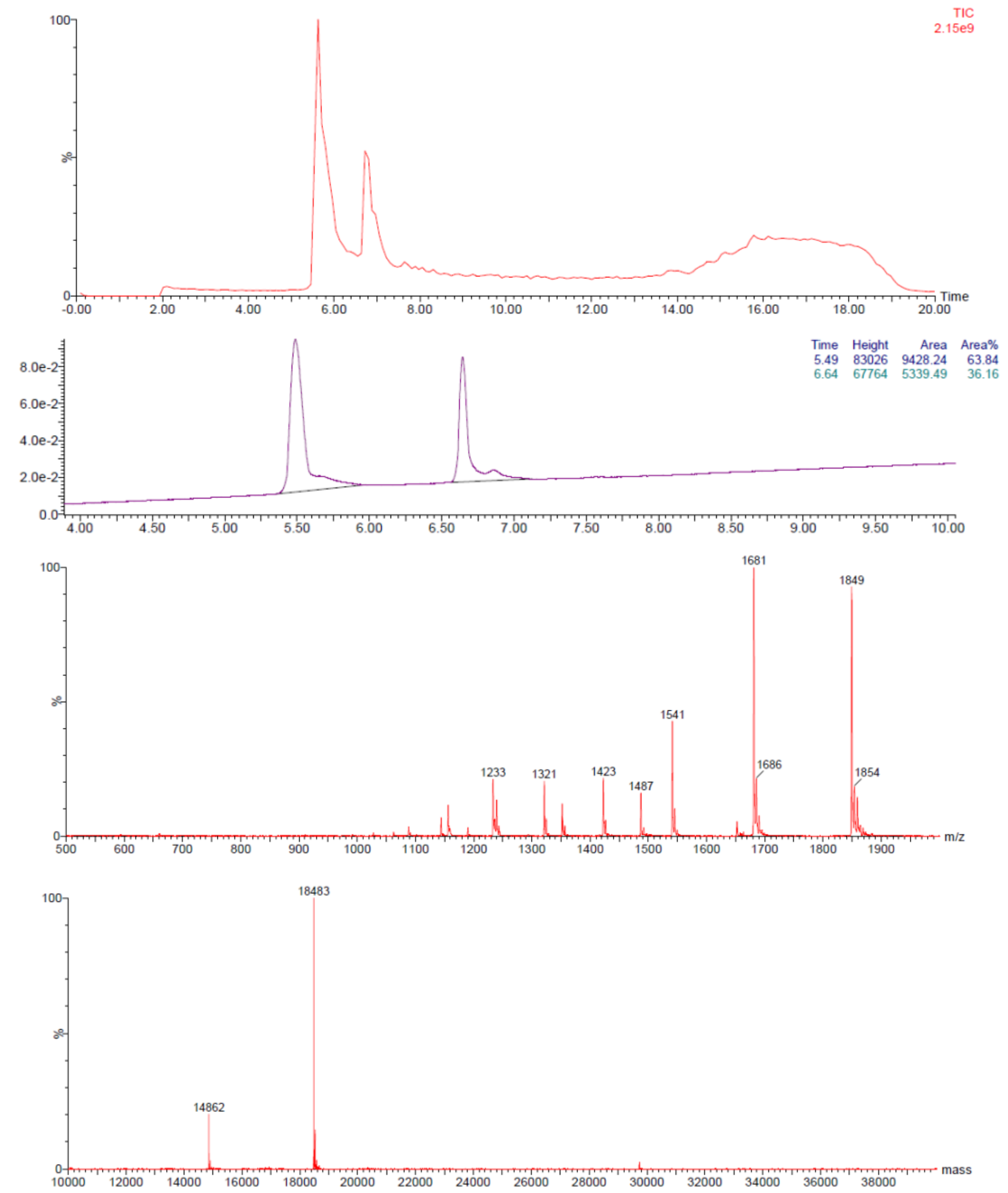


**Figure S55.** LC-MS analysis of the reaction mixture from the preparation of the **2Rb17c-C138-maleimide-ss11-mer** conjugate.

## 2Rb17c-C138-ss25-mer

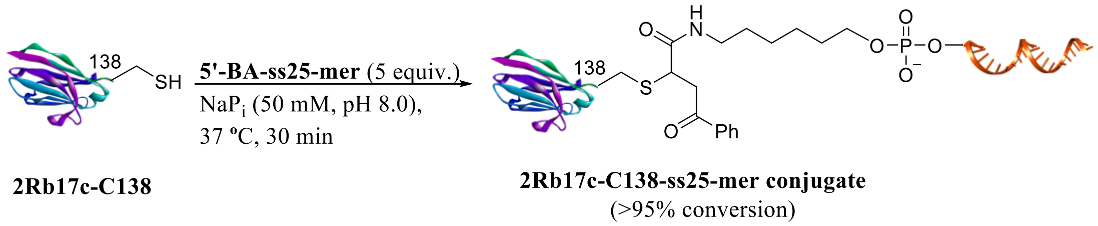


In an Eppendorf tube, the reaction mixture consisting of the freshly reduced **2Rb17c-C138** (20 µM final concentration, 1 equiv.) and **5'-BA-ss25-mer** (100 µM final concentration, 5 equiv.) in 20 µL of 50 mM $NaP_i$ pH 8.0 was vortexed and shaken at 37 ºC for 30 min. 5 µL aliquot of the reaction mixture was then analyzed by LC-MS and complete conversion of the starting material to the desired **2Rb17c-C138-ss25-mer** conjugate product was obtained (calculated: 22859 Da; observed: 22858 Da).

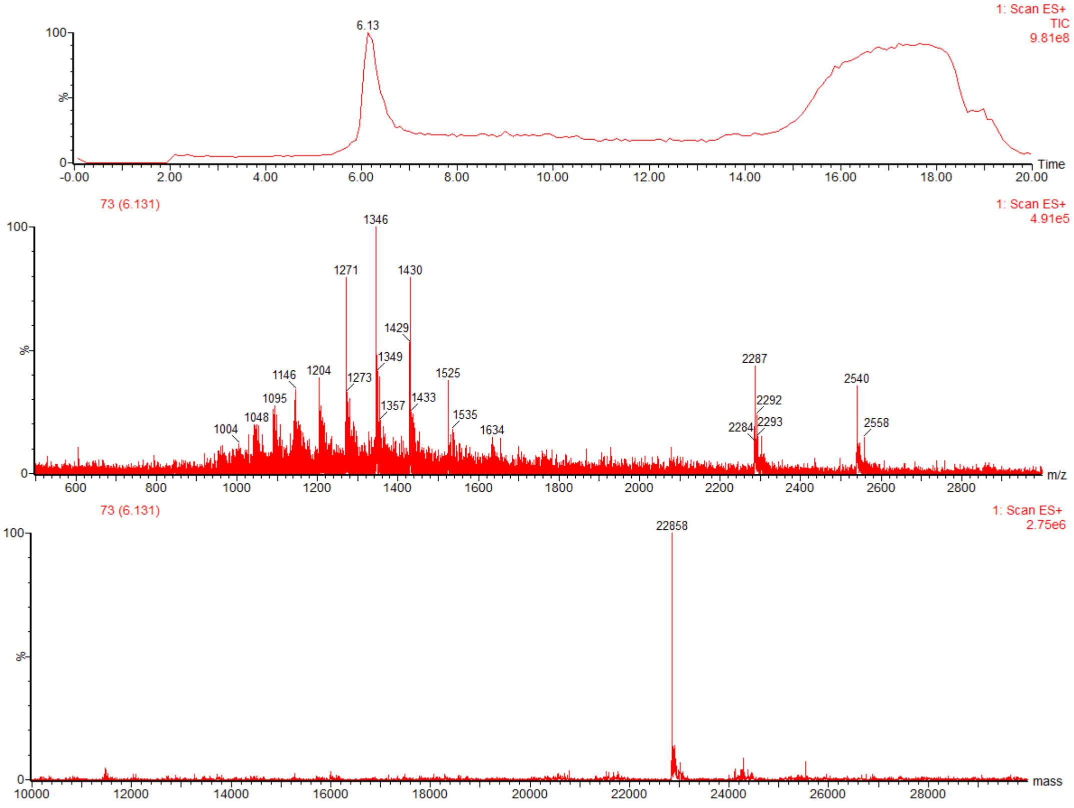


**Figure S56.** LC-MS analysis of the reaction mixture from the preparation of the **2Rb17c-C138-ss25-mer** conjugate.

## 2Rb17c-C138-ss50-mer

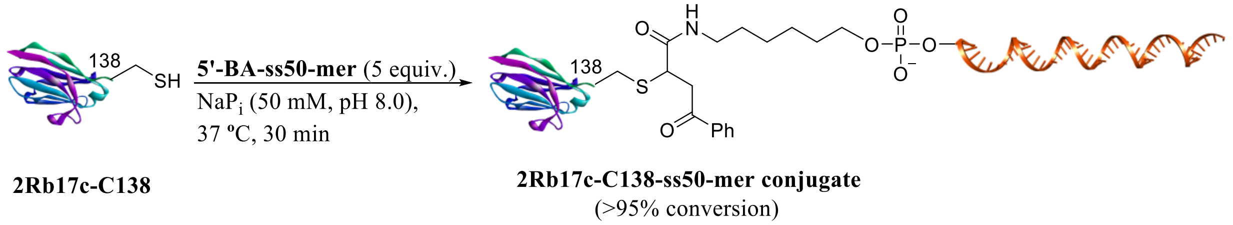


In an Eppendorf tube, the reaction mixture consisting of the freshly reduced **2Rb17c-C138** (13 μM final concentration, 1 equiv.) and **5'-BA-ss50-mer** (65 μM final concentration, 5 equiv.) in 32 μL of 50 mM $NaP_i$ pH 8.0 was vortexed and shaken at 37 ºC for 30 min. 5 μL aliquot of the reaction mixture was then analyzed by LC-MS and complete conversion of the starting material to the desired **2Rb17c-C138-ss50-mer** conjugate product was obtained (calculated: 30544 Da; observed: 30548 Da).

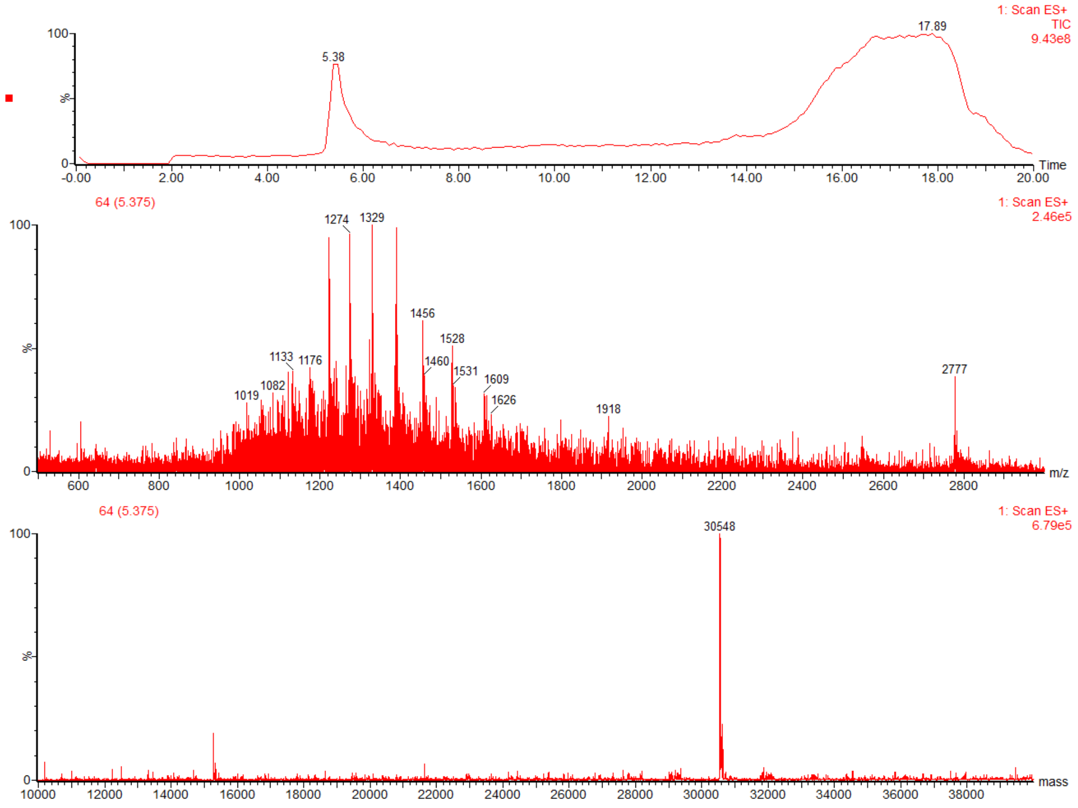


**Figure S57.** LC-MS analysis of the reaction mixture from the preparation of the **2Rb17c-C138-ss50-mer** conjugate.

## 2Rb17c-C138-ds19-mer

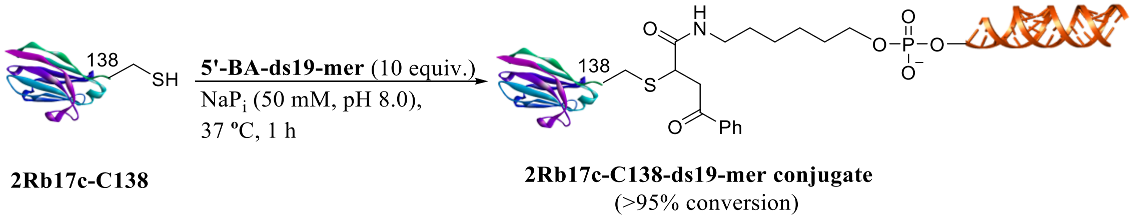


In an Eppendorf tube, the reaction mixture consisting of the freshly reduced **2Rb17c-C138** (20 μM final concentration, 1 equiv.) and **5'-BA-ds19-mer** (200 μM final concentration, 10 equiv.) in 20 μL of 50 mM NaP$_i$ pH 8.0 was vortexed and shaken at 37 ºC for 1 h. 5 μL aliquot of the reaction mixture was then analyzed by LC-MS and complete conversion of the starting material to the desired **2Rb17c-C138-ds19-mer** conjugate product was obtained (calculated with the sense strand: 20995 Da; observed: 20990 Da).

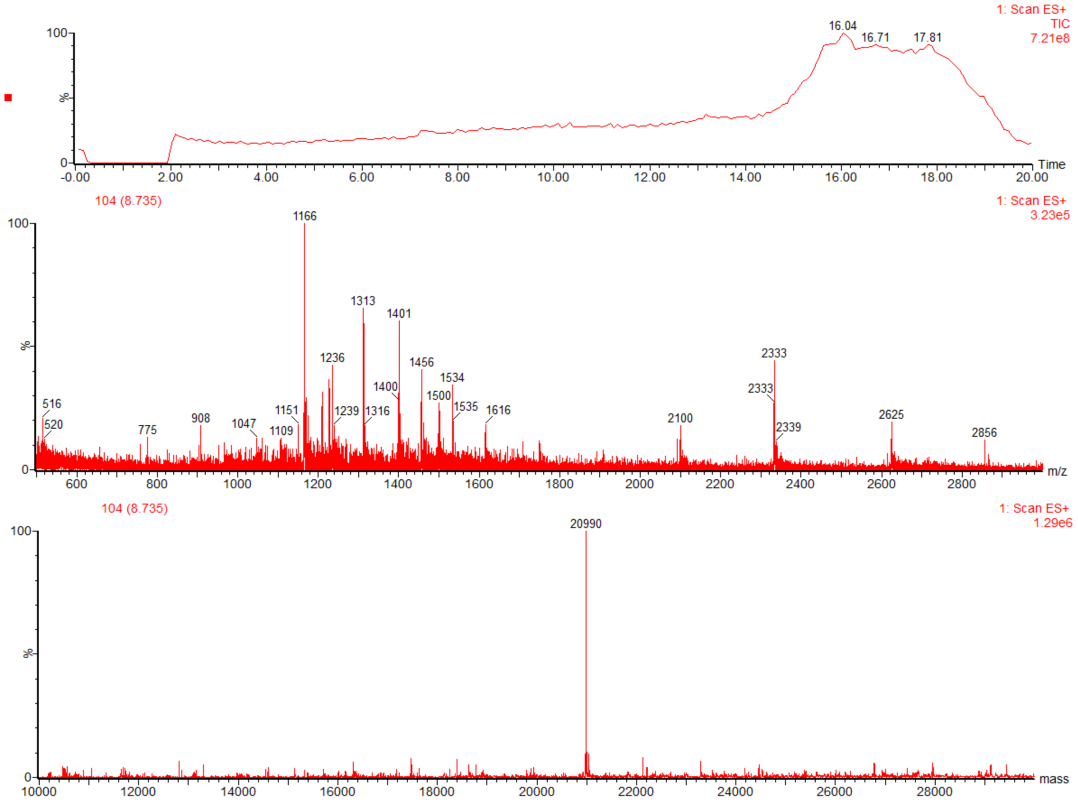


**Figure S58.** LC-MS analysis of the reaction mixture from the preparation of the **2Rb17c-C138-ds19-mer** conjugate.

## 2Rb17c-C138-Cy3-3'-ss25-mer

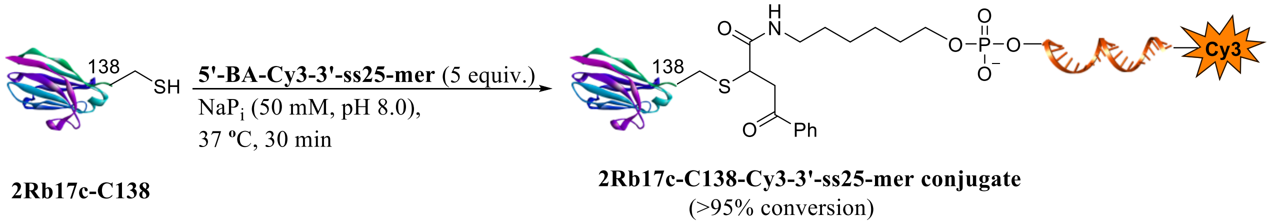


In an Eppendorf tube, the reaction mixture consisting of the freshly reduced **2Rb17c-C138** (20 µM final concentration, 1 equiv.) and **5'-BA-Cy3-3'-ss25-mer** (200 µM final concentration, 10 equiv.) in 15 µL of 50 mM $NaP_i$ pH 8.0 was vortexed and shaken at 37 ºC for 1 h. 5 µL aliquot of the reaction mixture was then analyzed by LC-MS and complete conversion of the starting material to the desired **2Rb17c-C138-Cy3-3'-ss25-mer** conjugate product was obtained (calculated: 23504 Da; observed: 23505 Da).

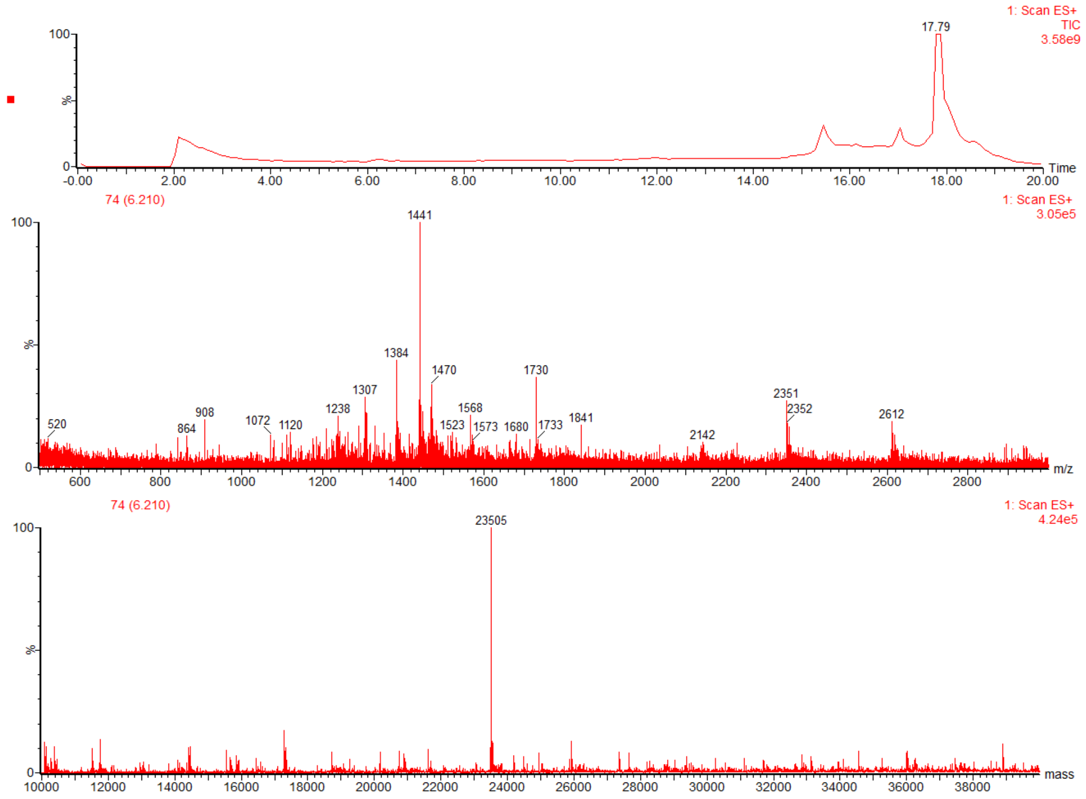


**Figure S59.** LC-MS analysis of the reaction mixture from the preparation of the **2Rb17c-C138-Cy3-3'-ss25-mer** conjugate.

Each DNA-protein or antibody bioconjugation reaction was repeated at least once under specified optimized reaction conditions and similar results were obtained for each construct.

## Plasma integrity tests of the 2Rb17c-C138-ss11-mer conjugate

In an Eppendorf tube, lyophilized **2Rb17c-C138-ss11-mer** (~40 µM final concentration) was redissolved in 45 µL of phosphate-buffered saline buffer pH 7.4 containing 10% human plasma (Sigma-Aldrich, P9523), vortexed and shaken at 37 ºC. 5 µL aliquots of the sample were taken right upon redissolving and after 24 h, 48 h, 72 h and analyzed by LC-MS. No detectable degradation of the **2Rb17c-C138-ss11-mer** was observed by LC-MS (calculated: 18488 Da; observed: 18486 Da or 18484 Da).

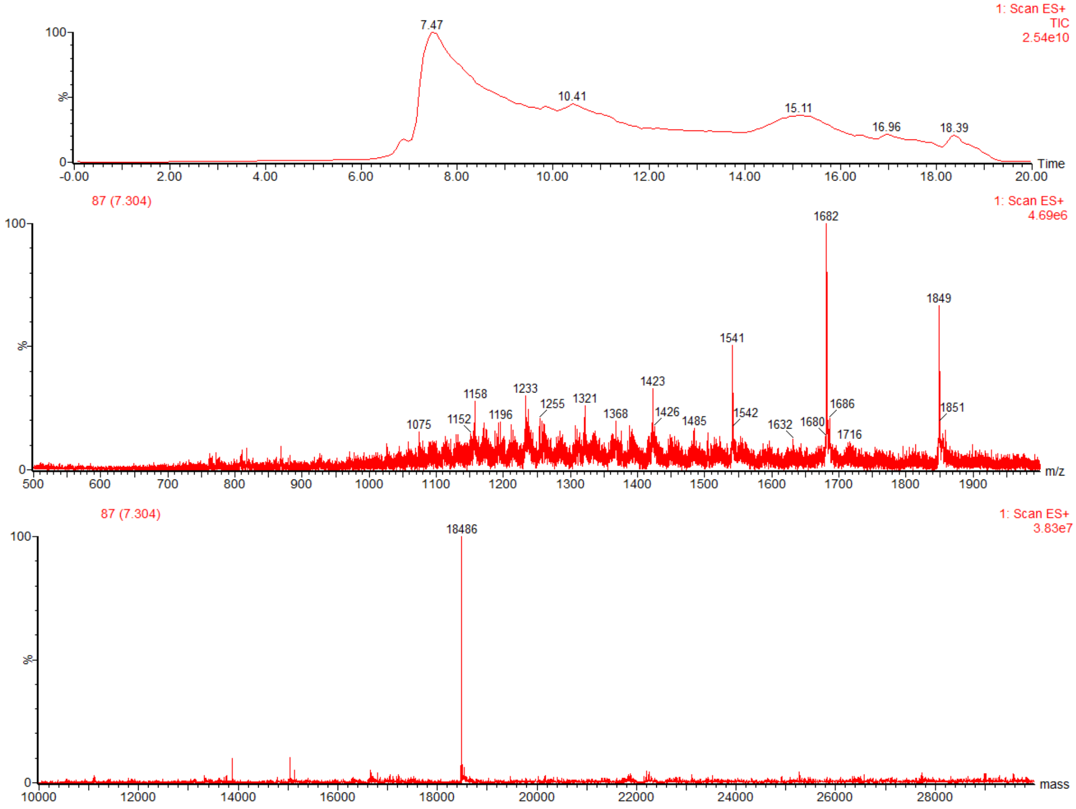


**Figure S60.** LC-MS analysis of the sample (right upon redissolving) from the plasma stability testing of the **2Rb17c-C138-ss11-mer** conjugate.

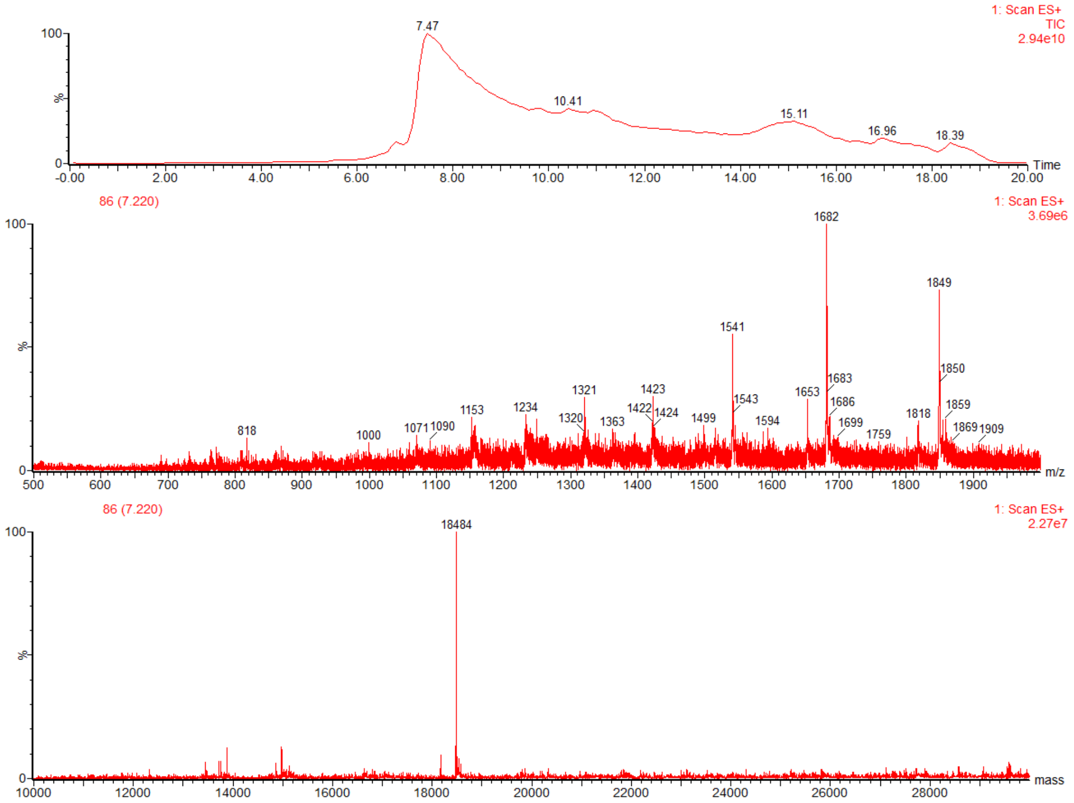


**Figure S61.** LC-MS analysis of the sample (after 24 h) from the plasma stability testing of the **2Rb17c-C138-ss11-mer** conjugate.

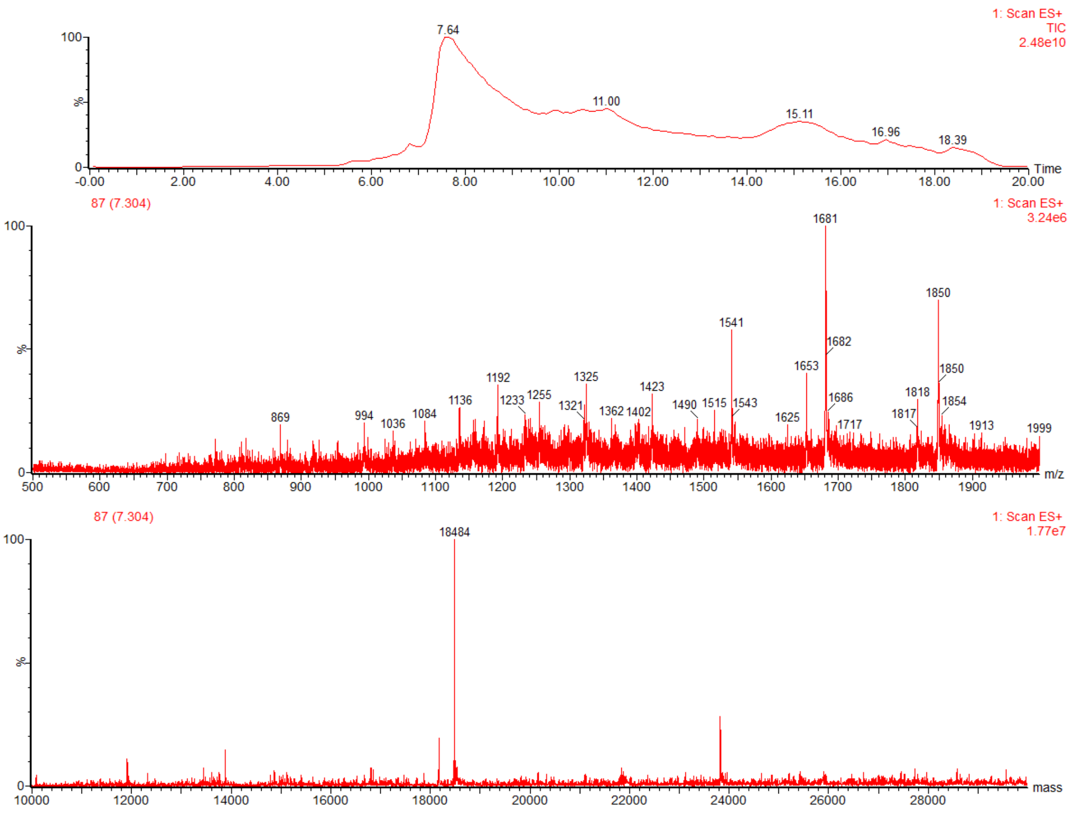


**Figure S62.** LC-MS analysis of the sample (after 48 h) from the plasma stability testing of the **2Rb17c-C138-ss11-mer** conjugate.

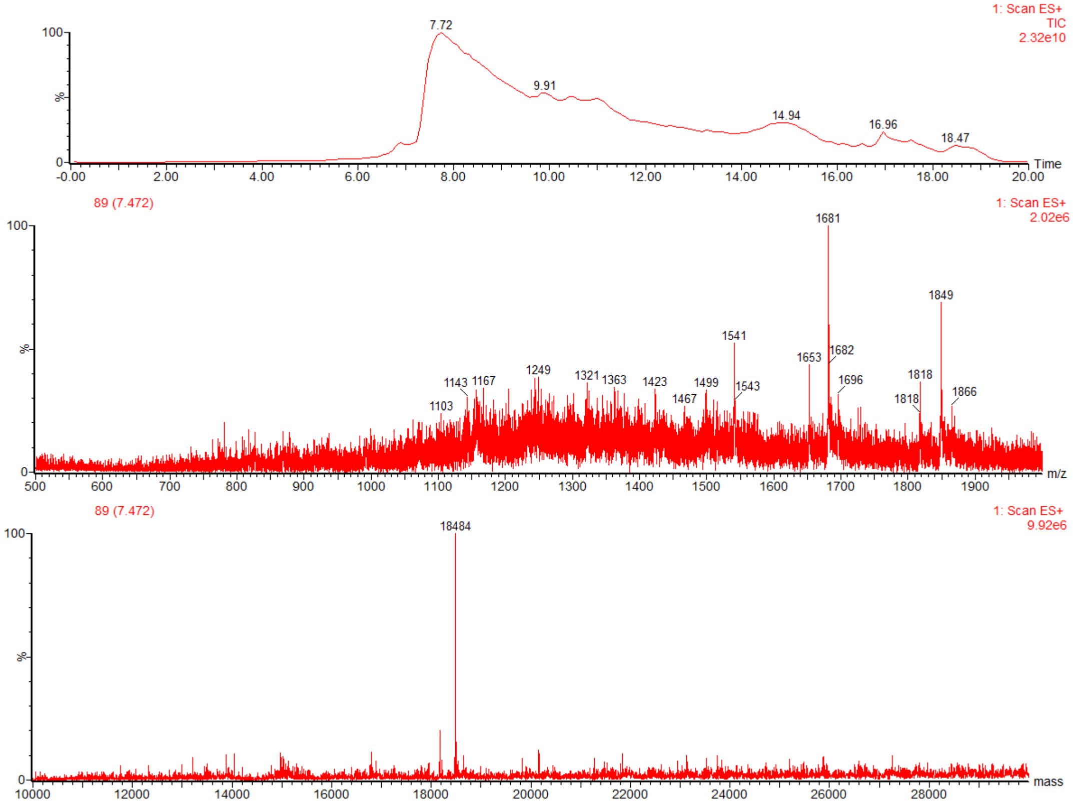


**Figure S63.** LC-MS analysis of the sample (after 72 h) from the plasma stability testing of the **2Rb17c-C138-ss11-mer** conjugate.

## Cell culture

SK-BR-3 [SKBR3] (ATCC® HTB-30™) cells were grown in a humidified incubator at 37 ºC under 5% $CO_2$ with 90% humidity and split before reaching confluence using 0.25% Trypsin-EDTA solution. Cells were grown in McCoy's 5A (Modified) Medium, GlutaMAX™ Supplement, supplemented with 10% heat-inactivated FBS, 100 units/mL penicillin and 100 µg/mL streptomycin. For imaging experiments, cells were grown to >90% confluency before seeding 50000 cells/well into Corning Costar® 6-well clear flat bottom plates containing EtOH-sterilized 25mm round glass coverslips or 30000 cells/well into ibidi µ-slide 8 well chamber slides. Before each plating, cell count was determined by Trypan Blue Exclusion assay using Countess™ II Automated Cell Counter (Thermo Fisher Scientific, UK). All cell culture reagents were purchased from Gibco Life Technologies (Thermo Fisher Scientific, UK), unless stated otherwise.

## DNA-PAINT imaging experiments

Cells on 25mm round glass coverslips in a Corning Costar® 6-well clear flat bottom plate were incubated at 37 ºC for 24 h before fixation. Cell culture medium was removed, cells were briefly washed with PBS and fixed with 4% paraformaldehyde in PBS for 10 min. Cells were then briefly washed with PBS (3×), permeabilized with 0.25% Triton X-100 in PBS for 10 min, briefly washed with PBS (3×) and blocking was performed for 1 h with 3% bovine serum albumin, 0.1% Triton X-100 and 0.5 mg/mL Salmon Sperm DNA in PBS. Next, cells were briefly washed with PBS and stained for 2 h with **Thiomab LC-V205C-ss11-mer** (15 µg/mL) or **2Rb17c-C138-ss11-mer** (1.5 µg/mL) in 3% bovine serum albumin and 1 mg/mL Salmon Sperm DNA in PBS. After this, cells were washed with PBS (3×) with 5 min incubation each time before being imaged using near-TIRF illumination with 5 nM imaging strand as previously described.[12] Briefly, imaging was performed on a home-built total internal reflection fluorescence (TIRF) microscope. Light from a 561 nm laser (Cobalt Jive, Cobalt) was circularly polarized, expanded and passed through a FF01-561/14-25 excitation filter before being focussed on the back of 1.49 N.A., 60x TIRF objective (UPLSAPO, 60XO TIRF, Olympus) mounted on an inverted Ti-E Eclipse microscope (Nikon, Japan). Fluorescence was collected by the objective and separated from the excitation light by a dichroic mirror (Di01- R405/488/561/635, Semrock), and passed through appropriate filters (BLP01-488R-25 for ThT and LP02-568RS-25 for Cy3B, Semrock). The fluorescence was then passed through a 2.5x beam expander onto an EMCCD camera (Evolve 512, Photometrics) with a 50 ms exposure. Each pixel corresponded to a length of 104.2 nm. Localizations were detected using the PeakFit plugin (an imageJ/Fiji plugin of the GDSC Single Molecule Light Microscopy package www.sussex.ac.uk/gdsc/intranet/microscopy/imagej/gdsc_plugins) for imageJ[13] using a 'signal strength' threshold of 30 and a precision threshold of 20 nm. The localizations were sorted into clusters using the DBSCAN algorithm in Python 2.7 (sklearn v0.18.1) using epsilon = 3 pixels and a minimum points threshold of 10 to remove spurious localizations. TatraSpeck™ Microspheres, 0.1 µM, fluorescent, blue/green/orange/dark red (Thermo Fisher Scientific, UK) were used as fiducial markers at 1:1000 dilution. Each experiment was performed two independent times and similar results were obtained each time.

## Epifluorescence microscopy imaging experiments

SKBR3 cells in cell culture medium in ibidi μ-slide 8 well chamber slides were incubated at 37 ºC for 24 h before 2 h incubation with PBS as a vehicle control **2Rb17c-C138** (0.5 μM) as nanobody control or **5'-NH$_2$-Cy3-3'-ss25-mer ON** (0.5 μM) as Cy3-ON control. Cells were incubated for 2 h with **2Rb17c-C138-Cy3-3'-ss25-mer** or **2Rb17c-C138-C5-AF488** probe (0.5 μM) or incubated for 1 h with **Trastuzumab** (7.5 μM) followed by 2 h incubation with **2Rb17c-C138-Cy3-3'-ss25-mer** probe (0.5 μM). Cell culture medium was removed, cells were briefly washed with PBS (3×) and cell nuclei were stained with Hoechst 33342 (5 μg/mL). Cells were then briefly washed with PBS and fixed with 4% paraformaldehyde in PBS for 10 min. Finally, after 3 more PBS washings, cells were imaged on an EVOS M5000 imaging system. DAPI EVOS light cube was used to image nuclei stained by Hoechst 33342, RFP EVOS light cube was used for the imaging of Cy3 conjugates and GFP EVOS light cube for the imaging of the AF488 conjugate. Images were processed using ImageJ software.[13] Each experiment was performed two independent times and similar results were obtained each time. Cell fluorescence was quantified using the data from obtained images and ImageJ software by calculating Corrected total cell fluorescence (CTCF) using the following formula: CTCF = Integrated Density – (Area of selected cell × Mean fluorescence of background readings).

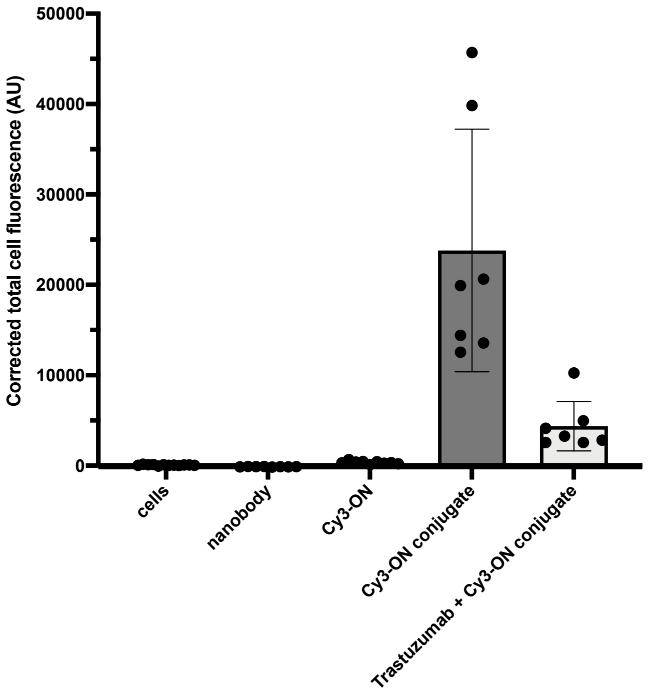


**Figure S64.** Quantification of the intracellular localization of the **2Rb17c-C138-Cy3-3'-ss25-mer** conjugate after 2 h incubation without or with 1 h pre-incubation with Trastuzumab. Bar graph represents CTCF of epifluorescence images from one experiment in the red channel, error bars represent SD.

Preparation of the **2Rb17c-C138-C5-AF488** conjugate

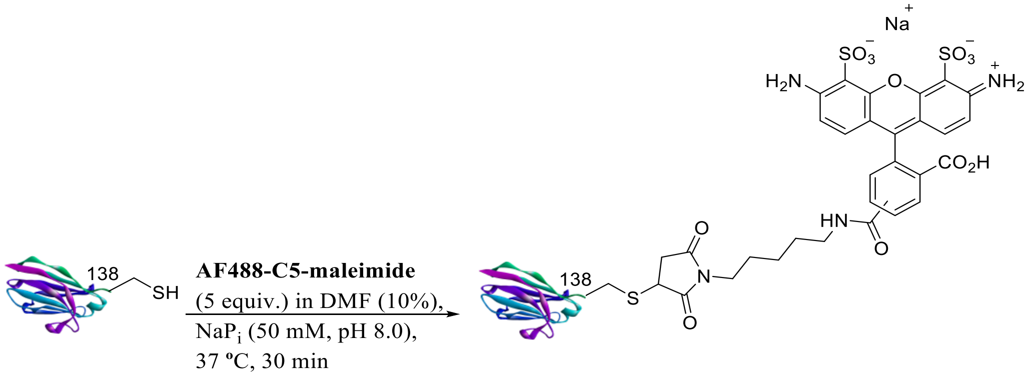


**2Rb17c-C138** **2Rb17c-C138-C5-AF488 conjugate**
(>95 % conversion)

To an Eppendorf tube with a freshly reduced **2Rb17c-C138** (20 µM final concentration, 1 equiv.) in 43.2 µL of 50 mM $NaP_i$ pH 8.0, 4.8 µL of **AF488-C5-maleimide** (1 mM in DMF, 5 equiv., 10% DMF final concentration) were added. The reaction mixture was vortexed and shaken at 37 ºC for 30 min. 5 µL aliquot of the reaction mixture was then analyzed by LC-MS and complete conversion of the starting material to the desired **2Rb17c-C138-C5-AF488** conjugate product was obtained (calculated: 15559 Da; observed: 15561 Da).

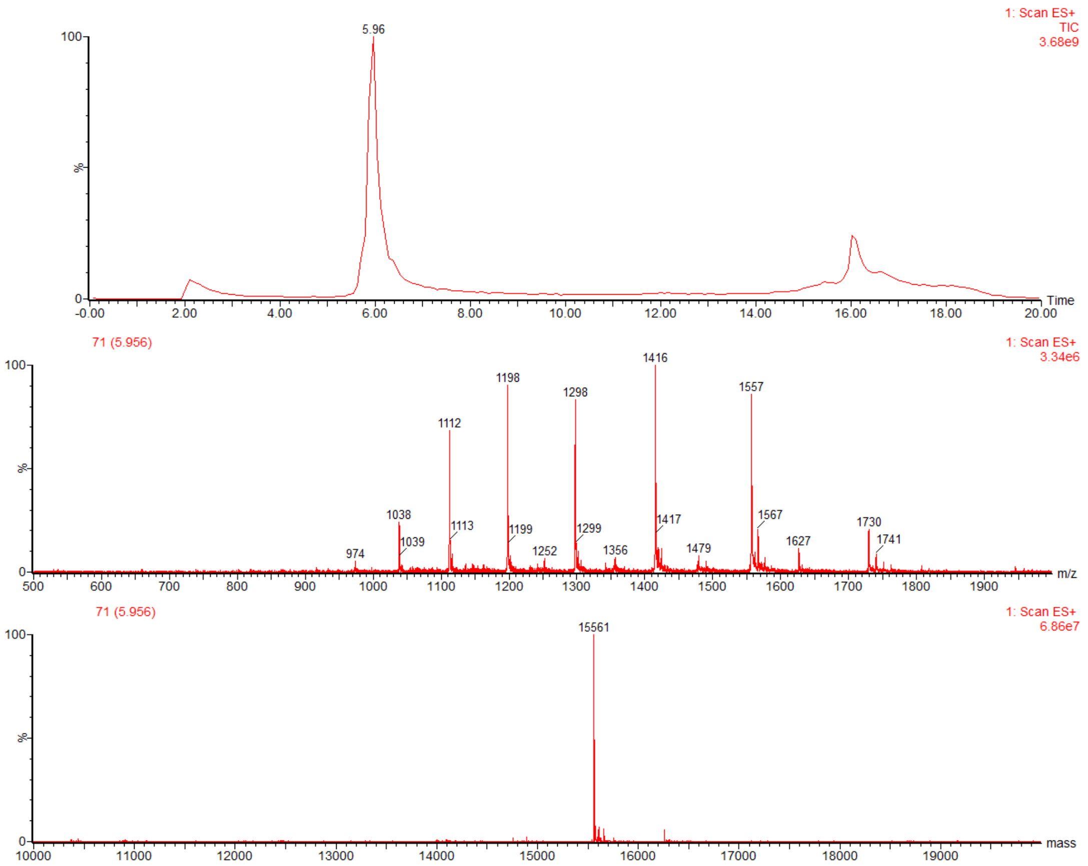


**Figure S65.** LC-MS analysis of the reaction mixture from the preparation of the **2Rb17c-C138-C5-AF488** conjugate.

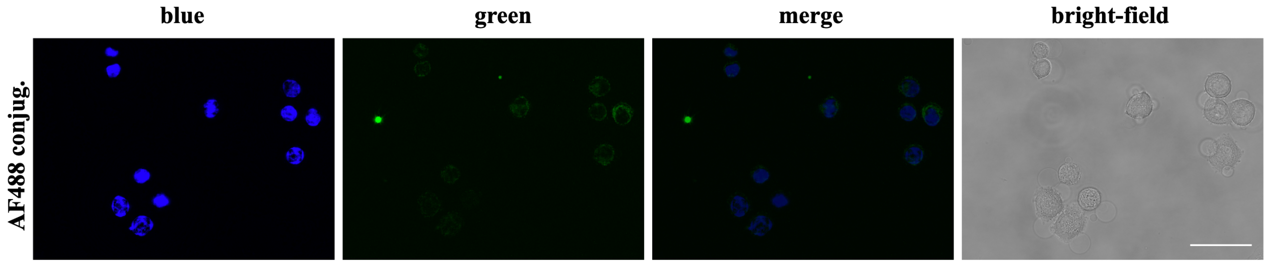


**Figure S66.** Epifluorescence microscopy imaging of the HER2 receptor-mediated internalization of the **2Rb17c-C138-AF488** conjugate on SKBR3 breast cancer cells. DAPI EVOS light cube was used to image nuclei stained by Hoechst 33342 and GFP EVOS light cube for the imaging of the AF488 conjugate. Images of SKBR3 cells were obtained after 2 h incubation with **2Rb17c-C138-C5-AF488** conjugate. Images were processed using ImageJ software,[13] scale bar represents 100 μm.

# NMR spectra

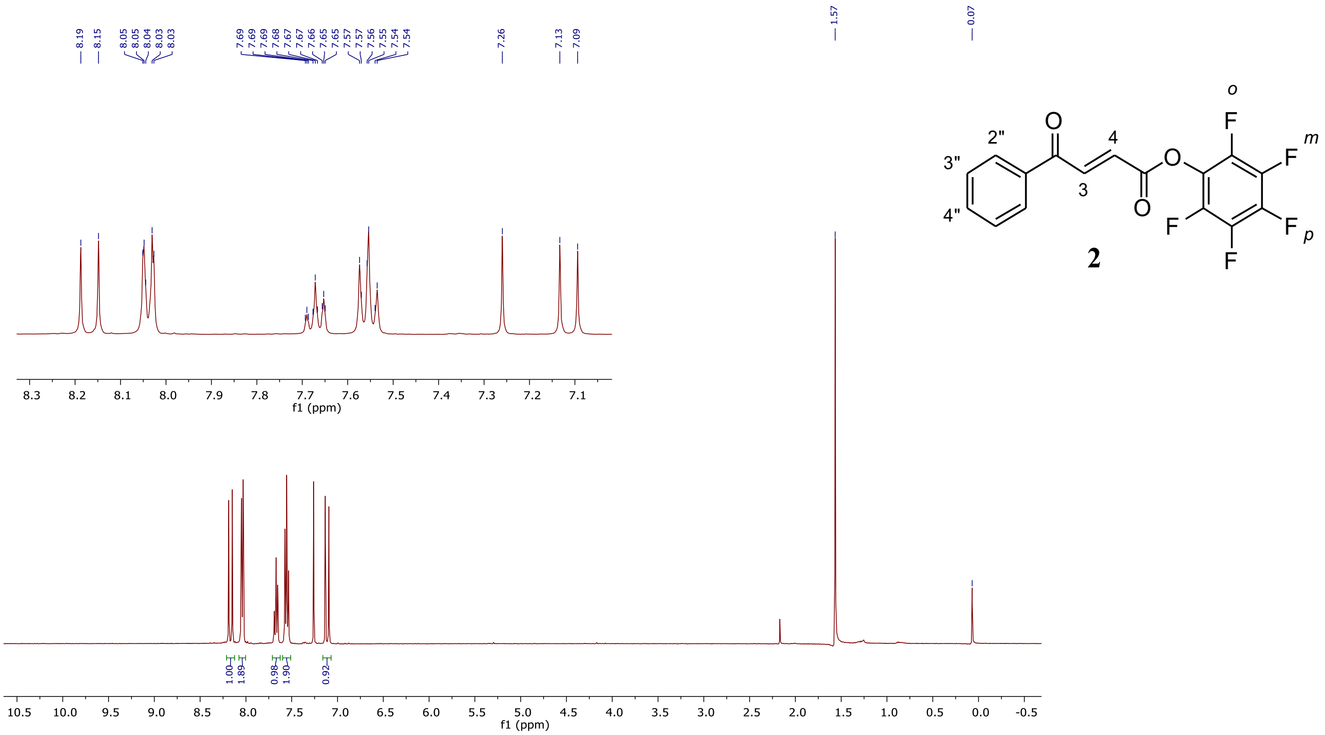


$^{1}$H spectrum of compound **2**.

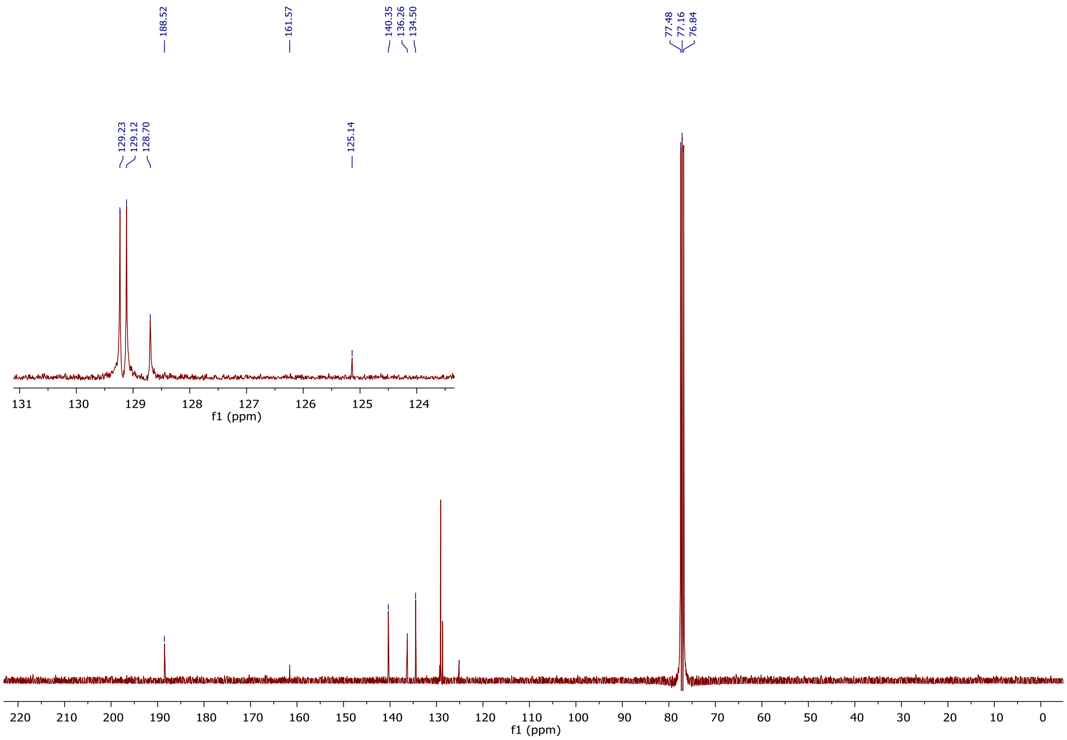


$^{13}$C spectrum of compound **2**.

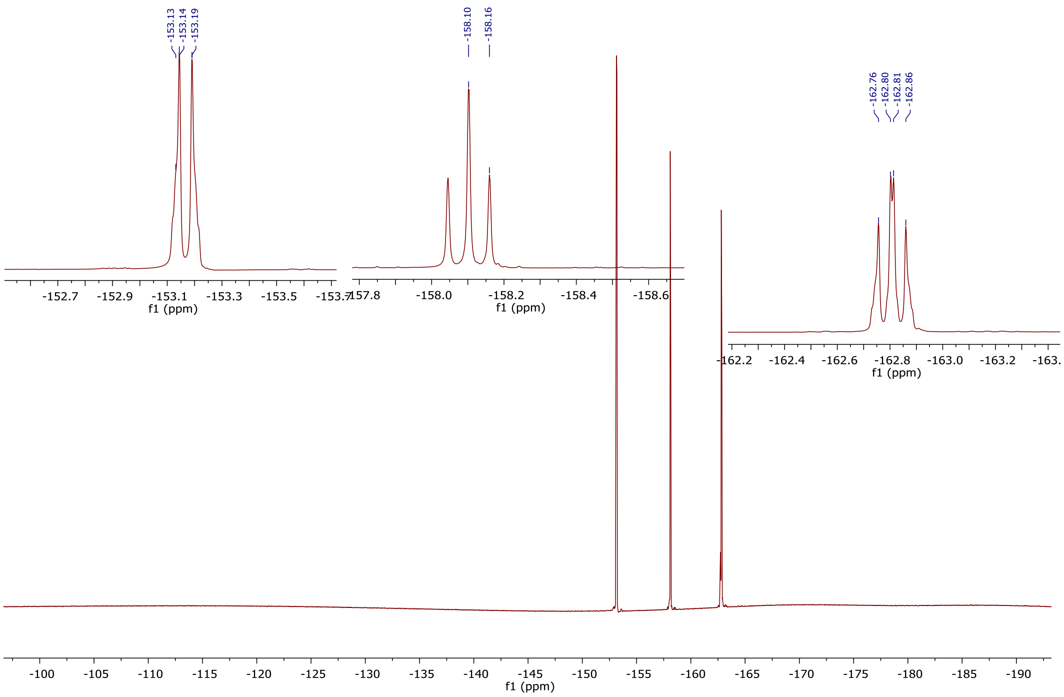


$^{19}$F spectrum of compound **2**.

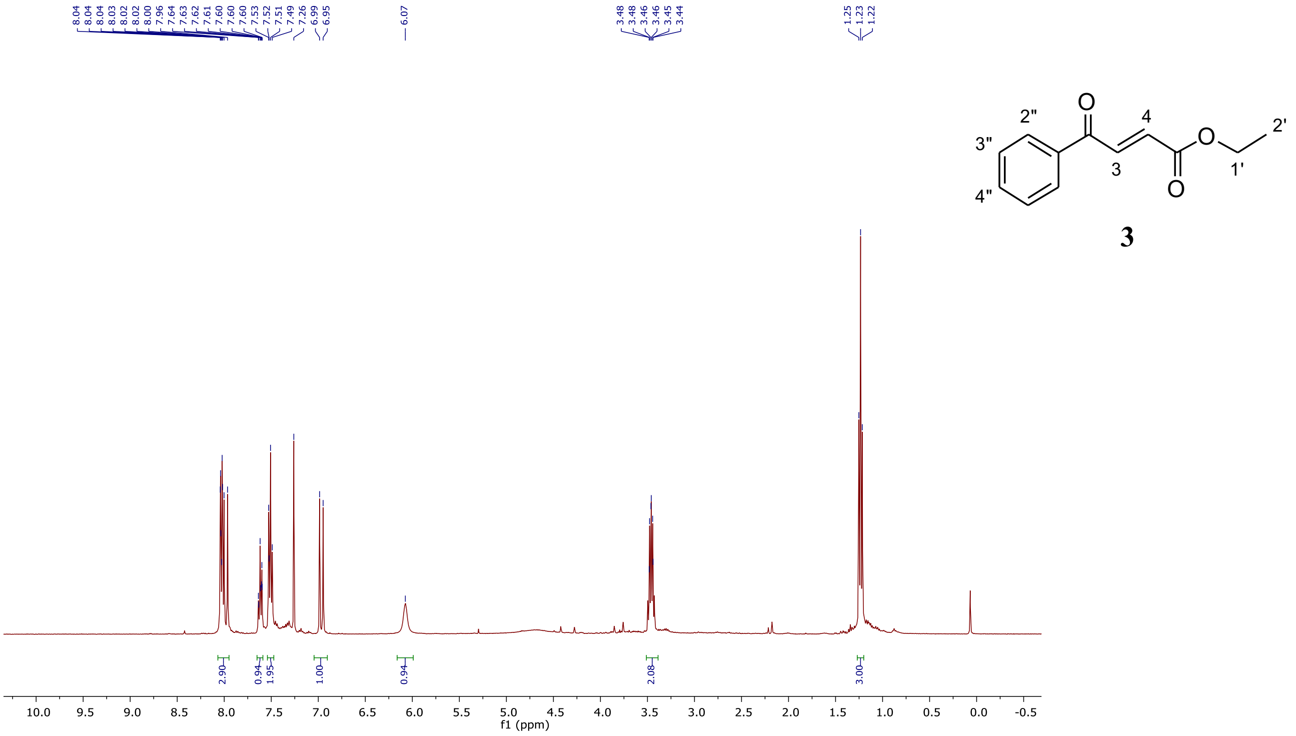


$^{1}$H NMR spectrum of compound **3**.

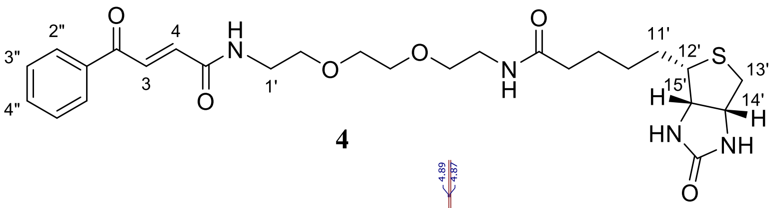

**4**

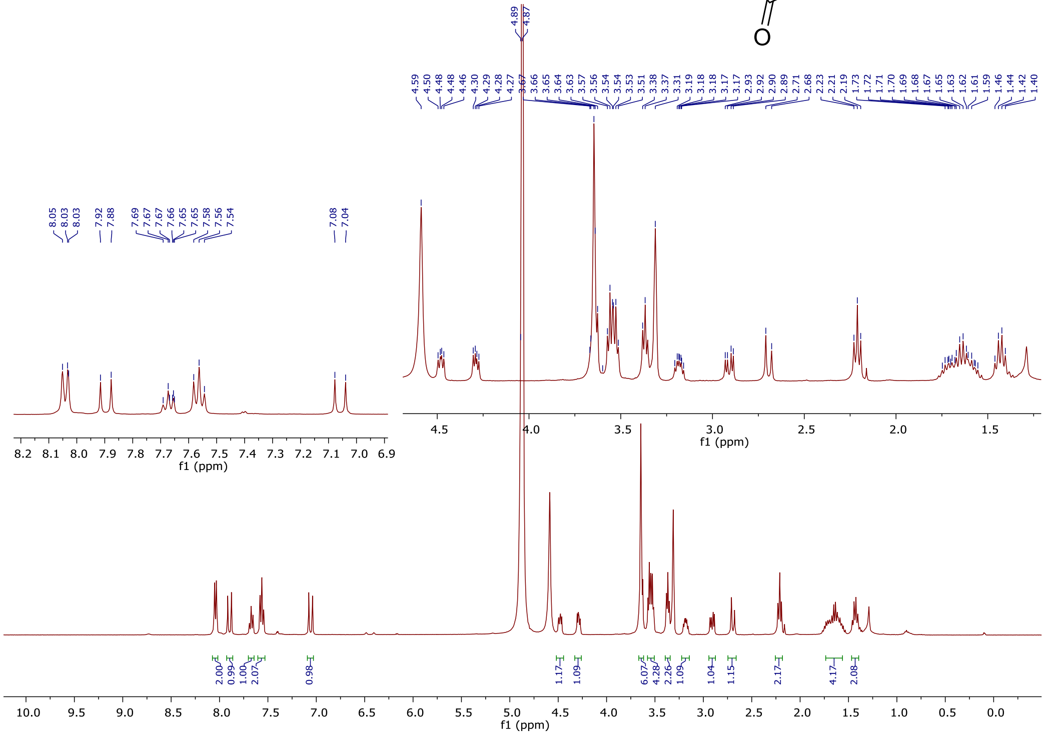


$^{1}H$ spectrum of compound **4**.

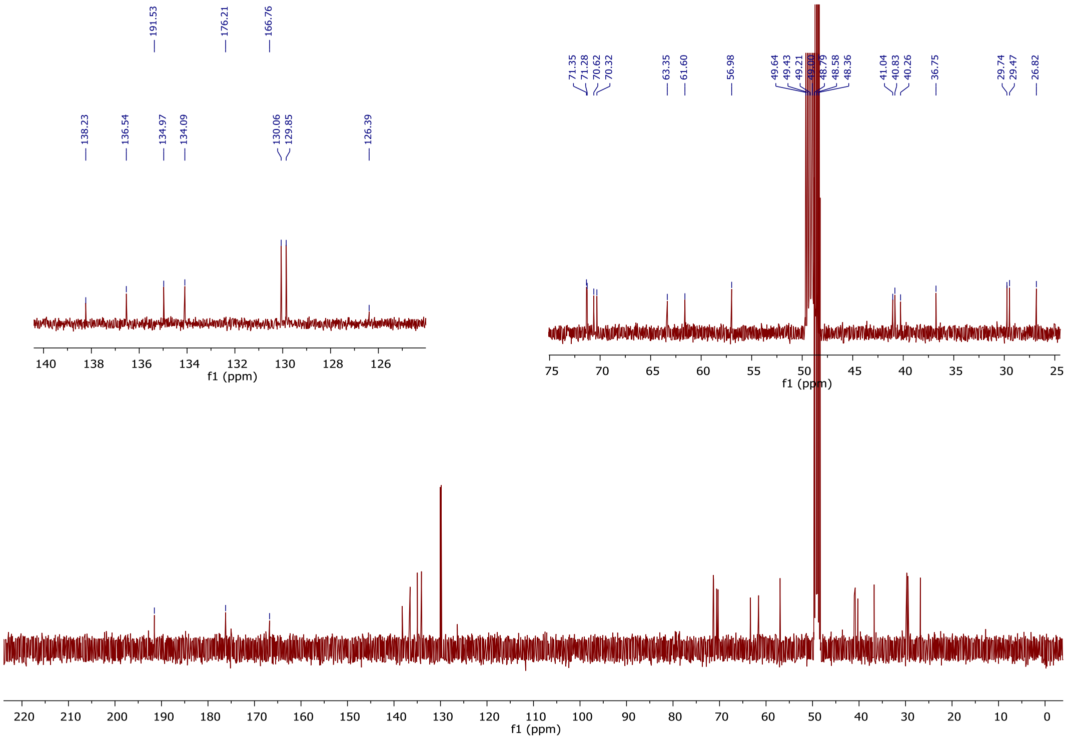


$^{13}C$ spectrum of compound **4**.

# References

1) A. B. Pangborn, M. A. Giardello, R. H. Grubbs, R. K. Rosen, F. J. Timmers, *Organometallics* **1996**, *15*, 1518–1520.

2) DNA Calculator. http://www.molbiotools.com/dnacalculator.html Accessed [30/11/2020].

3) *ProtParam tool.* https://web.expasy.org/protparam/ Accessed [18/07/2020].

4) B. Bernardim, P. M. S. D. Cal, M. J. Matos, B. L. Oliveira, N. Martínez-Sáez, I. S. Albuquerque, F. Corzana, A. C. B. Burtoloso, G. Jiménez-Osés, G. J. L. Bernardes, *Nat. Commun.* **2016**, *7*, 13128.

5) J. Liu, Q. Chen, S. Rozovsky, *J. Am. Chem. Soc.* **2017**, *139*, 3430–3437.

6) I. S. Alam, A. A. Neves, T. H. Witney, J. Boren, K. M. Brindle, *Bioconjugate Chem.* **2010**, *21*, 884–891.

7) S. E. Logue, M. Elgendy, S. J. Martin, *Nat. Protoc.* **2009**, *4*, 1383–1395.

8) T. Pleiner, M. Bates, S. Trakhanov, C. -T. Lee, J. E. Schliep, H. Chug, M. Böhning, H. Stark, H. Urlaub, D. Görlich, *eLife* **2015**, *4*, e11349.

9) I. Vaneycken, N. Devoogdt, N. Van Gassen, C. Vincke, C. Xavier, U. Wernery, S. Muyldermans, T. Lahoutte, V. Caveliers, *FASEB J.* **2011**, *25*, 2433–2446.

10) L. Konermann, *J. Am. Soc. Mass Spectrom.* **2017**, *28*, 1827–1835.

11) B. Bernardim, M. J. Matos, X. Ferhati, I. Compañón, A. Guerreiro, P. Akkapeddi, A. C. B. Burtoloso, G. Jiménez-Osés, F. Corzana, G. J. L. Bernardes, *Nat. Protoc.* **2019**, *14*, 86–99.

12) D. R. Whiten, Y. Zuo, L. Calo, M. -L. Choi, S. De, P. Flagmeier, D. C. Wirthensohn, F. Kundel, R. T. Ranasinghe, S. E. Sanchez, D. Athauda, S. F. Lee, C. M. Dobson, S. Gandhi, M. -G. Spillantini, D. Klenerman, M. H. Horrocks, *ChemBioChem* **2018**, *19*, 2033–2038.

13) ImageJ Image Processing and Analysis in Java. https://imagej.nih.gov/ij/ Accessed [18/07/2020]
